# Cognitive performance during adulthood in a rat model of neonatal diffuse white matter injury

**DOI:** 10.1007/s00213-021-06053-w

**Published:** 2022-01-22

**Authors:** E. J. Marijke Achterberg, Ralf J. van Oldeniel, Erik van Tilborg, Jeroen P. H. Verharen, Cora H. Nijboer, Louk J. M. J. Vanderschuren

**Affiliations:** 1 Department of Population Health Sciences, Unit Animals in Science and Society, Division of Behavioural Neuroscience, Faculty of Veterinary Medicine, Utrecht University, Yalelaan 2, 3584CM Utrecht, The Netherlands; 2 Department for Developmental Origins of Disease, University Medical Center, Utrecht Brain Center, Wilhelmina Children’s Hospital, Utrecht University, Lundlaan 6, 3584EA Utrecht, The Netherlands; 3 Helen Wills Neuroscience Institute, Department of Molecular and Cell Biology, University of California Berkeley, Berkeley, CA USA

**Keywords:** Diffuse white matter injury, Cognition, Attention, Impulsivity, Flexibility, Myelination, Prefrontal cortex

## Abstract

**Rationale:**

Infants born prematurely risk developing diffuse white matter injury (WMI), which is associated with impaired cognitive functioning and an increased risk of autism spectrum disorder. Recently, our rat model of preterm diffuse WMI induced by combined fetal inflammation and postnatal hypoxia showed impaired motor performance, anxiety-like behaviour and autism-like behaviour in juvenile rats, especially males. Immunohistochemistry showed delayed myelination in the sensory cortex and impaired oligodendrocyte differentiation.

**Objective:**

To assess long-term cognitive deficits in this double-hit rat model of diffuse WMI, animals were screened on impulsivity, attention and cognitive flexibility in adulthood using the 5-choice serial reaction time task (5CSRTT) and a probabilistic reversal learning task, tests that require a proper functioning prefrontal cortex. Thereafter, myelination deficits were evaluated by immunofluorescent staining in adulthood.

**Results:**

Overall, little effect of WMI or sex was found in the cognitive tasks. WMI animals showed subtle differences in performance in the 5CSRTT. Manipulating 5CSRTT parameters resulted in performance patterns previously seen in the literature. Sex differences were found in perseverative responses and omitted trials: female WMI rats seem to be less flexible in the 5CSRTT but not in the reversal learning task. Males collected rewards faster in the probabilistic reversal learning task. These findings are explained by temporally rather than permanently affected myelination and by the absence of extensive injury to prefrontal cortical subregions, confirmed by immunofluorescent staining in both adolescence and adulthood.

**Conclusion:**

This rat model of preterm WMI does not lead to long-term cognitive deficits as observed in prematurely born human infants.

**Supplementary Information:**

The online version contains supplementary material available at 10.1007/s00213-021-06053-w.

## Introduction

Diffuse white matter injury (WMI) is the most common type of brain injury in infants born prematurely (Back and Miller 2014). Diffuse WMI is associated with myelination deficits, reduced white matter volumes, punctate white matter lesions and active gliosis, causing sensory, behavioural and cognitive problems that persist throughout adulthood (Back and Miller 2014; Buser et al. 2012; Hack et al. 2002; Kroll et al. 2017; Rutherford et al. 2010). Additionally, preterm birth is associated with increased risk of neurodevelopmental disorders, including autism spectrum disorders (ASD), schizophrenia and attention deficits (Breeman et al. 2016; Joseph et al. 2017; Nosarti et al. 2012; Pritchard et al. 2016). However, to what extent the increased risk of neurodevelopmental disorders in preterm infants is mediated by white matter changes remains incompletely understood.

Important risk factors for white matter abnormalities in preterm infants are inflammatory insults and disrupted cerebral oxygenation. Inflammation in the preterm brain is often the result of maternal complications leading to fetal exposure to inflammation, postnatal infections or diseases of prematurity (e.g. bronchopulmonary dysplasia, necrotizing enterocolitis) whereas disrupted oxygenation resides from underdeveloped cerebral vascularization, lung disease and ventilation therapy (Luu et al. 2016; Patra et al. 2017; Van Tilborg et al. 2016). Moreover, exposure to *multiple* preterm birth–related insults has been suggested to be an important etiological factor, with early hits sensitizing the brain to become more vulnerable to subsequent injurious events (Kaindl et al. 2009; Van Steenwinckel et al. 2014). Indeed, clinical data indicate that exposure of the developing brain to multiple hits dramatically increases the risk of WMI (Barnett et al. 2018; Korzeniewski et al. 2014; Leviton et al. 2013).

Currently, no treatment options are available to protect the developing white matter from preterm birth–related insults. Therefore, translational studies are crucial to aid the development of novel therapeutic strategies. Recently, we introduced a novel double-hit rat model of preterm diffuse WMI in which the combination of fetal inflammation and postnatal hypoxia causes impaired oligodendrocyte maturation, delayed cortical myelination, astrogliosis and microglia activation (Van Tilborg et al. 2018a). Moreover, in these juvenile rats with diffuse WMI, we observed impaired motor performance, anxiety-like behaviour, reduced social play behaviour and increased repetitive grooming, reminiscent of an autism-like phenotype (Van Tilborg et al. 2018a). ASD is characterized by impairments in executive functioning, such as impulse control, attention and cognitive flexibility (e.g. Hill 2004). The increased risk of preterm infants to develop neurodevelopmental disorders associated with cognitive problems later in life raises the question whether rats exposed to combined fetal inflammation and postnatal hypoxia display similar deficits in executive functioning.

In the present study, we therefore investigated the consequences of fetal inflammation and postnatal hypoxia on executive functioning in adult male and female rats (age 7–9 months). The 5-choice serial reaction time task (5CSRTT) was used to assess impulse control, attention and cognitive flexibility during adulthood. The probabilistic reversal learning task was utilized as an additional measure of cognitive flexibility. Myelination in prefrontal cortical subregions was determined at postnatal day 30 and after completion of the cognitive tasks. We predicted that adult rats exposed to combined fetal inflammation and postnatal hypoxia would display reduced impulse control, attention problems and impaired cognitive flexibility accompanied by myelination deficits in the prefrontal cortex.

## Methods

### Animals

All procedures were carried out according to Dutch (“Wet op de dierproeven” 1996) and European regulations (Guideline 86/609/EEC) and were approved by the Animal Ethics Committee of Utrecht University.

In total, 60 animals (Wistar rats, Envigo, Horst, The Netherlands) were used in this study, 48 animals were used in the 5CSTT and probabilistic reversal learning task (control animals: *n* = 28 (13 males, 15 females); WMI animals: *n* = 20 (12 males, 8 females)). One female with WMI was taken out of the experiment after the 5CSRTT and did not participate in the reversal learning test because of overgrown teeth. Out of these 48 animals, 8 control and 7 WMI animals that underwent the cognitive tests were selected for analysis of the PFC and sensory cortex at the age of 9 months when the tests were completed (Fig. 3A–G; Fig. 4D,F). Twelve additional animals (6 control and 6 WMI; not subjected to cognitive tests) were sacrificed at P30 to evaluate integrity of the PFC and sensory cortex in this WMI model (Fig. 3H–K; Fig. 4A–E).

Animals were housed in groups at a 12/12-h light/dark cycle (lights on at 7.00AM) and had ad libitum access to water. Beginning at 1 week prior to starting the test of each task, animals were food deprived until the animals were at 85% of their free-feeding weight. They received 35–40 (males) or 40–45 (females) grams of standard chow per kilogram of bodyweight. Training and testing took place at the age of 7–9 months.

### Induction of WMI: a double-hit model

WMI was induced as described previously (Van Tilborg et al. 2018a). Briefly, the mothers of WMI dams received i.p. injections with 100 μg/kg LPS (Lipopolysaccharide: E. Coli O55:B5; L2880; Sigma-Aldrich) on embryonic days (E)18 and E19. Pups were subsequently subjected to hypoxia (8% O_2_) for 140 min at postnatal day (P)4. “Control” rat pups were derived from saline-treated mothers (at E18/E19) and were kept away from the mother for 140 min under normoxic thermo-regulated conditions at P4.

### Immunohistochemistry and analysis

At P30 or after completion of the 5CSRTT at 9 months of age, animals were sacrificed by overdose pentobarbital. Animals were transcardially perfused with PBS followed by 4% paraformaldehyde (PFA) in PBS and brains were dissected. After post-fixation in 4% PFA for 24 h, brains were dehydrated and embedded in paraffin. Coronal paraffin sections of 8 µm were cut at approximately + 0.48 or + 2.20–2.35 mm from bregma. Sections were deparaffinized and rehydrated. Antigen retrieval was performed by heating sections at 95 °C in 10 mM sodium citrate buffer (pH 6.0) for 20 min. Sections were rinsed in PBS/0.025% TritonX-100 (PBST) and blocked with 5% normal goat (MBP) or donkey (CTIP) serum for 30 min at RT. Primary antibodies mouse-anti-myelin basic protein (MBP; 1:1000 in PBS, Sternberger Monoclonals, SMI94) or rat-anti-CTIP (COUP-TF-Interacting Protein 2; a marker for the lower cortical layers V/VI; 1:1000 in PBS, Abcam ab18465) was incubated o/n at 4 °C. Next day, sections were rinsed in PBS and incubated with alexafluor-488-conjugated goat-anti-mouse antibody or alexafluor-594-conjugated donkey-anti-rat antibody (both 1:500; Life Technologies, Carlsbad, CA) for 2 h at 37 °C. After washing in PBS, sections were counterstained with DAPI and embedded with fluorsave.

Full slice images were acquired at 10 × (NA 0.3) with an Olympus IX83 widefield microscope, and stitched by CellSens (multiple image alignment) V3.1.1. Gamma was adjusted for visualization using ImageJ (Figs. 3A, 4A). Micrographs (10 × , 20 ×) from both hemispheres were obtained using a Cell Observer microscope (Zeiss, Oberkochen, Germany). For MBP analysis at approximately + 2.20–2.35 mm from bregma (medial prefrontral cortex (mPFC), Paxinos and Watson 5th edition), Paxinos and Watson (2007) in each hemisphere 3 pictures were taken (20 ×) in the medial prefrontal cortex: dorsal prelimbic cortex (dPrL, L1), ventral prelimbic cortex (vPrL, L2) and the border of the vPrL and infralimbic cortex (vPrL/IL, L3) cortices (see Fig. 3B/C). The following procedure was followed: for dPrL (L1), the microscope field (10 × ; Fig. 3C-L1) was outlined vertically to the midline of the hemispheres with the top of the field aligning the corpus callosum (forceps minor), then the magnification was switched to 20 × and a photograph was taken (square in Fig. 3C-L1). For micrographs of the vPrL (L2), magnification was switched back to 10 × , still vertically aligning the midline of the hemispheres and moving one microscope field down, then switching again to 20 × magnification for a micrograph of L2 (square in Fig. 3C-L2). This procedure was repeated once more for the vPrL/IL (L3). Micrographs at 20 × covered most of the cortical layers (I/II–III and V). For MBP analysis at approximately + 0.48 mm from bregma (sensory cortex), in each hemisphere a picture was taken (20 ×) at a fixed distance from the cingulum, having the external capsule horizontally aligned and moving a fixed distance from the external capsule into the sensory cortex (see Fig. 4B for details). Micrographs at 20 × were taken in layer V of the cortex as was assessed by aligning CTIP staining with MBP (Fig. 4C). Densitometry for MBP + area was analyzed by an experimenter blinded to experimental groups using Image J software 1.47 using background subtraction and automated thresholding. Settings of the first analyzed micrograph were copied to all micrographs to ensure similar thresholds between all animals. Values of the photographs were averaged between both hemispheres (per layer) per animal. MBP + area in WMI animals is depicted relatively to MBP + area in control animals which is put at 100%.

### Behavioural assessment 5-choice serial reaction time task

The 5CSRTT (Baarendse and Vanderschuren 2012; Bari et al. 2008; Carli et al. 1983) took place in operant conditioning chambers (Med Associates, St. Albans, VT, USA) with an array of five response holes located on one wall and a food tray, through which 45 mg sucrose pellets (TestDiet, St. Louis, MO) were delivered, on the opposite wall, 2 cm above a metal bar floor. A stimulus light was located within each hole, and a horizontal infrared beam detected nose-poke responses into these apertures. The entire chamber could be illuminated by a light at the top of the chamber.

Rats were trained to detect and respond to a brief visual stimulus presented randomly in one of the five nose poke units to obtain a food reward. A trial started with an inter-trial interval (ITI) of 5 s, followed by 1 s illumination of one of the five apertures and 2 s limited hold. Following a nose poke into the illuminated aperture, i.e. a correct response, animals were rewarded with the delivery of one sucrose pellet into the food magazine. During the training session, stimulus duration was set at 32 s, which was gradually decreased over sessions to 1 s until animals reached stable baseline performance (accuracy > 80% correct choice; < 20 omissions). Each daily session consisted of 100 discrete trials or 30 min, whichever occurred first. A nose-poke response into a non-illuminated aperture, i.e. an incorrect response, as well as a failure to respond within 5 s after the onset of the stimulus, i.e. an omission, resulted in no food delivery and a time-out period with the house light extinguished for 5 s. Nose pokes made during the ITI, i.e. before the onset of the stimulus (premature responses), were recorded as a measure of impulsivity, and resulted in a 5-s time-out and no food reward. Perseverative responses, i.e. repeated responding during the presentation of the stimulus, were measured but did not have any programmed consequences. The following behavioural measures were recorded: (1) accuracy, i.e. percentage of correct responses [(number correct responses)/(correct + incorrect responses) × 100]; (2) premature responses, i.e. number of responses into any of the holes during the ITI preceding stimulus presentation; (3) omissions, i.e. the total number of omitted trials during a session; (4) perseverative responses after correct choice; (5) latency of correct responses, i.e. the mean time between stimulus onset and nose poke in the illuminated unit; (6) latency to reward, i.e. the mean time between a correct nose poke and the collection of the sucrose pellet (Baarendse and Vanderschuren 2012).

Several parameters of the 5CSRTT were manipulated in order to determine impulse control, attention and cognitive flexibility of the animals. To assess *impulse control*, a long (7 s) ITI, a long variable ITI (between 5 and 13 s) and a mean variable ITI (between 3 and 7 s) were used; these manipulations typically lead to an increase in premature responses (Baarendse and Vanderschuren 2012; Dalley et al. 2007; Irimia et al. 2015). To study *sustained attention*, we used a short ITI of 2 s, a short variable ITI (between 1 and 5 s), a variable short stimulus duration (alternating every 20 trials between 1.2 and 0.5 s) or we introduced a distractor into the testing chamber in the form of a fixed or loose wooden block (fixed: a hurdle protruding 1 cm into the test chamber dividing the chamber in the middle; loose: 8 cm (l) × 3 cm (w) × 3 cm (h) placed in the middle and can be moved around, this is also used a homecage enrichment); these manipulations typically reduce accuracy and increase errors of omission. Finally, to determine *cognitive flexibility*, perseverative responses were assessed in all manipulations of the 5CSRTT. Impairments of cognitive flexibility are typically shown as increases in perseverative responses (Dalley et al. 2002). To evaluate the motivation to work for sucrose, animals were allowed ad libitum access to chow for at least 48 h before assessing their performance in the 5CSRTT. This manipulation usually results in an increase in errors of omission (Bari et al. 2008; Grottick and Higgins 2002; Harrison et al. 1997). Between every test, animals were subjected to daily training sessions as previously described until a stable baseline performance was reached (accuracy: > 80%; omissions: < 20). Tests measuring the same construct (e.g. impulse control) were not tested consecutively. The exact order of test manipulations was as follows: long ITI, short stimulus duration, short ITI, fixed block, short variable ITI, short variable stimulus duration, mean variable ITI, loose block, long variable stimulus duration, ad libitum food access. Data are presented as baseline performance preceding the test vs. manipulation.

#### Probabilistic reversal learning

After testing in the 5CSRTT had finished, the animals were tested in the probabilistic reversal learning task (Bari et al. 2010; Izquierdo and Jentsch 2012) which took place in standard operant conditioning chambers equipped with two retractable levers and a food magazine in between the levers.

After a 5-day period that consisted of habituation and training, animals were subjected to the reversal learning task for 5 consecutive daily sessions of 120 trials each. In this task, both levers were presented, one of which was randomly assigned as the initially high probability lever, while the other lever was assigned the initially low probability lever. Whereas a press on the high probability lever resulted in presentation of a food reward with 80% probability, a press on the low probability lever resulted in food reward with a 20% probability. Thus, adopting a strategy to continuously press the high probability lever would yield the maximum number of rewards to the animal. After 40 trials, the reward contingencies switched: the initially high probability lever became the low probability lever (20% probability of reward) and the initially low probability lever became the high probability lever (80% probability of reward). After another 40 trials, the reward scheme switched back to the initially high probability lever becoming high probability again and the initially low probability lever becoming low probability again. The ability of animals to change their strategy in response to a switch in the high vs. low probability levers is considered a measure of cognitive flexibility. The following parameters were assessed: the number of trials that animals pressed the high probability lever, as well as win-stay- (pressing the same lever after obtaining a reward) and lose-shift- (switching to the other lever when a lever press does not result in reward) behaviour, reaction time (the time it takes the individual to respond on a lever) and the percentage of pellets earned compared to the maximum obtainable sucrose pellets.

#### Statistics

Performance in the 5CSRTT was analyzed using a repeated-measures ANOVA, with manipulation (baseline vs. test) as within-subjects factor and treatment (control vs. WMI) and sex (male vs. female) as between-subjects factors. In case a significant effect or interaction with sex was observed, both sexes were further analyzed separately. In case no significant effect of sex was observed, both sexes were pooled to be analyzed by repeated-measures ANOVA to determine the effects of the manipulation (within-subjects factor: baseline vs. test) and treatment (between-subjects factor: control vs. WMI). Post hoc tests were performed when appropriate and *p*-values of *t*-tests were adjusted with a Bonferroni correction.

Parameters in the reversal leaning task were analyzed with a repeated-measures ANOVA. For the number of lever presses on the high probability lever, within-subject factors were test day (day 1–5) and session (1: trial 0–40, 2: trial 41–80 and 3: trial 81–120). Between-subject factors were treatment (control vs. WMI) and sex (male vs. female). For the other parameters in the reversal learning task, the within-subject factor was test day (day 1–5) and the between-subject factors were treatment (control vs. WMI) and sex (male vs. female).

##### Computational modelling

The trial-by-trial data of the probabilistic reversal learning task was also subjected to computational modelling (Verharen et al. 2019) with parameter estimation from Verharen et al. (2018). In brief, a modified version of the Rescorla-Wagner Q learning model was fit to the data of the individual sessions, explaining the behaviour of the rats on the basis of four parameters: reward learning rate α + (sensitivity to rewarding feedback), punishment learning rate α- (sensitivity to punishment or absence of reward), stickiness parameter π (a measure for perseveration, i.e. a preference for the last chosen lever, independent of outcome) and explore/exploit parameter β (measure of the extent to which animals consistently chose the highest valued option). Best-fit parameters were estimated using maximum likelihood estimation with the function *fmincon* in Matlab (Version R2018b; The MathWorks Inc. USA), and were compared between groups using traditional statistical tests. In case of significant interaction effects, univariate ANOVAs and paired and unpaired *t*-tests with Bonferroni corrections were performed when appropriate.

Immunohistochemical data (percentage MBP area) were analyzed using independent samples *t*-tests and in case of unequal variances, Welch’s corrections were applied. All data are presented as mean + SEM.

## Results

### Behavioural assessments

#### 5CSRTT

Due to the large amount of parameters assessed in the different manipulations of the 5CSRTT, only the most outstanding effects of WMI and sex are described in the results (see Table S1–S11 for the complete results). In Tables 1, 2 and 3, an overview is presented of significant results.

**Table 1:**
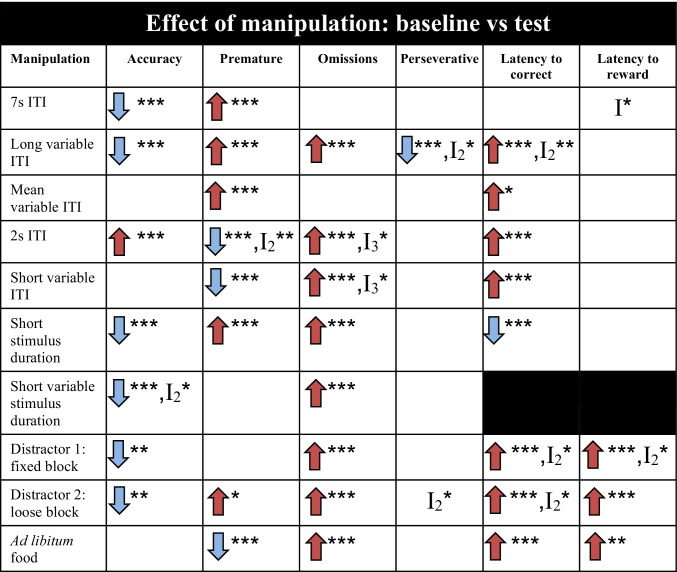
Effect of manipulation on test performance in the 5CSRTT compared to baseline. Baseline parameters for each manipulation can be found in supplementary tables S1–S11

*I* interaction effect, *I*_*2*_ two-way interaction effect, *I*_*3*_ three-way interaction effect, *ITI* inter-trial-interval, blue arrow: decrease in parameter compared to baseline, red arrow: increase in parameter compared to baseline. Significances are indicated with *p ≤ 0.05, **p < 0.01, ***p < 0.001

**Table 2:**
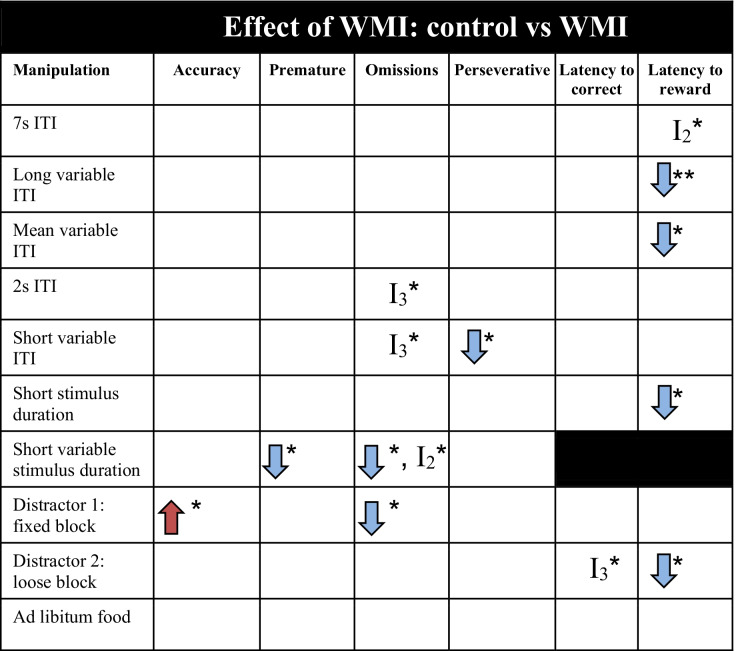
Effect of white matter injury (WMI) compared to control animals on test performance in the 5CSRTT. Baseline parameters for each manipulation can be found in supplementary tables S1–S11

*I* interaction effect, *I*_*2*_ two-way interaction effect, *I*_*3*_ three-way interaction effect, *ITI* inter-trial-interval, blue arrow: decrease in parameter compared to control animals, red arrow: increase in parameter compared to control animals. Significances are indicated with *p ≤ 0.05, **p < 0.01, ***p < 0.001

**Table 3:**
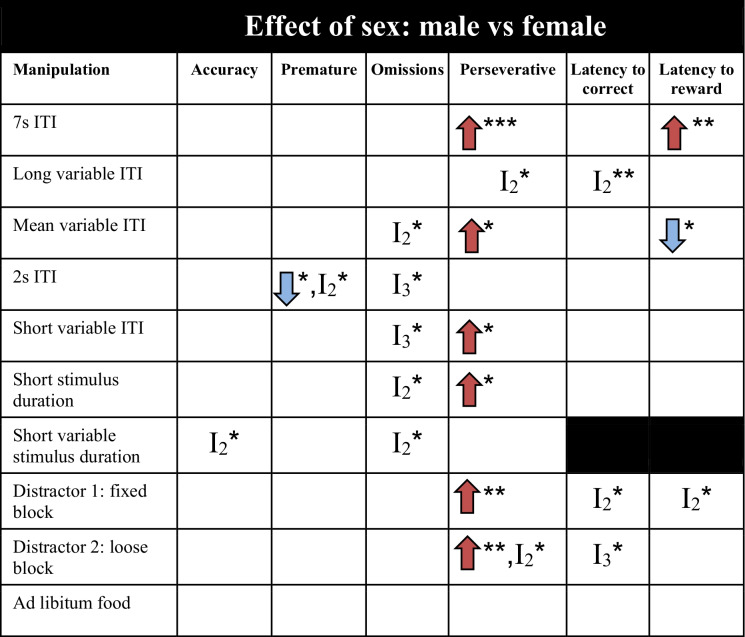
Effect of sex on test performance in the 5CSRTT. Baseline parameters for each manipulation can be found in supplementary tables S1–S11

*I* interaction effect, *I*_*2*_ two-way interaction effect, *I*_*3*_ three-way interaction effect, *ITI* inter-trial-interval, blue arrow: decrease in parameter compared to male animals, red arrow: increase in parameter compared to male animals. Significances are indicated with *p ≤ 0.05, **p < 0.01, ***p < 0.001

### Training and motivation

Animals were trained until they responded stably (criteria: accuracy > 80% and omissions < 20) in the task. Stability for both criteria was reached in 49 training days. Performance was comparable for control and WMI animals as well as for male and female rats (see Table S1). Together, these results indicate that control and WMI animals of both sexes do not differ in acquisition of the 5CSRTT and thus have a similar starting point. No differences in accuracy or the latency to respond and obtain the reward were found between WMI and control animals and males and females after the ad libitum food test, indicating similar motivation to perform the task in all groups (see Table S2).

### Most prominent effects: omissions, interactions of treatment and sex

The most prominent effects on 5-choice task performance compared to baseline were found on the amount of omissions made when the ITI was short (Table S3), short and variable (Table S4) or varied around the mean (both longer and shorter; Table S5).

**Fig. 1:**
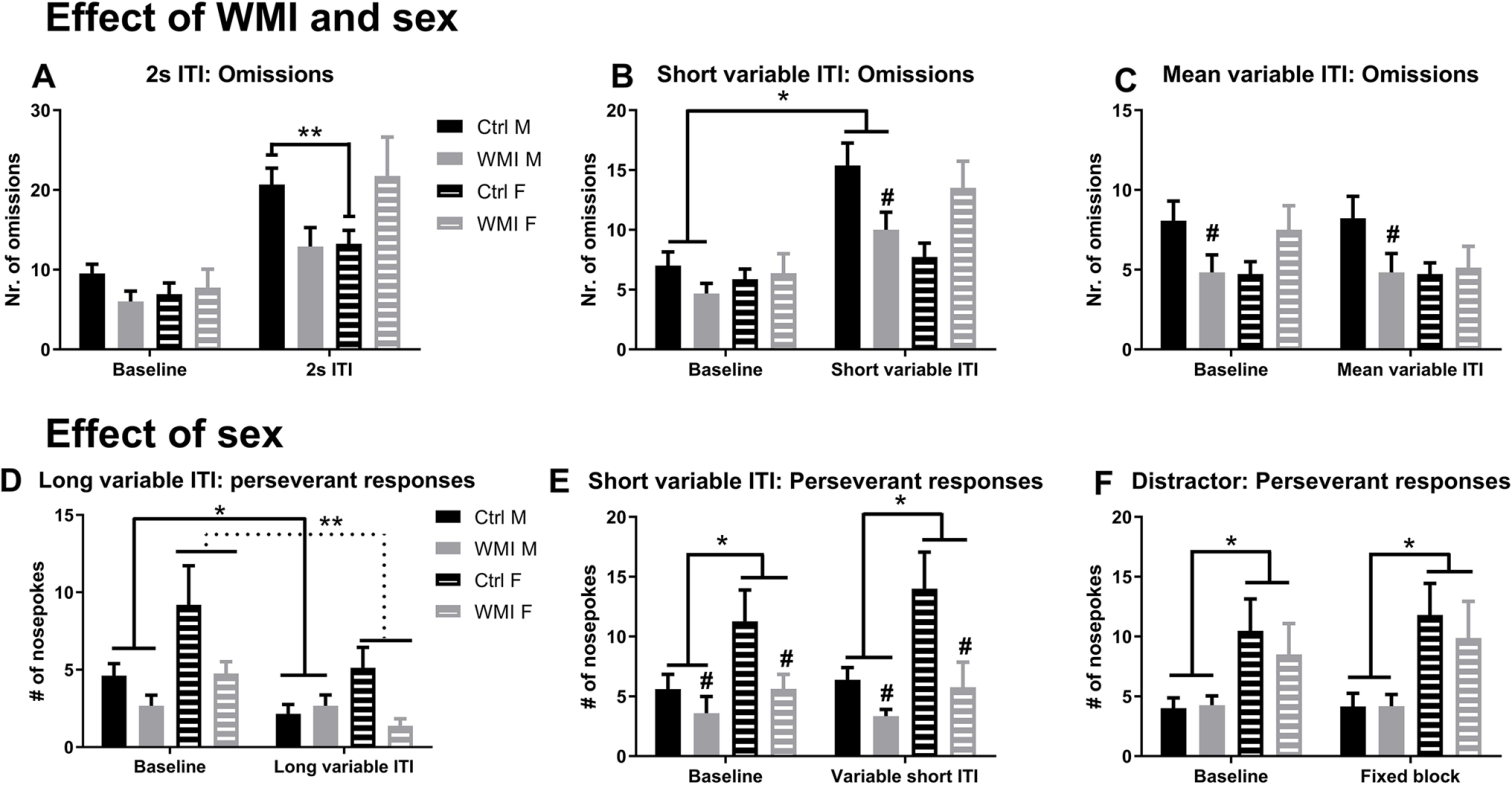
Most striking effects of WMI (white matter injury) and sex on performance in the 5-choice serial reaction time task. Under short (**A**) and variable (**B**, **C**) inter-trial interval (ITI) manipulations of the task, WMI males (solid grey bar; WMI M) omit less whereas WMI females (striped grey bar, WMI F) omit equal or more trials, indicating that under high attentional load WMI males perform better whereas female WMI rats perform equal compared to same sex control animals (male control: solid black bar, Ctrl M; female control: solid grey bar, Ctrl F). A sex-specific effect was observed in the number of perseverative responses, an indicator cognitive inflexibility, i.e. the inability to alter behaviour in reaction to a changing situation or task. Females made more perseverative responses irrespective of task manipulations, since baseline and test performance did not differ (long (**D**) and short variable (**E**) ITI manipulations or the presence of a distractor (**F**)). Data is presented as mean + SEM. *p < 0.05, **p < 0.01, compared to the group connected with the line. #*p* < 0.05 compared to the same condition control group

Animals omitted more trials in the 2 s ITI and short variable ITI test compared to baseline (2 s ITI: Ftest(1,44) = 53.05, *p* < 0.001; short variable ITI: Ftest(1,44) = 44.15, *p* < 0.001, see Fig. 1A and B).

When the ITI was shortened to 2 s, WMI animals differed from control animals in the amount of omissions (Ftest*treatment*sex(1,44) = 5.10, *p* = 0.03; Ftreatment*sex(1,44) = 9.72, *p* < 0.001, see Fig. 1A). Under baseline conditions, WMI and control animals of both sexes did not differ in their omissions (baseline: Ftreatment*sex(1,44) = 2.06, *p* = 0.16). However, a short ITI resulted in a different response in males and females (test: Ftreatment*sex(1,44) = 9.97, *p* = 0.003), male control animals omitted more trials compared to female control animals (*t*(26) = 2.85, *p* = 0.008) whereas male and female WMI animals did not significantly differ in their omitted trials (*t*(18) =  − 1.80, *p* = 0.09; see Fig. 1A). In addition, both male and female WMI animals did not differ in their omissions to their controls (during the short ITI: males WMI vs. control: *t*(23) = 2.49, *p* = 0.02, females WMI vs. controls (*t*(21) =  − 2.03, *p* = 0.06, Bonferroni-corrected *p*-value: *p* < 0.025).

When the time between two trials was short and variable (see Fig. 1B), both males and females made more omissions when the ITI was short and variable compared to baseline (Ftest*treatment*sex(1,44):5.91, *p* = 0.02; Ftreatment*sex(1,44) = 9.52, *p* = 0.004; males: Ftest(1,23) = 34.11, *p* < 0.001; females: Ftest(1,21) = 13.25, *p* = 0.002). In addition, male WMI rats omitted less trials than control males in test conditions (Ftreatment(1,23) = 5.69, *p* = 0.03; Ftest*treatment(1,23) = 1.69, *p* = 0.21), whereas this was not observed in females (Ftest*treatment(1,21) = 4.53, *p* = 0.05; Ftreatment(1,21) = 4.04, *p* = 0.06).

Omissions made in the test where the ITI varied around the mean did not differ from baseline (Ftest(1,44) = 1.31, *p* = 0.26, Fig. 1C). However, an interaction effect of treatment and sex was found in the amount of omitted trials (Ftreatment*sex(1,44) = 5.48, *p* = 0.02, see Fig. 1C). Male WMI rats omitted less trials compared to control males in both baseline and test conditions (Ftreatment(1,23) = 4.37, *p* = 0.048), whereas WMI and control females omitted trials equally both under baseline and test-conditions (Ftest(1,21) = 3.55, *p* = 0.07; Ftest*treatment(1,21) = 3.55, *p* = 0.07; see Fig. 1C and Table S5).

### Sex differences: perseverative responses and accuracy

In general, female rats made more perseverative responses compared to males under baseline conditions irrespective of experimental condition. The long variable ITI manipulation and the presence of a loose distractor resulted in a different behavioural pattern between the sexes (long variable ITI: Ftest*sex(1,44) = 4.63, *p* = 0.04; loose distractor: Ftest*sex(1,44) = 6.00, *p* = 0.02) whereas in the short variable ITI condition (short variable ITI: Fsex(1,44) = 5.39, *p* = 0.03), the WMI males made less perseverative responses.

In the long variable ITI condition (for full results and statistics, see table S7), when the sexes were analyzed separately, both males and females made less perseverative responses (males: Ftest(1,23) = 7.55, *p* = 0.01; females Ftest(1,21) = 10.94, *p* = 0.003, see Fig. 1D) compared to baseline but no effect of treatment was found (males: Ftreatment(1,23) = 0.67, *p* = 0.42; females Ftreatment(1,21) = 2.54, *p* = 0.13). In addition, the males showed a difference in response due to treatment (Ftest*treatment) = 7.55, *p* = 0.01, Fig. 1D): under baseline conditions male WMI rats showed a trend towards making less perseverative responses compared to control males (males baseline: control vs. WMI *t*(24) = 3.46, *p* = 0.08; males test: control vs. WMI *t*(24) = 0.31, *p* = 0.58). Female rats did not significantly show this interaction effect (Ftest*treatment) = 0.09, *p* = 0.76).

In the short variable ITI test, no effect of test was found in the amount of perseverative responses (see Table S4 for full results and statistics). When analyzed separately per sex, male WMI rats made less perseverative responses compared to control rats (Ftreatment(1,23) = 4.69, *p* = 0.04). Although the same pattern is observed in females rats, this was not significant (Ftreatment(1,21) = 3.25, *p* = 0.09, see Fig. 1E).

When analyzed per sex, in the presence of a loose distractor in the cage (for full results and statistics, see table S11), females tend to increase their perseverative responses compared to baseline (females: Ftest(1,21) = 3.70, *p* = 0.07); males Ftest(1,23) = 2.14, *p* = 0.16; see Fig. 1F). No effect of treatment or an interaction of test and treatment were found in both sexes (see table S11).

In the variable short stimulus duration condition, males tended to be less accurate during the short stimulus duration trials (Fsex(1,44) = 3.74, *p* = 0.06) and the sexes reacted differently to the test conditions (Fsex*test(1,44) = 2.45, *p* = 0.048). Post hoc analysis per sex showed that male rats were less accurate during the short stimulus duration trials (Ftest(1,23) = 10.29, *p* < 0.001) whereas female rats were equally accurate during test and baseline trials (Ftest(1,21) = 1.80, *p* = 0.14, see table S9).

### Impulsivity: long 7 s ITI, long variable ITI and mean variable ITI

The ITI was increased to a fixed time of 7 s or a long variable time (between 5 and 13 s) or differed around the mean (between 3 and 7 s) in order to assess the animal’s ability to withhold a response (i.e. impulsivity) (for full results and statistics, see table S5-S6-S7).

The manipulations resulted in expected and previously reported effects, i.e. increased premature responses and decreased accuracy without an effect on perseverative responses, the latency to a correct response or the latency to obtain a reward. In the variable long ITI test, in addition to increased premature responses and a reduced accuracy, also more trials were omitted, less perseverative responses were made and the latency to make a correct response was increased, without the latency to obtain the reward being affected. When the ITI varied around the mean, premature responses increased and it took longer to make a correct response. The accuracy, omissions, perseverative responses and the latency to collect a reward did not change.

On average, treatment and sex did not affect performance. The only differences observed were in the long variable or mean variable ITI condition, where WMI animals were faster in collecting the reward, both under baseline as well as test conditions (long variable ITI: Ftreatment(1,44) = 10.61, *p* = 0.002; mean variable ITI: Ftreatment(1,44) = 4.74, *p* = 0.04). No other effects of WMI were found in the long- and mean-variable ITI manipulation (see table S5 and S7). Males and females showed a difference in the latency to make a correct response in the long variable ITI manipulation (Ftest*sex(1,44) = 4.63, *p* = 0.04). Under test conditions, males took longer to make a correct response whereas for females this was only a trend (males: Ftest(1,23) = 68.49, *p* < 0.001; females: Ftest(1,21) = 3.30, *p* = 0.08). Under baseline conditions, males and females performed similar (see Table S7).

### Sustained attention: 2 s ITI, short variable ITI, a fixed or variable short stimulus duration and distractors

To investigate attentional performance, animals were subjected to a fixed short ITI (2 s), a short variable ITI (between 1 and 5 s), a fixed and variable short stimulus duration (fixed: stimulus duration is 0.5 s; variable: stimulus duration is alternating every 20 trials between 1.2 and 0.5 s) and two distractors (distractor 1: a fixed hurdle in the middle of the box and distractor 2: a wooden block that can be moved around the cage).

Tasks that place a high demand on attention are reported to result in decreased accuracy, increased omissions and longer response latencies without affecting premature responding. In line with this expectation, manipulations that place a high demand on attention made animals less accurate (short stimulus duration: Ftest(1,44) = 102.86, *p* < 0.001; short variable stimulus duration: Ftest(1,44) = 8.17, *p* < 0.001; distractors: fixed block: Ftest(1,44) = 10.69, *p* = 0.002; loose block: Ftest(1,44) = 12.46, *p* < 0.001, see Tables S8-S9-S10-S11), with the exception of an increase in accuracy by shortening the ITI to 2 s (Ftest(1,44) = 34.67, *p* < 0.001) and no difference compared to baseline in the short variable ITI condition (see table S3 and S4 for full results and statistics).

In addition, the manipulations indeed resulted in more omitted trials (see Tables S8-S11). Furthermore, animals made either less (2 s ITI and short variable ITI, Table S3-S4), more (short stimulus duration and loose block distractor, Table S8 and S11) or did not differ (variable short stimulus duration or the fixed block distractor, Table S9-S10) in the amount of premature responses.

The latency to a correct response was in most test conditions longer compared to baseline conditions in all animals (2 s ITI: Ftest(1,44) = 100.09, *p* < 0.001; short variable ITI: Ftest(1,44) = 34.34, *p* < 0.001, and both the distractors: fixed block: Ftest(1,44) = 20.37, *p* < 0.001, loose block: Ftest(1,44) = 19.17, *p* < 0.001, Table S3-S4 and S10-S11). A fixed but not variable short stimulus duration decreased the time it took the animals to make a correct response (short stimulus duration (Ftest(1,44) = 66.66, *p* < 0.001, see Table S8-S9).

The latency to obtain the reward was longer when a distractor was present (fixed block: Ftest(1,44) = 16.31, *p* < 0.001, loose block: Ftest(1,44) = 21.82, *p* < 0.001, see Table S10-S11), but did not differ with shorter (and variable) ITIs and stimulus durations (see Table S6-S9).

With a few exceptions, no effects were found of WMI and sex on performance. A fixed distractor made control animals less accurate compared to WMI animals (Ftreatment(1,44) = 5.25, *p* = 0.03, see Table S10). In the variable stimulus duration test, animals with WMI made less premature responses compared to control animals (Ftreatment(1,44) = 3.99, *p* = 0.05, Table S9). Females in the 2 s ITI test made less premature responses compared to males, especially in baseline conditions since there were hardly any premature responses in the test condition (Fsex(1,44) = 6.74, *p* = 0.01; Ftest*sex(1,44) = 6.41, *p* = 0.02; post hoc: baseline male vs. female: F(1,46) = 7.11, *p* = 0.01; test male vs. female F(1,46) = 1.51, *p* = 0.23). No effects on premature responses were found for the short variable ITI manipulation (see Table S3-S4).

Both distractors differentially affected the latency to a correct response in male and female rats (fixed block: Fsex*test(1,44) = 4.42, *p* = 0.04; loose block: Fsex*test(1,44) = 6.21, *p* = 0.02; Fsex*test*treatment(1,44) = 4.77, *p* = 0.03). Post hoc testing per sex showed that in the fixed and loose block manipulations, males take longer to make a correct choice (fixed block: Ftest(1,23) = 21.22, *p* < 0.001; loose block: Ftest(1,23) = 22.52, *p* < 0.001), whereas females did not differ or tended to be faster between baseline and test conditions in their response time (fixed block: Ftest(1,21) = 3.12, *p* = 0.09, see Table S10; loose block: Ftreatment(1,21) = 3.55, *p* = 0.07 see Table S11). The fixed block distractor differentially affected the latency to a reward in males and female rats (fixed block: Fsex*test(1,44) = 4.42, *p* = 0.04). Post hoc testing per sex showed that male rats took longer to collect the reward when the distractor was present but female rats did not (males: Ftest(1,23) = 67.26, *p* < 0.001; females: Ftest(1,21) = 0.97, *p* = 0.34, see Table S10).

### Probabilistic reversal learning test

To assess cognitive flexibility in control and WMI animals, a probabilistic reversal learning test was used. Due to the large amount of parameters assessed, only the most outstanding effects of WMI and sex are described in the results (see Table S12-S20 for the complete results).

**Fig. 2:**
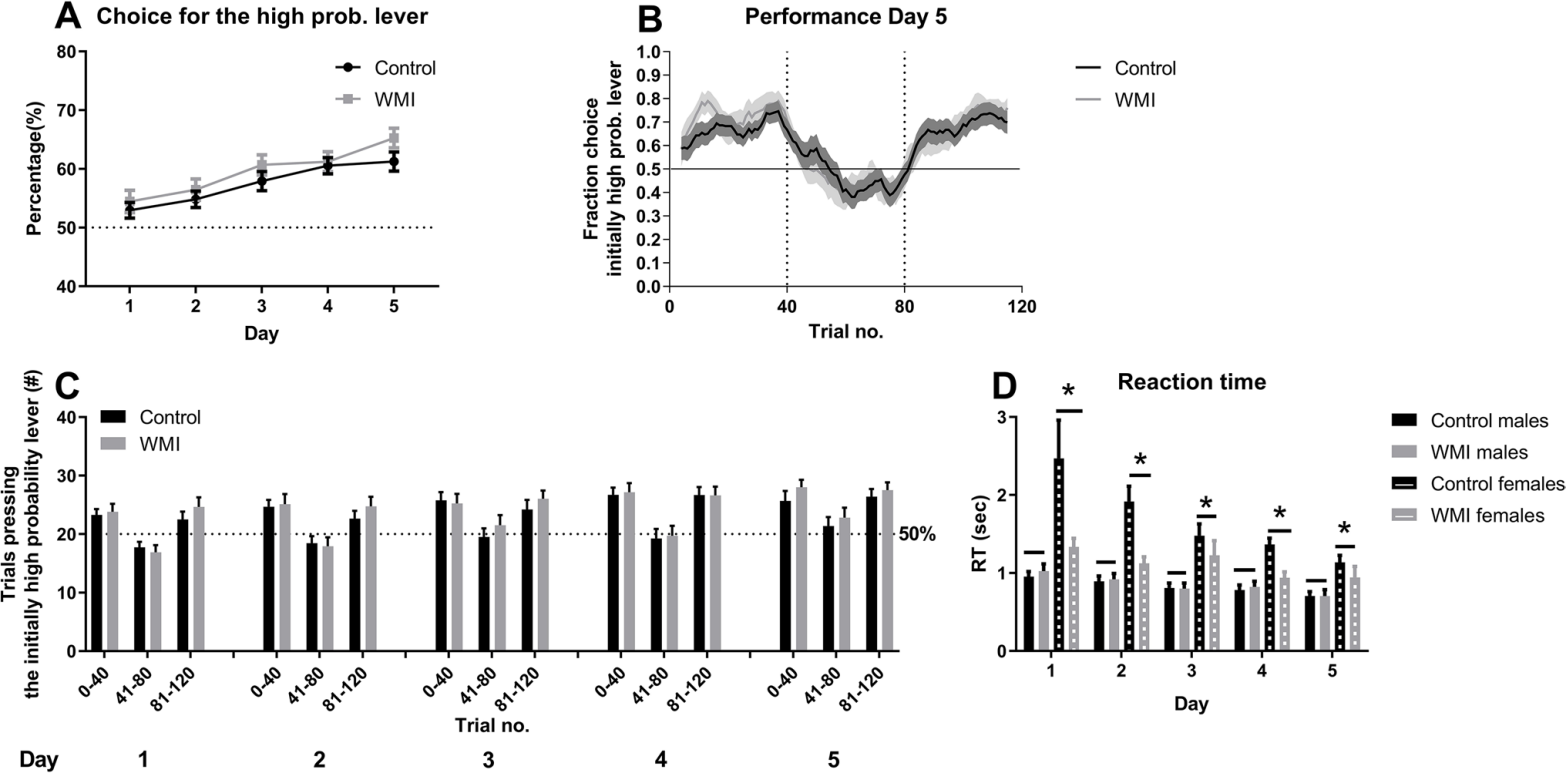
Performance of control (black bars) and WMI animals (grey bars) in the probabilistic reversal learning test. (**A**) Over the course of 5 sessions that animals performed the probabilistic reversal learning task animals showed a gradual increase in performance, as indicated by the proportion of trials that animals pressed the lever with high probability of a reward. No difference was observed between control and WMI animals (data for males and females was pooled because of a lack of effect of sex). (**B**) The fraction of choice for the initially high probability lever, based on a shifting window of 8 consecutive trials (i.e. for each time point the fraction of choice for the high probability lever of 4 trials before and 4 trials after that time point was calculated). Both control and WMI animals deployed a successful strategy during the final probabilistic reversal learning task: animals pressed the initially high probability lever more frequently during trials 0–40, successfully adapted their strategy to pressing the initially low probability lever during trials 41–80 and changed back to the initially high probability lever during trials 81–120. No differences were observed between control and WMI animals (data for males and females was pooled because of a lack of effect of sex). (**C**) Performance on the 5 test days. The number of trials during which animals pressed the high probability (80%) lever are shown for trials 0–40, 41–80 and 81–120 separately. The expected number of lever presses in the case of no preference for a specific lever (i.e. 20; 50%) is indicated by the dotted line (data for males and females was pooled because of a lack of effect of sex). (**D**) Reaction time per day per treatment (control vs. WMI) and per sex (males, solid bars; females, striped bars). Males are always faster than females. Reaction time is initially longer for female rats and decreases over days. Female WMI rats were faster compared to control females, whereas males were equally fast in both treatment conditions. Data is presented as mean + SEM. *p < 0.05

Over the course of 5 consecutive 120-trial sessions, the animals improved in performance in this task, they increased the proportion of trials that animals pressed the high probability lever (Fday(4,176) = 13.97, *p* < 0.001) (Fig. 2A). WMI and sex did not affect performance of the animals (Ftreatment(1,44) = 2.58, *p* = 0.12; Fsex(1,44) = 1.72, *p* = 0.20)(Fig. 2A and Table S12).

Animals of both groups successfully developed a strategy by pressing the initially high probability lever > 50% of the trials, switched to the other lever (initially low probability but now high probability of receiving a reward) when the probabilities changed and switching back when the contingencies changed back again (Fsession(2,88) = 48.00, *p* < 0.001; Fig. 2B/C, detailed statistics on the performance of animals during trials 1–40, 41–80 and 81–120 during sessions 1–5 can be found in table S12).

Reaction time in the task improved over days (Fdays(4,172) = 8.62, *p* < 0.001) and male rats where always faster than females (Fdays*sex(4,172) = 2.41, *p* = 0.05, see Fig. 2D). Reaction time differed between sexes and treatment (Fsex(1,43) = 16.45, *p* < 0.001; Ftreatment(1,43) = 3.87, *p* = 0.03; Fsex*treatment(1,43) = 4.62, *p* = 0.02). Post hoc testing revealed that female WMI rats were faster compared to female control rats (*t*female(20) = 2,96, *p* = 0.008) whereas males were equally fast in both treatment conditions (*t*male(23) =  − 0.30, *p* = 0.72). Both in control and WMI conditions, male rats were faster compared to female rats (*t*control(26) =  − 5.05, *p* < 0.001; *t*wmi(17) =  − 2.18, *p* = 0.04; Table S13).

Win-stay performance increased over days (Fdays(4,172) = 27.31, *p* < 0.001), as well as the number of pellets animals earned (Fdays(4,172) = 5.55, *p* < 0.001). No other effects were found for these parameters (see tables S14: win-stay and S15: pellets won). Lose-shift behaviour differed for the WMI and control animals over days (Fday*treatment(4,172) = 3.21, *p* = 0.01). Post hoc analysis showed that only on day 3, WMI animals shifted away more from the lever they were pressing after a loss compared to control animals (*t*day3(45) =  − 2.00, *p* = 0.05), and on the other days, no differences were found (See table S16).

Finally, we fitted the trial-by-trial data to a Q learning model (Verharen et al. 2019) to assess the effects of WMI on the component processes underlying probabilistic reversal learning performance. A comparison of the best-fit model parameters revealed that animals demonstrated an increase in reward learning parameter α + over days (Fdays(4,120) = 4.00, *p* = 0.004), but there was no difference between groups, indicating a stronger impact of positive (rewarding) feedback on behaviour in later sessions in all animals. Post hoc comparisons showed that there was a reduced learning rate on day 1 compared to day 5 (*t*_1vs5_(45) =  − 0.30, *p* = 0.01); i.e. all animals adapted their behaviour more strongly in response to positive feedback on day 5 as compared to day 1. No other effects on reward learning were found (Table S17). Learning from negative feedback (punishment learning rate α-) did not differ over days or between sexes or groups (Table S18). In addition, perseveration, measured as stickiness parameter π in the computational model (indicating a preference for the lastly chosen lever, independent of outcome), was not different over days or between sexes and groups (Table S19) and was close to 0. This indicates that the side chosen in the last trial did not affect the choice of the animal in the upcoming trial much, and thus, choices were based mostly on the value representation of the two options rather than perseverative behaviour on the same lever. The exploit/explore parameter (β), measuring the extent to which animals consistently chose the highest valued option, did not differ either over days or between sexes or groups (Table S20). Together, these results indicate that WMI and control animals behaved similarly in the task.

### Absence of myelin deficits in PFC subregions

**Fig. 3:**
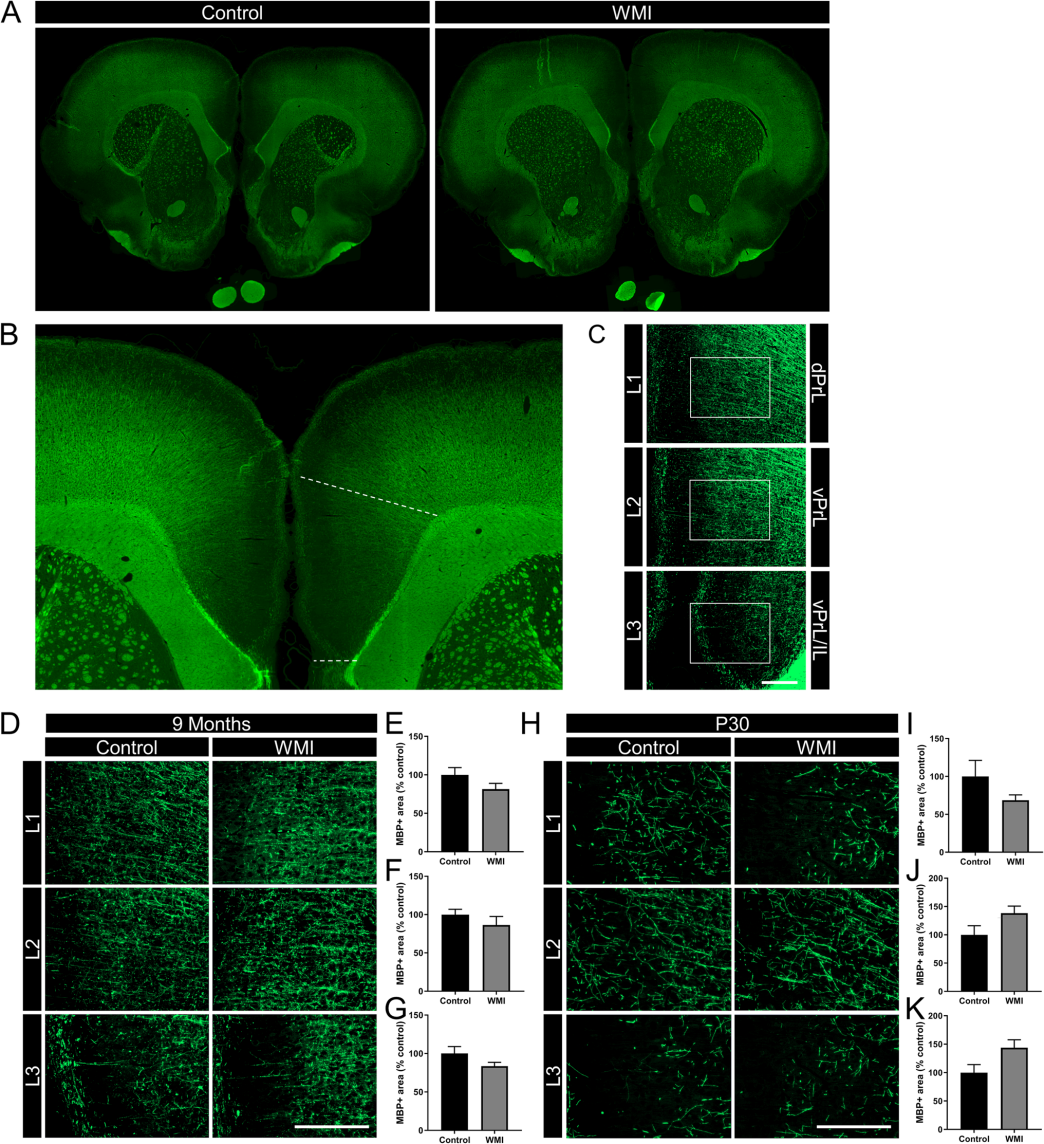
The double-hit model of fetal inflammation and postnatal hypoxia does not cause myelination failure in the prefrontal cortex. (**A**) Full slice scans of MBP staining in a representative control and WMI animal at 9 months of age, at approximately + 2.20– + 2.35 mm from bregma. (**B**) Zoom-in of full slice scan depicted in (**A**) (control animal) showing the location within the mPFC in which photographs were made (between dashed lines). (**C**) 10 × magnification micrographs taken within the outlined area in (**B**) covering the dorsal prelimbic (dPrL; L1), ventral prelimbic (vPrL; L2) and border between vPrL and infralimbic (vPrL/IL; L3) cortices. Squares indicate the location of 20 × micrographs. (**D**) Representative images (20 ×) of MBP staining in control and WMI animals at 9 months of age in the three layers. (**E**–**G**) Quantification of MBP + area in control versus WMI animals at 9 months in layer 1 (**E**), layer 2 (**F**) or layer 3 (**G**). (**H**) Representative images (20 ×) of MBP staining in control and WMI animals at P30 in the three layers. (**I**–**K**) Quantification of MBP + area in control versus WMI animals at P30 in layer 1 (**I**), layer 2 (**J**) or layer 3 (**K**). MBP + area in WMI animals is depicted as percentage of MBP + area in control animals which were put at 100%. Control animals *n* = 8, WMI animals *n* = 7 at 9 months of age and control animals *n* = 6, WMI animals *n* = 6 at P30. Scale bars in (**C**), (**D**), (**H**) represent 200 µm. Data in bar graphs is presented as mean + SEM

The prefrontal cortex is involved in cognitive functioning such as working memory, decision-making and inhibitory response control and attentional set-shifting (Dalley et al. 2004). Therefore, we explored possible myelination deficits in 3 subregions of the medial prefrontal cortex (mPFC) (dorsal prelimbic (dPrL), ventral prelimbic (vPrL) and the border of vPrL and infralimbic (IL) subregions) (Fig. 3). Figure 3A–C shows full slice scans, zoom-in and insets indicating the locations of the micrographs in these subregions of the mPFC. No differences were observed in myelination in the PFC subregions between control and WMI animals at the age of 9 months, i.e. after testing in the 5CSRTT (L1 prelimbic: *t*(13) = 1.06, *p* = 0.31; L2 infralimbic: *t*(13) = 1.53, *p* = 0.15; L3 dorsal penduncular: *t*(13) = 1.51, *p* = 0.16, Fig. 3D–G).

To rule out a possible positive effect of extensive training on myelination, a group of animals was subjected to the double-hit model and sacrificed at P30, the time point we previously observed myelination deficits in the sensory cortex. The data in Fig. 3H–K show that no significant differences in MBP + area were observed in WMI animals when compared to control animals in any of the 3 areas in the prefrontal cortex at P30 (L1 prelimbic: *t*(10) = 1.87, *p* = 0.10; L2 infralimbic: *t*(10) = 2.21, *p* = 0.05; L3 dorsal penduncular: *t*(10) = 1.41, *p* = 0.19). If anything, the WMI group showed a trend towards more myelination in the infralimbic cortex at this time point.

**Fig. 4:**
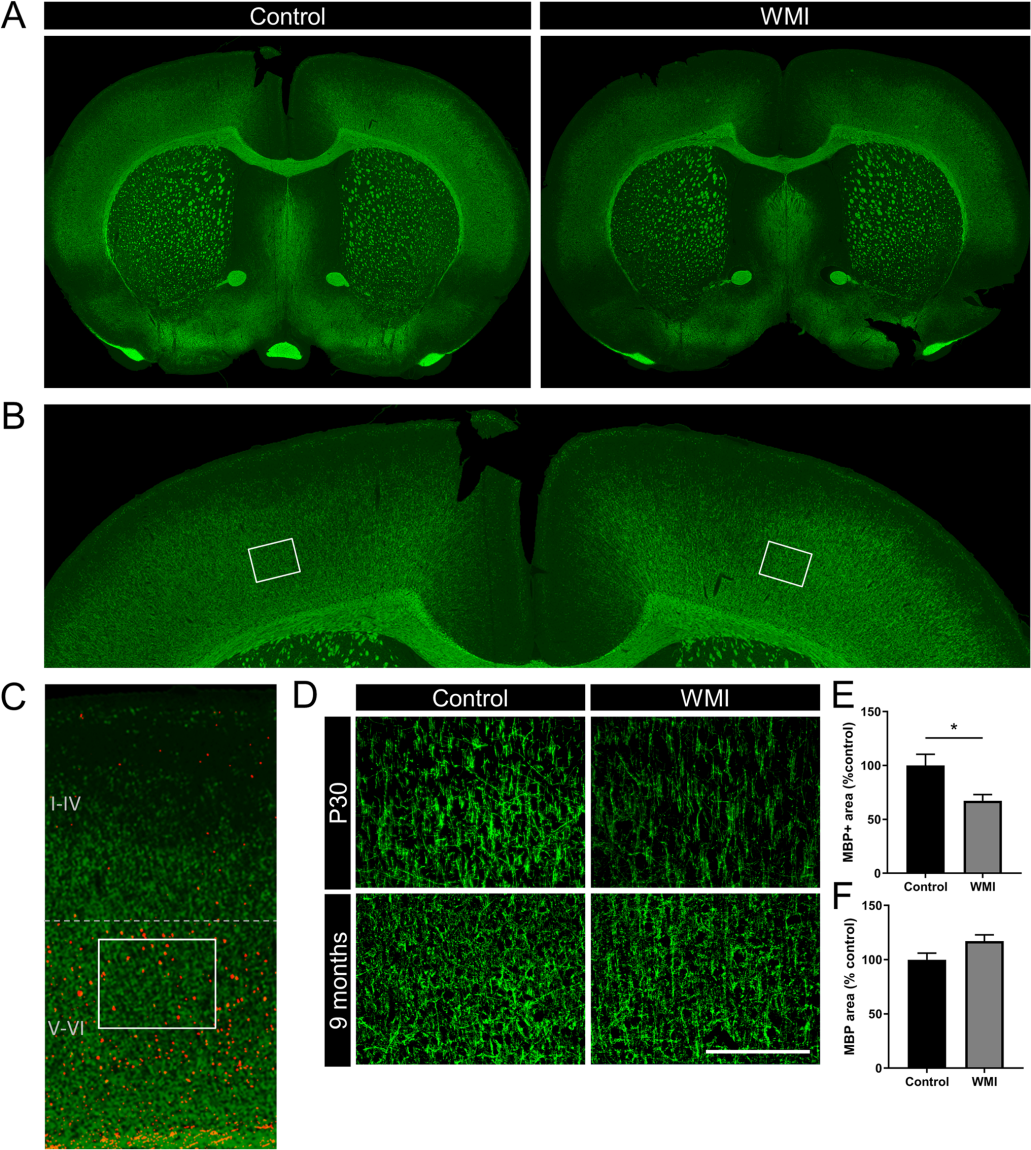
The double-hit model of fetal inflammation and postnatal hypoxia causes myelin deficits in the sensory cortex that restore over time. (**A**) Full slice scans of MBP staining in a representative control and WMI animal at P30, at approximately + 0.48 mm from bregma. (**B**) Zoom-in of full slice scan depicted in (**A**) (control animal) showing the location (squares) of the 20 × micrographs in both hemispheres. (**C**) CTIP staining (red) and MBP staining (green) were exactly aligned in a P30 brain to show localization of the 20 × micrographs within the cortical layers. The localization is in cortex layer V (upper part of CTIP + layers). (**D**) Representative images (20 ×) of MBP staining in control and WMI animals at P30 (upper) and 9 months of age (lower). (E + F) Quantification of MBP + area in control versus WMI animals at P30 (**E**) or 9 months (**F**). MBP + area in WMI animals is depicted as percentage of MBP + area in control animals which were put at 100%. Control animals *n* = 6, WMI animals *n* = 6 at P30 and control animals *n* = 8, WMI animals *n* = 7 at 9 months of age. Scale bar in (**D**) represents 200 µm. Data in bar graphs is presented as mean + SEM. *p < 0.05 control vs. WMI

To verify that our double-hit model of WMI, using fetal inflammation plus postnatal hypoxia, resulted in myelination deficits in the sensory cortex as described earlier (van Tilborg et al. 2018a, b), we checked myelination at P30 in the sensory cortex (Fig. 4). Figure 4A–C shows full slice scans, zoom-in and inset indicating the locations of the micrographs in layer V of the sensory cortex. Figure 4D–E confirms that WMI animals have ~ 30% loss of cortical MBP + area compared to control rats at P30 in this brain area (*t*(10) = 1.06, *p* = 0.02). Next, we assessed whether the myelination deficits observed at P30 in the sensory cortex were still present in this area at 9 months of age. At this later time point, we did not observe differences in myelination between control and WMI rats anymore in the sensory cortex (*t*(13) = 2.01, *p* = 0.07; Fig. 4D/F), indicating a transient, delayed deficit in myelination after fetal inflammation plus postnatal hypoxia which restored over time.

## Discussion

In the present study, we investigated the long-term consequences of WMI induced early in life by combined fetal inflammation and early postnatal hypoxia on executive functions in adulthood, in both male and female rats. In particular impulsivity, attention and cognitive flexibility in the 5CSRTT and reversal learning task were assessed, tasks that require a well-functioning prefrontal cortex. We observed that the amount of omitted trials was differently affected by WMI and sex when the task demanded a high attentional load, such as a short (variable) ITI or stimulus duration. In addition, males and females had a baseline difference in perseverative responses, i.e. females made more perseverative responses irrespective of task manipulation. In addition, whereas control females omit less trials in attention demanding tasks, WMI females have difficulties performing correctly in tasks of high attentional load. This pattern is reversed for males. In addition, we found little effect of WMI and sex on other parameters of the 5CSRTT; the manipulations of the 5CSRTT resulted in behavioural patterns previously observed in the literature. In the probabilistic reversal learning task, no effect of WMI was found. Males did perform faster in this task compared to females. The lack of effects observed in the cognitive tasks was supported by the myelination data of subregions of the PFC, which revealed no myelination deficits both at P30 and at 9 months of age. We did find a myelination deficit in the sensory cortex at P30, replicating the earlier findings in our double-hit model of WMI. Moreover, the deficits in myelination seem transient since this deficit in the sensory cortex was absent at 9 months of age.

### Effect of WMI on impulsivity, attention and cognitive flexibility

WMI animals did not show signs of increased impulsivity, as indicated by their ability to withhold a response during the long and variable long ITI manipulations of the task.

Attention was not impaired in rats exposed to fetal inflammation and postnatal inflammation, as WMI animals were as accurate as control rats in all tests. Interestingly, in baseline conditions, control males omitted more trials compared to male WMI rats whereas the amount of omitted trials in females was equal. In the short (variable) ITI and stimulus duration manipulations, male WMI rats omitted less trials whereas female WMI rats omitted equal amounts of trials compared to sex-specific controls. In addition, male control animals omitted more trials compared to female control animals whereas male and female WMI animals did not differ in their omitted trials. These results indicate that WMI males perform better in tests of high attentional load, whereas female WMI rats perform similar to their controls.

WMI animals did not show changes in cognitive flexibility when compared to controls as indicated by the amount of perseverative responses in the manipulations of the 5CSRTT. If anything, WMI animals seem to make less perseverative responses in the manipulations where the ITI or stimulus duration is variable. Performance in the probabilistic reversal learning test, the other measure of cognitive flexibility, was similar between WMI and control animals. The ability to process information due to changes in the task was equal between control and WMI animals as reflected by the latency to make a correct choice. In the reversal learning task, WMI females were faster in responding compared to female controls, whereas male WMI and controls were equally fast. In 4 out of 10 manipulations in the 5CSRTT, WMI animals were faster in collecting the reward, whereas in the other tests no differences were found. This indicates that WMI animals are at least as motivated to perform in the task as control animals. This is also supported by the lack of differences in performance in the task when animals had access to ad libitum food at least 48 h prior to testing. Taken together, based on performance on the 5CSRTT and probabilistic reversal learning task, no major deficits in functional outcome were observed in adult WMI rats.

### Effect of manipulating parameters in the 5CSRTT

The manipulations of the 5CSRTT presented in this paper were chosen to assess impulsivity, motivation, attention and cognitive flexibility. In addition, by manipulating task contingencies, an animal’s ability to react on a changing environment is assessed. The value of the 5CSRTT is that this paradigm has a human analogue, the Continuous Performance Task (CPT) (Chudasama and Robbins 2004) and more recently the 4CSRTT (Voon et al. 2014) and therefore provides high construct validity as a direct measure of attention and inhibitory control, i.e. impulsivity.

Elongating the ITI increased premature responses and decreased in accuracy, indicating that animals have difficulties in inhibitory control (Bari et al. 2008; Robbins 2002; Evenden 1999). The effects were stronger in the variable version of the task (Robbins 2002; Bari et al. 2008; Dalley et al. 2005a, b; Davies et al. 2009; Mirza and Stolerman 1998; Stolerman et al. 2000). In the short (variable) ITI manipulations, the animals have to make choices in a high frequency (High Event Rate, high attentional demand). In line with the literature, less premature responses, more omitted trials and an increase in choice latency were observed (Robbins 2002; Bari et al. 2008; Baardense et al. 2013; Asinof and Paine 2014). In contrast to Robbins (2002) and Bari et al. (2008), no negative effects on accuracy were found. Reducing the stimulus duration decreased accuracy and increased omissions, in line with several studies (Asinof and Paine 2014; Amitai and Markou 2011; Bari et al. 2008); however, in our study, animals also made more premature responses and the latency to a correct response was reduced. In line with Van der Veen et al. (2015), distractors resulted in reduced accuracy, more omitted trials and increased response latencies. Most studies use irrelevant visual stimuli or white noise as distractors (Amitai and Markou 2011; Robbins 2002). The continuous presence of the wooden block during testing could account for increased response and reward collect times by demanding a lot of the animal’s ability to ignore irrelevant information.

In sum, the animals in the present study reacted on changing contingencies in the task in similar ways as described in the literature, with little additional effects of WMI and sex.

### Sex differences in the 5CSRTT and the reversal learning task

Overall, males and females performed similar in the 5CSRTT and the reversal learning task, indicating that cognitive abilities are similar in male and female rats (Roberts et al. 2019). In 7 out of 10 manipulations in the 5CSRTT, female rats showed more perseverative responses (indicator of cognitive inflexibility) compared to male rats while the other parameters were similar. This might suggest that females have a different strategy in response to changes in the environment rather than problems with cognitive flexibility in the 5CRTT. In support of this explanation, females did not differ from males in their perseverance in the probabilistic reversal task. Sustained visual attention manipulations in the 5CSRTT resulted in less omitted trials in female control rats compared to male control rats but seemed opposite in WMI rats, indicating that in control conditions females perform better in tasks of high attentional load. In contrast to our findings, literature on sex differences in 5CRTT performance reports that females, more so than males, showed impairments in attention and omissions in the 5CSRTT (Jentsch and Taylor 2003; Bayless et al. 2012). In the probabilistic reversal learning task, overall performance was the same between the sexes, although the estrous cycle was found to affect some but not all parameters (Verharen et al. 2019). In the present study, estrous cycle was not taken into account; however, our data support the notion that overall performance in this task does not differ between the sexes. Since literature directly comparing males and females in the 5-choice task and probabilistic reversal is limited, our data adds to the literature that females react differently to males only in specific manipulations of the task and at different parameters.

### The WMI animals show no behavioural pattern reminiscent of PFC impairment

In our previous study, WMI animals were shown to exhibit impaired motor performance, anxiety-like behaviour and signs of autism-like behaviour, including reduced social play behaviour and repetitive grooming. No changes were observed in working memory at the age of 6 weeks or object recognition memory at 9 weeks, indicating no severe memory deficits (Van Tilborg et al. 2018a, b). The performance of WMI animals in more cognitive demanding tasks as evaluated in this study was subtly different but these findings do not seem to reflect a PFC impairment.

The PFC plays a role in impulse control, cognitive flexibility and attention, by selecting relevant information so that the appropriate behaviour is executed (Buschman and Miller 2007; Noudoost et al. 2010; Saalmann et al. 2007). Lesioning, pharmacological inactivation or optogenetic inhibition of the prelimbic (PrL) and infralimbic (IL) subregions of the PFC impaired accuracy (Muir et al. 1996; Chadusmasa and Muir 2001; Chudasama and Robbins 2003; Passetti et al. 2002; Dalton et al. 2016; Luchicchi et al. 2016; Izquierdo and Jentsch 2012; Hervig et al. 2019), whereas IL inactivation impaired inhibitory control in the 5CSRTT (Hosking et al. 2016). The probabilistic reversal learning test is dependent on the orbitofrontal (OFC) regions of the PFC (Birrell and Brown 2000; Chudasama and Robbins 2003; McAlonan and Brown 2003; Ghods-Sharifi et al. 2008; Izquierdo and Jentsch 2012). Lesioning or pharmacological inactivation of (subregions of) the OFC impairs reversal learning (Chudasama and Robbins 2003; Dalton et al. 2016). A recent study by Verharen et al. (2020) confirmed the involvement of OFC subregions in this task but also showed that inactivating the PrL and IL affected performance. The WMI animals in this study, although a slightly different version of the task is used, did not show any disruption in reversal learning.

Behavioural patterns resulting from directly affecting the PFC do not resemble that of the WMI animals in the present study, which can be attributed to unaffected PFC myelination in WMI animals at the time of testing. Our myelin stainings show that the PFC remains relatively spared following fetal inflammation and postnatal hypoxia, both in adolescence (i.e. P30) and adulthood (i.e. after the tests). This might be explained by the fact that PFC maturation and myelination occur later in development compared to more caudal brain regions (Van Eden et al. 1990). In addition, our stainings show that myelin deficits observed in the sensory cortex at P30 in WMI animals did also recover at the age of 9 months. Together, these data indicate that the two-hit model of WMI elicits transient myelin deficits in brain regions more posterior to the prefrontal cortex (Van Tilborg et al. 2018b). While our immunofluorescent analyses did not show any overt myelin deficits in the PFC, we cannot rule out that subtle differences in behavioural paradigms could also be explained by differences in myelin quality or compactness of axonal wrapping (i.e. myelin g-ratio), which could be addressed by for instance electron microscopy.

Besides myelination, other neurodevelopmental processes may also be affected by the two-hit model, and could be responsible for the subtle differences between control and WMI animals. One example is the generation and migration of GABAergic interneurons, which coincides with the time window during which preterm infants are exposed to various perinatal insults (Robinson et al. 2006). Both in rodent models of neonatal WMI and in human tissue of preterm infants with WMI, a reduction in inhibitory GABAergic neurons has been found (Komitova et al. 2013; Robinson et al. 2006). Most recently, we showed that the number of PV-positive interneurons was transiently reduced in the cortex and long-lasting reduced in the hippocampal area after this double-hit model of WMI in rats (Vaes et al. 2021). Disrupted inhibitory to excitatory (E/I) balance in the brain has been associated with neurodevelopmental disorders such as autism spectrum disorder and schizophrenia where cognitive abilities are impaired (Sohal and Rubenstein, 2019). Another neurodevelopmental process that occurs during early postnatal brain development is synaptic pruning by microglia (Paolicelli et al. 2011). During this process, microglia phagocytose excess neuronal synapses, which is crucial for normal (E/I) balance in the developing brain (Paolicelli et al. 2011). Activation of microglia during brain development may cause excessive or insufficient synaptic pruning, which may explain the link between early inflammation and neurodevelopmental disorders (Neniskyte and Gross 2017). Taken together, these studies indicate that besides myelination deficits, neural circuitry may also be affected by the two-hit model, especially by fetal inflammation at E18/19, and might at least partly explain the subtle differences between WMI and control animals in the present study as well as the behavioural abnormalities in the previous study (van Tilborg et al. 2018a).

### Clinical relevance of rodent dWMI models

As the incidence of preterm birth is still ominously high and the consequences of prematurity lead to serious life-long neurodevelopmental impairments, in recent years much effort has been made to develop relevant animal models that mimic non-cystic diffuse WMI, the most commonly observed form of brain injury in (extreme) preterm infants. Whereas most models apply only a single inflammatory or a single hypoxic insult to induce WMI, multiple-hit models are now acknowledged to mimic the complex etiology and pathophysiology of dWMI most closely (Silbereis et al. 2010; for review see Zeng et al. 2018; Kaindl et al. 2009; Van Steenwinckel et al. 2014; Vaes et al. 2020). Besides representing the anatomical deficits in the target patient (i.e. diffuse patterns of myelination deficits), animal models gain in value when functional impairments as observed in preterm infants are also represented.

Numerous clinical studies, systemic reviews and meta-analyses have established that preterm birth is associated with negative neurodevelopmental outcome during childhood into adolescence and adulthood (up to 36 years of age), with lower gestational age at birth increasing the risk (Allotey et al. 2018; Hack et al. 2002; van Houdt et al. 2016; Kroll et al. 2017; Lundequist et al. 2015; Lærum et al. 2017; Moster et al. 2008; O’Reilly et al. 2020; Stålnacke et al. 2019; Taylor and Clark 2016; Van Tilborg et al. 2018a, b). Children born very or extremely preterm show lower cognitive abilities (e.g. particularly lower executive functioning including cognitive inflexibility and lower inhibition control, lower IQ and lower academic achievements) and an increased risk of developing psychiatric disorders like attention-deficit hyperactivity disorder (ADHD), autism spectrum disorders and schizophrenia than children born at term (Ask et al. 2018; Franz et al. 2018; Galéra et al. 2011).

Interestingly, in terms of personality, young (19–26 years old) adults born prematurely show more internalizing, avoidant and social problems (i.e. anxiety, withdrawal, lack of self-confidence) and less externalizing (i.e. aggressive, intrusive, rule-breaking behaviours) compared to peers born at term (Pyhala et al. 2017). Follow-up studies testing preterm infants from (pre-) school age into adolescence and adulthood have demonstrated that cognitive abilities (e.g. executive functioning) measured at school age are very predictive for functioning later in life, showing that deficits remain stable over time without significant catch-up (Lundequist et al. 2015; Stålnacke et al. 2019; Van Houdt et al. 2019).

Previously, we showed that our double-hit rat model leads to myelination deficits (at least until P30) and impaired functional outcome including motor deficits, anxiety-related and autism-like behaviour (Van Tilborg et al. 2018b). The current study shows that only subtle differences were observed in the 5CSRTT and reversal learning task. In other words, our double-hit model did not induce overt deficits in cognitive functioning during adulthood, thereby not reflecting clinical observations regarding impaired cognitive performance that persists throughout adulthood. Our data indicate that the neonatal rat model either causes only transient functional deficits, or the higher cognitive functional domains or corresponding brain regions required for these cognitive tasks are not affected by the hits or timing of our model. No animal model completely mimics the pathophysiological changes in human preterm infants and the deficits observed in other rodent models of neonatal diffuse WMI seem to be transient as well. A rat model of maternal inflammation using LPS injections at E19 and E20 caused transient motor deficits in offspring which were restored to control levels after P23 (Rousset et al. 2013). Functional recovery observed in these rodent models could be explained by delayed rather than permanently damaged white matter as indicated by restored white matter integrity (Van Tilborg et al. 2018b; Rousset et al. 2013; Rideau Batista Novais et al. 2016). Alternatively, differences in plasticity between rodent and human newborn brains could explain why long-term deficits as observed in human preterm infants are not observed in rodent models of dWMI. Early postnatal rodent brains may respond to inflammatory and/or hypoxic conditions by promoting proliferation and differentiation of neural stem cells to compensate for injurious events. To illustrate this, Fagel et al. demonstrated in a mouse model that chronic perinatal hypoxia enhanced cortical neurogenesis and that 45% of newly generated cells became oligodendrocytes (Fagel et al. 2006). Similarly, following combined fetal inflammation and postnatal hypoxia, we observed increased proliferation of oligodendrocyte precursor cells (OPCs) in the white matter of WMI rats (Van Tilborg et al. 2018b). The increased generation of OPCs following perinatal insults may eventually compensate for the initial lack of myelination. Differences in oligodendrocyte turnover, which is relatively high in rodents and relatively low in humans, indicate that dynamics by which the oligodendrocyte pool is maintained and can respond to injury may be different between species, favoring (re)myelination in rodent models (van Tilborg et al. 2018a review). The differences in adult cognitive functioning between preterm human infants and rats that underwent fetal inflammation and postnatal hypoxia during the neonatal period may be interpreted as a limitation of the currently used rat model of diffuse WMI. However, so far no alternative rodent model has been developed that causes non-cystic diffuse WMI together with subtle cognitive impairment throughout adulthood. Whether the absence of long-term cognitive deficits in rodents exposes an inherent difference between the rodent vs. human brain, or whether alternative perinatal insults (e.g. more severe, systemic or repeated perinatal hits) may cause diffuse WMI in combination with long-term cognitive deficits to accurately reflect the clinical situation should be further elucidated.

## Conclusion

Using the five-choice serial reaction time task and the probabilistic reversal learning task, we demonstrate that combined fetal inflammation and early postnatal hypoxia does not substantially affect cognitive functioning in both male and female rats during adulthood. The absence of major cognitive deficits is explained by an absence of myelination deficits in prefrontal cortical subregions in both young and adult animals. We did find a myelination deficit in young animals at 30 days of age in the sensory cortex that was not observed anymore at 9 months of age. Our data adds and highlights the possibilities and limitations to the currently available diffuse WMI models. The model utilized in the present study can be used to develop novel treatment options aimed at relative early recovery after perinatal insults, which may improve myelination in specific brain regions, and may improve negatively affected aspects of functional outcome, like autism-like behaviour.

## Supplementary Information

Below is the link to the electronic supplementary material.

Supplementary file1 (DOCX 283 KB)

Supplementary file2 (DOCX 85 KB)
