# Supplementary material for: Cognitive performance during adulthood in a rat model of neonatal diffuse white matter injury
Source: Psychopharmacology (Berl). 2022 Jan 22;239(3):745–64. doi: 10.1007/s00213-021-06053-w (PMC8891199; doi:10.1007/s00213-021-06053-w)
Supplement: Supplementary file 1 — Supplementary file1 (DOCX 283 KB) [file 213_2021_6053_MOESM1_ESM.docx]

**Supplementary material**

**Cognitive performance during adulthood in a rat model of neonatal diffuse white matter injury.**

Psychopharmacology

EJ Marijke Achterberg, Ralf J van Oldeniel, Erik van Tilborg, Jeroen P.H. Verharen, Cora H Nijboer, Louk JMJ Vanderschuren

Corresponding author

E.J.M. Achterberg, Department of Population Health Sciences, Unit Animals in Science and Society, Division of Behavioural Neuroscience, Faculty of Veterinary Medicine, Utrecht University, Yalelaan 2, 3584CM, Utrecht. The Netherlands. Email: e.j.m.achterberg@uu.nl

**Table 1: Training**

| **Parameters** | |  |  |  |  |  |  | **Statistics** |  |  |  |  |
| --- | --- | --- | --- | --- | --- | --- | --- | --- | --- | --- | --- | --- |
| **Accurary** | |  |  |  |  |  |  |  |  |  |  |  |
|  |  |  | **Start training** | | **End training** | |  | **Factors** | **df** | **df** | **F** | **Sig.** |
| **N** | **Sex** | **Treatment** | **Mean** | **STDV** | **Mean** | **STDV** |  | *Training* | *1* | *44* | *23.36* | *p<0.001* |
| 13 | Male | Contol | 83.15 | 15.89 | 93.62 | 3.98 |  | Sex | 1 | 44 | 0.04 | 0.85 |
| 12 |  | WMI | 84.58 | 15.89 | 95.00 | 3.98 |  | Treatment | 1 | 44 | 2.66 | 0.11 |
| 15 | Female | Contol | 76.00 | 15.89 | 94.33 | 3.98 |  | Sex * Treatment | 1 | 44 | 1.16 | 0.29 |
| 8 |  | WMI | 89.38 | 15.89 | 94.75 | 3.98 |  | Training * Sex | 1 | 44 | 0.09 | 0.76 |
|  |  |  |  |  |  |  |  | Training * Treatment | 1 | 44 | 1.99 | 0.17 |
|  |  |  |  |  |  |  |  | Training * Sex * Treatment | 1 | 44 | 1.96 | 0.17 |
| **Omissions** | |  |  |  |  |  |  |  |  |  |  |  |
|  |  |  | **Start training** | | **End training** | |  | **Factors** | **df** | **df** | **F** | **Sig.** |
| **N** | **Sex** | **Treatment** | **Mean** | **STDV** | **Mean** | **STDV** |  | Training | 1 | 44 | 3.54 | 0.07 |
| 13 | Male | Control | 3.69 | 9.20 | 12.77 | 4.51 |  | Sex | 1 | 44 | 1.15 | 0.29 |
| 12 |  | WMI | 7.75 | 9.20 | 7.00 | 4.52 |  | Treatment | 1 | 44 | 0.26 | 0.61 |
| 15 | Female | Control | 5.73 | 9.21 | 7.33 | 4.52 |  | Sex * Treatment | 1 | 44 | 0.00 | 0.95 |
| 8 |  | WMI | 5.00 | 9.20 | 6.75 | 4.51 |  | Training * Sex | 1 | 44 | 0.64 | 0.43 |
|  |  |  |  |  |  |  |  | Training * Treatment | 1 | 44 | 2.43 | 0.13 |
|  |  |  |  |  |  |  |  | Training * Sex * Treatment | 1 | 44 | 2.59 | 0.12 |

**Table 2: Ad libitum food**

| **Parameters** | |  |  |  |  |  |  | **Statistics** |  |  |  |  |
| --- | --- | --- | --- | --- | --- | --- | --- | --- | --- | --- | --- | --- |
| **Accurary** | |  |  |  |  |  |  |  |  |  |  |  |
|  |  |  | **Baseline** | | **Test** |  |  | **Factors** | **df** | **df** | **F** | **Sig.** |
| **N** | **Sex** | **Treatment** | **Mean** | **STDV** | **Mean** | **STDV** |  | Test | 1 | 44 | 0.28 | 0.60 |
| 13 | Male | Control | 93.16 | 4.49 | 94.35 | 5.30 |  | Sex | 1 | 44 | 0.08 | 0.78 |
| 12 |  | WMI | 95.50 | 3.82 | 96.61 | 3.37 |  | Treatment | 1 | 44 | 3.19 | 0.08 |
| 15 | Female | Control | 93.79 | 4.11 | 93.91 | 3.58 |  | Test * Sex | 1 | 44 | 0.14 | 0.71 |
| 8 |  | WMI | 96.01 | 3.03 | 94.70 | 2.91 |  | Test * Treatment | 1 | 44 | 2.82 | 0.10 |
|  |  |  |  |  |  |  |  | Sex * Treatment | 1 | 44 | 0.52 | 0.47 |
|  |  |  |  |  |  |  |  | Test * Sex * Treatment | 1 | 44 | 0.41 | 0.52 |
| **Premature** | |  |  |  |  |  |  |  |  |  |  |  |
|  |  |  | **Baseline** | | **Test** |  |  | **Factors** | **df** | **df** | **F** | **Sig.** |
| **N** | **Sex** | **Treatment** | **Mean** | **STDV** | **Mean** | **STDV** |  | *Test* | *1* | *44* | *22.52* | *<0.001* |
| 13 | Male | Control | 5.62 | 4.46 | 2.00 | 4.06 |  | Sex | 1 | 44 | 2.58 | 0.12 |
| 12 |  | WMI | 3.00 | 2.59 | 1.25 | 1.06 |  | Treatment | 1 | 44 | 0.77 | 0.38 |
| 15 | Female | Control | 2.53 | 2.20 | 0.73 | 0.80 |  | Sex * Treatment | 1 | 44 | 2.58 | 0.12 |
| 8 |  | WMI | 3.00 | 3.21 | 1.25 | 1.04 |  | Test * Sex | 1 | 44 | 0.93 | 0.34 |
|  |  |  |  |  |  |  |  | Test * Treatment | 1 | 44 | 1.04 | 0.31 |
|  |  |  |  |  |  |  |  | Test * Sex * Treatment | 1 | 44 | 0.93 | 0.34 |
| **Omissions** | |  |  |  |  |  |  |  |  |  |  |  |
|  |  |  | **Baseline** | | **Test** |  |  | **Factors** | **df** |  | **F** | **Sig.** |
| **N** | **Sex** | **Treatment** | **Mean** | **STDV** | **Mean** | **STDV** |  | *Test* | *1* | *44* | *55.95* | *<0.001* |
| 13 | Male | Control | 5.00 | 2.68 | 18.08 | 11.08 |  | Sex | 1 | 44 | 0.52 | 0.47 |
| 12 |  | WMI | 3.08 | 2.43 | 21.00 | 22.53 |  | Treatment | 1 | 44 | 0.13 | 0.72 |
| 15 | Female | Control | 5.53 | 4.07 | 20.13 | 7.83 |  | Sex * Treatment | 1 | 44 | 0.02 | 0.90 |
| 8 |  | WMI | 5.63 | 5.45 | 22.13 | 8.34 |  | Test * Sex | 1 | 44 | 0.00 | 0.99 |
|  |  |  |  |  |  |  |  | Test * Treatment | 1 | 44 | 0.66 | 0.42 |
|  |  |  |  |  |  |  |  | Test * Sex * Treatment | 1 | 44 | 0.13 | 0.73 |
| **Perserverant** | |  |  |  |  |  |  |  |  |  |  |  |
|  |  |  | **Baseline** | | **Test** |  |  | **Factors** | **df** | **df** | **F** | **Sig.** |
| **N** | **Sex** | **Treatment** | **Mean** | **STDV** | **Mean** | **STDV** |  | *Test* | 1 | 44 | 0.33 | 0.57 |
| 13 | Male | Control | 5.92 | 5.60 | 6.77 | 4.99 |  | Sex | 1 | 44 | 3.01 | 0.09 |
| 12 |  | WMI | 2.67 | 2.02 | 4.42 | 4.08 |  | Treatment | 1 | 44 | 1.27 | 0.27 |
| 15 | Female | Control | 8.33 | 8.67 | 7.87 | 7.02 |  | Sex * Treatment | 1 | 44 | 0.40 | 0.53 |
| 8 |  | WMI | 7.63 | 4.47 | 7.00 | 5.15 |  | Test * Sex | 1 | 44 | 1.96 | 0.17 |
|  |  |  |  |  |  |  |  | Test * Treatment | 1 | 44 | 0.08 | 0.78 |
|  |  |  |  |  |  |  |  | Test * Sex * Treatment | 1 | 44 | 0.16 | 0.69 |

| **Parameters** | |  |  |  |  |  |  | **Statistics** |  |  |  |  |
| --- | --- | --- | --- | --- | --- | --- | --- | --- | --- | --- | --- | --- |
| **Latency to correct** | | |  |  |  |  |  |  |  |  |  |  |
|  |  |  | **Baseline** | | **Test** |  |  | **Factors** | **df** | **df** | **F** | **Sig.** |
| **N** | **Sex** | **Treatment** | **Mean** | **STDV** | **Mean** | **STDV** |  | *Test* | *1* | *44* | *98.70* | *<0.001* |
| 13 | Male | Control | 0.61 | 0.10 | 0.72 | 0.09 |  | Sex | 1 | 44 | 0.13 | 0.72 |
| 12 |  | WMI | 0.63 | 0.13 | 0.75 | 0.23 |  | Treatment | 1 | 44 | 0.00 | 0.99 |
| 15 | Female | Control | 0.63 | 0.09 | 0.78 | 0.08 |  | Sex * Treatment | 1 | 44 | 0.49 | 0.49 |
| 8 |  | WMI | 0.59 | 0.10 | 0.77 | 0.15 |  | Test * Sex | 1 | 44 | 3.05 | 0.09 |
|  |  |  |  |  |  |  |  | Test * Treatment | 1 | 44 | 0.18 | 0.67 |
|  |  |  |  |  |  |  |  | Test * Sex * Treatment | 1 | 44 | 0.17 | 0.68 |
| **Latency to reward** | | |  |  |  |  |  |  |  |  |  |  |
|  |  |  | **Baseline** | | **Test** |  |  | **Factors** | **df** | **df** | **F** | **Sig.** |
| **N** | **Sex** | **Treatment** | **Mean** | **STDV** | **Mean** | **STDV** |  | *Test* | *1* | *44* | *9.74* | *0.003* |
| 13 | Male | Control | 1.81 | 0.71 | 3.80 | 3.11 |  | Sex | 1 | 44 | 2.25 | 0.14 |
| 12 |  | WMI | 1.40 | 0.34 | 5.35 | 7.34 |  | Treatment | 1 | 44 | 0.31 | 0.58 |
| 15 | Female | Control | 1.59 | 0.62 | 2.46 | 1.60 |  | Sex * Treatment | 1 | 44 | 0.10 | 0.76 |
| 8 |  | WMI | 1.84 | 1.90 | 2.53 | 1.13 |  | Test * Sex | 1 | 44 | 3.33 | 0.08 |
|  |  |  |  |  |  |  |  | Test * Treatment | 1 | 44 | 0.55 | 0.46 |
|  |  |  |  |  |  |  |  | Test * Sex * Treatment | 1 | 44 | 0.79 | 0.38 |

**Table 3: 2 seconds (short) ITI**

| **Parameters** | |  |  |  |  |  |  | **Statistics** |  |  |  |  |
| --- | --- | --- | --- | --- | --- | --- | --- | --- | --- | --- | --- | --- |
| **Accurary** | |  |  |  |  |  |  |  |  |  |  |  |
|  |  |  | **Baseline** | | **Test** |  |  | **Factors** | **df** | **df** | **F** | **Sig.** |
| **N** | **Sex** | **Treatment** | **Mean** | **STDV** | **Mean** | **STDV** |  | *Test* | *1* | *44* | *34.67* | *<0.001* |
| 13 | Male | Control | 92.57 | 4.59 | 97.31 | 3.27 |  | Sex | 1 | 44 | 0.00 | 0.96 |
| 12 |  | WMI | 91.54 | 6.16 | 97.64 | 1.56 |  | Treatment | 1 | 44 | 0.16 | 0.69 |
| 15 | Female | Control | 92.81 | 5.78 | 97.34 | 2.86 |  | Sex * Treatment | 1 | 44 | 0.01 | 0.94 |
| 8 |  | WMI | 93.30 | 5.55 | 95.83 | 3.56 |  | Test * Sex | 1 | 44 | 1.55 | 0.22 |
|  |  |  |  |  |  |  |  | Test * Treatment | 1 | 44 | 0.05 | 0.83 |
|  |  |  |  |  |  |  |  | Test * Sex * Treatment | 1 | 44 | 1.23 | 0.27 |
| **Premature** | |  |  |  |  |  |  |  |  |  |  |  |
|  |  |  | **Baseline** | | **Test** |  |  | **Factors** | **df** | **df** | **F** | **Sig.** |
| **N** | **Sex** | **Treatment** | **Mean** | **STDV** | **Mean** | **STDV** |  | *Test* | *1* | *44* | *84.55* | *<0.001* |
| 13 | Male | Control | 7.69 | 5.66 | 0.38 | 1.12 |  | *Sex* | *1* | *44* | *6.74* | *0.01* |
| 12 |  | WMI | 4.92 | 3.58 | 0.42 | 0.90 |  | Treatment | 1 | 44 | 1.63 | 0.21 |
| 15 | Female | Control | 3.53 | 2.03 | 0.20 | 0.41 |  | Sex * Treatment | 1 | 44 | 0.97 | 0.33 |
| 8 |  | WMI | 3.38 | 1.30 | 0.00 | 0.00 |  | *Test * Sex* | *1* | *44* | *6.41* | *0.02* |
|  |  |  |  |  |  |  |  | Test * Treatment | 1 | 44 | 1.89 | 0.18 |
|  |  |  |  |  |  |  |  | Test * Sex * Treatment | 1 | 44 | 2.00 | 0.16 |
| **Omissions** | |  |  |  |  |  |  |  |  |  |  |  |
|  |  |  | **Baseline** | | **Test** |  |  | **Factors** | **df** | **df** | **F** | **Sig.** |
| **N** | **Sex** | **Treatment** | **Mean** | **STDV** | **Mean** | **STDV** |  | *Test* | *1* | *44* | *53.05* | *<0.001* |
| 13 | Male | Control | 9.54 | 4.18 | 20.69 | 7.35 |  | Sex | 1 | 44 | 0.01 | 0.93 |
| 12 |  | WMI | 6.00 | 4.57 | 12.92 | 8.24 |  | Treatment | 1 | 44 | 0.09 | 0.76 |
| 15 | Female | Control | 6.93 | 5.43 | 13.27 | 6.45 |  | *Sex * Treatment* | *1* | *44* | *9.72* | *0.003* |
| 8 |  | WMI | 7.75 | 6.56 | 21.75 | 13.82 |  | Test * Sex | 1 | 44 | 0.18 | 0.67 |
|  |  |  |  |  |  |  |  | Test * Treatment | 1 | 44 | 0.42 | 0.52 |
|  |  |  |  |  |  |  |  | *Test * Sex * Treatment* | *1* | *44* | *5.10* | *0.03* |
| **Preserverative responses** | | | |  |  |  |  |  |  |  |  |  |
|  |  |  | **Baseline** | | **Test** |  |  | **Factors** | **df** | **df** | **F** | **Sig.** |
| **N** | **Treatment** | **Sex** | **Mean** | **STDV** | **Mean** | **STDV** |  | Test | 1 | 44 | 0.43 | 0.52 |
| 13 | Control | Male | 5.08 | 4.75 | 5.31 | 5.56 |  | Sex | 1 | 44 | 1.91 | 0.17 |
| 15 |  | Female | 6.47 | 4.79 | 8.93 | 7.54 |  | Treatment | 1 | 44 | 3.70 | 0.06 |
| 12 | WMI | Male | 2.83 | 1.95 | 3.33 | 2.96 |  | Sex * Treatment | 1 | 44 | 0.17 | 0.68 |
| 8 |  | Female | 5.00 | 8.86 | 3.88 | 4.39 |  | Test * Sex | 1 | 44 | 1.10 | 0.30 |
|  |  |  |  |  |  |  |  | Test * Treatment | 1 | 44 | 0.04 | 0.85 |
|  |  |  |  |  |  |  |  | Test * Sex * Treatment | 1 | 44 | 1.48 | 0.23 |

| **Parameters** | |  |  |  |  |  |  | **Statistics** |  |  |  |  |
| --- | --- | --- | --- | --- | --- | --- | --- | --- | --- | --- | --- | --- |
| **Latency to correct** | | |  |  |  |  |  |  |  |  |  |  |
|  |  |  | **Baseline** | | **Test** |  |  | **Factors** | **df** | **df** | **F** | **Sig.** |
| **N** | **Sex** | **Treatment** | **Mean** | **STDV** | **Mean** | **STDV** |  | *Test* | *1* | *44* | *100.90* | *<0.001* |
| 13 | Male | Control | 0.63 | 0.05 | 0.74 | 0.11 |  | Sex | 1 | 44 | 0.09 | 0.77 |
| 12 |  | WMI | 0.61 | 0.12 | 0.73 | 0.16 |  | Treatment | 1 | 44 | 0.61 | 0.44 |
| 15 | Female | Control | 0.63 | 0.08 | 0.73 | 0.11 |  | Sex * Treatment | 1 | 44 | 0.05 | 0.83 |
| 8 |  | WMI | 0.58 | 0.08 | 0.73 | 0.12 |  | Test * Sex | 1 | 44 | 0.33 | 0.57 |
|  |  |  |  |  |  |  |  | Test * Treatment | 1 | 44 | 2.19 | 0.15 |
|  |  |  |  |  |  |  |  | Test * Sex * Treatment | 1 | 44 | 0.52 | 0.47 |
| **Latency to reward** | | |  |  |  |  |  |  |  |  |  |  |
|  |  |  | **Baseline** | | **Test** |  |  | **Factors** | **df** | **df** | **F** | **Sig.** |
| **N** | **Sex** | **Treatment** | **Mean** | **STDV** | **Mean** | **STDV** |  | Test | 1 | 44 | 0.71 | 0.40 |
| 13 | Male | Control | 1.85 | 0.62 | 1.96 | 0.70 |  | Sex | 1 | 44 | 0.29 | 0.59 |
| 12 |  | WMI | 1.53 | 0.50 | 1.86 | 0.85 |  | Treatment | 1 | 44 | 0.00 | 0.99 |
| 15 | Female | Control | 1.58 | 0.69 | 1.56 | 0.51 |  | Sex * Treatment | 1 | 44 | 0.77 | 0.38 |
| 8 |  | WMI | 2.34 | 3.05 | 1.22 | 0.43 |  | Test * Sex | 1 | 44 | 3.68 | 0.06 |
|  |  |  |  |  |  |  |  | Test * Treatment | 1 | 44 | 1.11 | 0.30 |
|  |  |  |  |  |  |  |  | Test * Sex * Treatment | 1 | 44 | 2.57 | 0.12 |

**Post hoc tests**

| **Premature** | |  |  |  |  |  |  |  |  |  | **Bonferroni adjustment: p<0.0125** | | | |  |
| --- | --- | --- | --- | --- | --- | --- | --- | --- | --- | --- | --- | --- | --- | --- | --- |
|  |  | **Male** | |  |  | **Female** | |  |  |  |  | **df** | **df** | **F** | **Sig.** |
| **Factors** |  | **df** | **df** | **F** | **Sig.** | **df** | **df** | **F** | **Sig.** |  | ***Baseline: M vs F*** | *1* | *46* | *7.11* | *0.01* |
| *Test* |  | *1* | *23* | *45.92* | *<0.001* | *1* | *21* | *69.24* | *<0.001* |  | **Test: M vs F** | 1 | 46 | 1.51 | 0.23 |
| Treatment | | 1 | 23 | 1.63 | 0.22 | 1 | 21 | 0.19 | 0.67 |  |  |  |  |  |  |
| Test * Treatment | | 1 | 23 | 2.60 | 0.12 | 1 | 21 | 0.00 | 0.96 |  |  |  |  |  |  |
|  |  |  |  |  |  |  |  |  |  |  |  |  |  |  |  |
|  |  |  |  |  |  |  |  |  |  |  |  |  |  |  |  |
| **Omissions** | |  |  |  |  |  |  |  |  |  | **Bonferroni adjustment: p<0.0125** | | | |  |
|  |  | **Baseline** | |  |  | **Test** |  |  |  |  | **Test** | **t** | **df** | **Sig.** |  |
| **Factors** |  | **df** | **df** | **F** | **Sig.** | **df** | **df** | **F** | **Sig.** |  | **M: ctrl vs WMI** | 2.49 | 23 | 0.02 |  |
| Sex |  | 1 | 44 | 0.08 | 0.78 | 1 | 44 | 0.08 | 0.79 |  | **F: ctrl vs WMI** | -2.03 | 21 | 0.06 |  |
| Treatment | | 1 | 44 | 0.80 | 0.38 | 1 | 44 | 0.02 | 0.89 |  | ***Ctrl: M vs F*** | *2.85* | *26* | *0.01* |  |
| Sex * Treatment | | 1 | 44 | 2.06 | 0.16 | *1* | *44* | *9.97* | *0.003* |  | **WMI: M vs F** | -1.80 | 18 | 0.09 |  |

**Table 4: Short variable ITI (1,2 and 3 sec)**

| **Parameters** | |  |  |  |  |  |  | **Statistics** |  |  |  |  |
| --- | --- | --- | --- | --- | --- | --- | --- | --- | --- | --- | --- | --- |
| **Accurary** | |  |  |  |  |  |  |  |  |  |  |  |
|  |  |  | **Baseline** | | **Test** |  |  | **Factors** | **df** | **df** | **F** | **Sig.** |
| **N** | **Sex** | **Treatment** | **Mean** | **STDV** | **Mean** | **STDV** |  | Test | 1 | 44 | 2.51 | 0.12 |
| 13 | Male | Control | 93.35 | 4.39 | 94.32 | 4.60 |  | Sex | 1 | 44 | 0.17 | 0.68 |
| 12 |  | WMI | 95.13 | 3.43 | 95.76 | 3.62 |  | Treatment | 1 | 44 | 0.08 | 0.78 |
| 15 | Female | Control | 93.83 | 3.77 | 95.67 | 3.75 |  | Sex * Treatment | 1 | 44 | 1.81 | 0.19 |
| 8 |  | WMI | 93.79 | 2.46 | 93.60 | 2.73 |  | Test * Sex | 1 | 44 | 0.00 | 0.98 |
|  |  |  |  |  |  |  |  | Test * Treatment | 1 | 44 | 1.33 | 0.26 |
|  |  |  |  |  |  |  |  | Test * Sex * Treatment | 1 | 44 | 0.68 | 0.41 |
| **Premature** | |  |  |  |  |  |  |  |  |  |  |  |
|  |  |  | **Baseline** | | **Test** |  |  | **Factors** | **df** | **df** | **F** | **Sig.** |
| **N** | **Sex** | **Treatment** | **Mean** | **STDV** | **Mean** | **STDV** |  | *Test* | *1* | *44* | *15.46* | *<0.001* |
| 13 | Male | Control | 6.46 | 4.86 | 3.46 | 2.63 |  | Sex | 1 | 44 | 1.63 | 0.21 |
| 12 |  | WMI | 5.42 | 4.96 | 3.25 | 2.93 |  | Treatment | 1 | 44 | 0.16 | 0.69 |
| 15 | Female | Control | 5.20 | 4.59 | 1.87 | 1.77 |  | Sex * Treatment | 1 | 44 | 0.09 | 0.77 |
| 8 |  | WMI | 4.13 | 4.22 | 2.75 | 1.98 |  | Test * Sex | 1 | 44 | 0.03 | 0.86 |
|  |  |  |  |  |  |  |  | Test * Treatment | 1 | 44 | 1.24 | 0.27 |
|  |  |  |  |  |  |  |  | Test * Sex * Treatment | 1 | 44 | 0.20 | 0.66 |
| **Omissions** | |  |  |  |  |  |  |  |  |  |  |  |
|  |  |  | **Baseline** | | **Test** |  |  | **Factors** | **df** | **df** | **F** | **Sig.** |
| **N** | **Sex** | **Treatment** | **Mean** | **STDV** | **Mean** | **STDV** |  | *Test* | *1* | *44* | *44.15* | *<0.001* |
| 13 | Male | Control | 7.00 | 4.18 | 15.38 | 6.78 |  | Sex | 1 | 44 | 0.62 | 0.44 |
| 12 |  | WMI | 4.67 | 2.96 | 10.00 | 5.13 |  | Treatment | 1 | 44 | 0.10 | 0.75 |
| 15 | Female | Control | 5.87 | 3.31 | 7.73 | 4.51 |  | *Sex * Treatment* | *1* | *44* | *9.52* | *0.004* |
| 8 |  | WMI | 6.38 | 4.63 | 13.50 | 6.32 |  | Test * Sex | 1 | 44 | 1.91 | 0.17 |
|  |  |  |  |  |  |  |  | Test * Treatment | 1 | 44 | 0.42 | 0.52 |
|  |  |  |  |  |  |  |  | *Test * Sex * Treatment* | *1* | *44* | *5.91* | *0.02* |
| **Preserverative responses** | | | |  |  |  |  |  |  |  |  |  |
|  |  |  | **Baseline** | | **Test** |  |  | **Factors** | **df** | **df** | **F** | **Sig.** |
| **N** | **Sex** | **Treatment** | **Mean** | **STDV** | **Mean** | **STDV** |  | Test | 1 | 44 | 0.91 | 0.35 |
| 13 | Male | Control | 5.62 | 4.44 | 6.38 | 3.66 |  | *Sex* | *1* | *44* | *5.39* | *0.03* |
| 12 |  | WMI | 3.58 | 4.87 | 3.33 | 2.02 |  | *Treatment* | *1* | *44* | *6.17* | *0.02* |
| 15 | Female | Control | 11.27 | 10.14 | 14.00 | 11.81 |  | Sex * Treatment | 1 | 44 | 1.33 | 0.26 |
| 8 |  | WMI | 5.63 | 3.42 | 5.75 | 5.97 |  | Test * Sex | 1 | 44 | 0.44 | 0.51 |
|  |  |  |  |  |  |  |  | Test * Treatment | 1 | 44 | 1.05 | 0.31 |
|  |  |  |  |  |  |  |  | Test * Sex * Treatment | 1 | 44 | 0.20 | 0.66 |

| **Parameters** | |  |  |  |  |  |  | **Statistics** |  |  |  |  |
| --- | --- | --- | --- | --- | --- | --- | --- | --- | --- | --- | --- | --- |
| **Latency to correct** | | |  |  |  |  |  |  |  |  |  |  |
|  |  |  | **Baseline** | | **Test** |  |  | **Factors** | **df** | **df** | **F** | **Sig.** |
| **N** | **Sex** | **Treatment** | **Mean** | **STDV** | **Mean** | **STDV** |  | *Test* | *1* | *44* | *34.34* | *<0.001* |
| 13 | Male | Control | 0.64 | 0.06 | 0.66 | 0.08 |  | Sex | 1 | 44 | 0.65 | 0.42 |
| 12 |  | WMI | 0.63 | 0.10 | 0.70 | 0.14 |  | Treatment | 1 | 44 | 0.02 | 0.88 |
| 15 | Female | Control | 0.62 | 0.07 | 0.66 | 0.07 |  | Sex * Treatment | 1 | 44 | 0.06 | 0.81 |
| 8 |  | WMI | 0.61 | 0.09 | 0.66 | 0.05 |  | Test * Sex | 1 | 44 | 0.00 | 0.97 |
|  |  |  |  |  |  |  |  | Test * Treatment | 1 | 44 | 3.76 | 0.06 |
|  |  |  |  |  |  |  |  | Test * Sex * Treatment | 1 | 44 | 0.59 | 0.45 |
| **Latency to reward** | | |  |  |  |  |  |  |  |  |  |  |
|  |  |  | **Baseline** | | **Test** |  |  | **Factors** | **df** | **df** | **F** | **Sig.** |
| **N** | **Sex** | **Treatment** | **Mean** | **STDV** | **Mean** | **STDV** |  | Test | 1 | 44 | 3.25 | 0.08 |
| 13 | Male | Control | 1.84 | 0.67 | 1.95 | 0.83 |  | Sex | 1 | 44 | 0.86 | 0.36 |
| 12 |  | WMI | 1.96 | 1.06 | 2.04 | 1.30 |  | Treatment | 1 | 44 | 0.27 | 0.60 |
| 15 | Female | Control | 1.72 | 0.48 | 2.07 | 1.35 |  | Sex * Treatment | 1 | 44 | 0.86 | 0.36 |
| 8 |  | WMI | 1.26 | 0.41 | 1.80 | 1.16 |  | Test * Sex | 1 | 44 | 1.41 | 0.24 |
|  |  |  |  |  |  |  |  | Test * Treatment | 1 | 44 | 0.09 | 0.77 |
|  |  |  |  |  |  |  |  | Test * Sex * Treatment | 1 | 44 | 0.14 | 0.72 |

**Post hoc tests**

| **Omissions** | |  |  |  |  |  |  |  |  |  | **Bonferroni adjustment: p<0.025** | | | |
| --- | --- | --- | --- | --- | --- | --- | --- | --- | --- | --- | --- | --- | --- | --- |
|  |  | **Male** | |  |  | **Female** | |  |  |  | **Females** | **t** | **df** | **Sig.** |
| **Factors** |  | **df** | **df** | **F** | **Sig.** | **df** | **df** | **F** | **Sig.** |  | **Baseline: ctrl vs WMI** | -0.31 | 21 | 0.76 |
| *Test* |  | *1* | *23* | *34.11* | *<0.001* | *1* | *21* | *13.25* | *0.002* |  | ***Test: ctrl vs WMI*** | *-2.5* | *21* | *0.02* |
| *Treatment* | | *1* | *23* | *5.69* | *0.03* | 1 | 21 | 4.04 | 0.06 |  |  |  |  |  |
| Test * Treatment | | 1 | 23 | 1.69 | 0.21 | 1 | 21 | 4.53 | 0.05 |  |  |  |  |  |
|  |  |  |  |  |  |  |  |  |  |  |  |  |  |  |
|  |  |  |  |  |  |  |  |  |  |  |  |  |  |  |
|  |  |  |  |  |  |  |  |  |  |  |  |  |  |  |
| **Preserverative responses** | | | |  |  |  |  |  |  |  |  |  |  |  |
|  |  | **Male** | |  |  | **Female** | |  |  |  |  |  |  |  |
| **Factors** |  | **df** | **df** | **F** | **Sig.** | **df** | **df** | **F** | **Sig.** |  |  |  |  |  |
| Test |  | 1 | 23 | 0.06 | 0.80 | 1 | 21 | 0.94 | 0.34 |  |  |  |  |  |
| Treatment | | *1* | *23* | *4.69* | *0.04* | 1 | 21 | 3.25 | 0.09 |  |  |  |  |  |
| Test * Treatment | | 1 | 23 | 0.24 | 0.63 | 1 | 21 | 0.79 | 0.39 |  |  |  |  |  |

**Table 5: Mean variable ITI (3, 5 and 7 sec)**

| **Parameters** | |  |  |  |  |  |  | **Statistics** |  |  |  |  |
| --- | --- | --- | --- | --- | --- | --- | --- | --- | --- | --- | --- | --- |
| **Accuracy** | |  |  |  |  |  |  |  |  |  |  |  |
|  |  |  | **Baseline** | | **Test** |  |  | **Factors** | **df** | **df** | **F** | **Sig.** |
| **N** | **Sex** | **Treatment** | **Mean** | **STDV** | **Mean** | **STDV** |  | Test | 1 | 44 | 0.50 | 0.48 |
| 13 | Male | Control | 93.94 | 4.66 | 95.43 | 2.83 |  | Sex | 1 | 44 | 0.00 | 0.96 |
| 12 |  | WMI | 94.49 | 2.77 | 95.22 | 3.35 |  | Treatment | 1 | 44 | 0.12 | 0.74 |
| 15 | Female | Control | 94.83 | 3.05 | 94.37 | 4.16 |  | Sex * Treatment | 1 | 44 | 0.02 | 0.88 |
| 8 |  | WMI | 95.11 | 3.09 | 94.95 | 3.53 |  | Test * Sex | 1 | 44 | 1.61 | 0.21 |
|  |  |  |  |  |  |  |  | Test * Treatment | 1 | 44 | 0.04 | 0.84 |
|  |  |  |  |  |  |  |  | Test * Sex * Treatment | 1 | 44 | 0.23 | 0.64 |
| **Premature** | |  |  |  |  |  |  |  |  |  |  |  |
|  |  |  | **Baseline** | | **Test** |  |  | **Factors** | **df** | **df** | **F** | **Sig.** |
| **N** | **Sex** | **Treatment** | **Mean** | **STDV** | **Mean** | **STDV** |  | *Test* | *1* | *44* | *31.78* | *<0.001* |
| 13 | Male | Control | 6.85 | 6.84 | 12.23 | 9.17 |  | Sex | 1 | 44 | 2.15 | 0.15 |
| 12 |  | WMI | 4.92 | 4.08 | 11.08 | 7.08 |  | Treatment | 1 | 44 | 0.32 | 0.57 |
| 15 | Female | Control | 3.33 | 2.55 | 6.27 | 6.56 |  | Sex * Treatment | 1 | 44 | 2.39 | 0.13 |
| 8 |  | WMI | 5.63 | 2.62 | 10.63 | 5.07 |  | Test * Sex | 1 | 44 | 1.10 | 0.30 |
|  |  |  |  |  |  |  |  | Test * Treatment | 1 | 44 | 0.68 | 0.41 |
|  |  |  |  |  |  |  |  | Test * Sex * Treatment | 1 | 44 | 0.14 | 0.71 |
| **Omissions** | |  |  |  |  |  |  |  |  |  |  |  |
|  |  |  | **Baseline** | | **Test** |  |  | **Factors** | **df** | **df** | **F** | **Sig.** |
| **N** | **Sex** | **Treatment** | **Mean** | **STDV** | **Mean** | **STDV** |  | Test | 1 | 44 | 1.31 | 0.26 |
| 13 | Male | Control | 8.08 | 4.42 | 8.23 | 4.95 |  | Sex | 1 | 44 | 0.86 | 0.36 |
| 12 |  | WMI | 4.83 | 3.79 | 4.83 | 4.11 |  | Treatment | 1 | 44 | 0.69 | 0.41 |
| 15 | Female | Control | 4.73 | 3.01 | 4.73 | 2.69 |  | *Sex * Treatment* | *1* | *44* | *5.48* | *0.02* |
| 8 |  | WMI | 7.50 | 4.31 | 5.13 | 3.80 |  | Test * Sex | 1 | 44 | 1.70 | 0.20 |
|  |  |  |  |  |  |  |  | Test * Treatment | 1 | 44 | 1.70 | 0.20 |
|  |  |  |  |  |  |  |  | Test * Sex * Treatment | 1 | 44 | 1.31 | 0.26 |
| **Preserverative responses** | | | |  |  |  |  |  |  |  |  |  |
|  |  |  | **Baseline** | | **Test** |  |  | **Factors** | **df** | **df** | **F** | **Sig.** |
| **N** | **Sex** | **Treatment** | **Mean** | **STDV** | **Mean** | **STDV** |  | Test | 1 | 44 | 0.70 | 0.41 |
| 13 | Male | Control | 6.85 | 4.20 | 6.23 | 3.75 |  | *Sex* | *1* | *44* | *4.02* | *0.05* |
| 12 |  | WMI | 4.42 | 3.42 | 4.25 | 2.05 |  | Treatment | 1 | 44 | 3.19 | 0.08 |
| 15 | Female | Control | 12.33 | 10.95 | 10.20 | 9.03 |  | Sex * Treatment | 1 | 44 | 0.34 | 0.56 |
| 8 |  | WMI | 6.63 | 5.01 | 7.25 | 6.14 |  | Test * Sex | 1 | 44 | 0.07 | 0.79 |
|  |  |  |  |  |  |  |  | Test * Treatment | 1 | 44 | 1.38 | 0.25 |
|  |  |  |  |  |  |  |  | Test * Sex * Treatment | 1 | 44 | 0.71 | 0.40 |

| **Parameters** | |  |  |  |  |  |  | **Statistics** |  |  |  |  |
| --- | --- | --- | --- | --- | --- | --- | --- | --- | --- | --- | --- | --- |
| **Latency to correct** | | |  |  |  |  |  |  |  |  |  |  |
|  |  |  | **Baseline** | | **Test** |  |  | **Factors** | **df** | **df** | **F** | **Sig.** |
| **N** | **Sex** | **Treatment** | **Mean** | **STDV** | **Mean** | **STDV** |  | *Test* | *1* | *44* | *5.42* | *0.03* |
| 13 | Male | Control | 0.62 | 0.06 | 0.63 | 0.08 |  | Sex | 1 | 44 | 0.85 | 0.36 |
| 12 |  | WMI | 0.60 | 0.12 | 0.63 | 0.15 |  | Treatment | 1 | 44 | 0.97 | 0.33 |
| 15 | Female | Control | 0.62 | 0.07 | 0.62 | 0.09 |  | Sex * Treatment | 1 | 44 | 0.34 | 0.57 |
| 8 |  | WMI | 0.56 | 0.09 | 0.59 | 0.05 |  | Test * Sex | 1 | 44 | 0.01 | 0.93 |
|  |  |  |  |  |  |  |  | Test * Treatment | 1 | 44 | 2.66 | 0.11 |
|  |  |  |  |  |  |  |  | Test * Sex * Treatment | 1 | 44 | 0.09 | 0.77 |
| **Latency to reward** | | |  |  |  |  |  |  |  |  |  |  |
|  |  |  | **Baseline** | | **Test** |  |  | **Factors** | **df** | **df** | **F** | **Sig.** |
| **N** | **Sex** | **Treatment** | **Mean** | **STDV** | **Mean** | **STDV** |  | Test | 1 | 44 | 0.31 | 0.58 |
| 13 | Male | Control | 2.03 | 0.87 | 1.99 | 0.76 |  | *Sex* | *1* | *44* | *8.77* | *0.01* |
| 12 |  | WMI | 1.59 | 0.46 | 1.59 | 0.48 |  | *Treatment* | *1* | *44* | *4.74* | *0.04* |
| 15 | Female | Control | 1.50 | 0.41 | 1.43 | 0.52 |  | Sex * Treatment | 1 | 44 | 0.26 | 0.62 |
| 8 |  | WMI | 1.22 | 0.35 | 1.19 | 0.25 |  | Test * Sex | 1 | 44 | 0.06 | 0.81 |
|  |  |  |  |  |  |  |  | Test * Treatment | 1 | 44 | 0.13 | 0.72 |
|  |  |  |  |  |  |  |  | Test * Sex * Treatment | 1 | 44 | 0.00 | 0.95 |

**Post hoc tests**

| **Omissions** | |  |  |  |  |  |  |  |  |
| --- | --- | --- | --- | --- | --- | --- | --- | --- | --- |
|  |  | **Male** | |  |  | **Female** | |  |  |
| **Factors** |  | **df** | **df** | **F** | **Sig.** | **df** | **df** | **F** | **Sig.** |
| Test |  | 1 | 23 | 0.01 | 0.92 | 1 | 21 | 3.55 | 0.07 |
| *Treatment* | | *1* | *23* | *4.37* | *0.05* | 1 | 21 | 1.47 | 0.24 |
| Test * Treatment | | 1 | 23 | 0.01 | 0.92 | 1 | 21 | 3.55 | 0.07 |

| **Preserverative responses** | | | |  |  |  |  |  |  |
| --- | --- | --- | --- | --- | --- | --- | --- | --- | --- |
|  |  | **Male** | |  |  | **Female** | |  |  |
| **Factors** |  | **df** | **df** | **F** | **Sig.** | **df** | **df** | **F** | **Sig.** |
| Test |  | 1 | 23 | 0.38 | 0.54 | 1 | 21 | 0.35 | 0.56 |
| Treatment | | 1 | 23 | 3.17 | 0.09 | 1 | 21 | 1.41 | 0.25 |
| Test * Treatment | | 1 | 23 | 0.13 | 0.73 | 1 | 21 | 1.18 | 0.29 |

| **Latency to reward** | |  |  |  |  |  |  |  |  |
| --- | --- | --- | --- | --- | --- | --- | --- | --- | --- |
|  |  | **Male** | |  |  | **Female** | |  |  |
| **Factors** |  | **df** | **df** | **F** | **Sig.** | **df** | **df** | **F** | **Sig.** |
| Test |  | 1 | 23 | 0.04 | 0.84 | 1 | 21 | 0.42 | 0.53 |
| Treatment | | 1 | 23 | 2.76 | 0.11 | 1 | 21 | 2.42 | 0.14 |
| Test * Treatment | | 1 | 23 | 0.08 | 0.78 | 1 | 21 | 0.06 | 0.81 |

**Table 6: 7 seconds (long) ITI**

| **Parameters** | |  |  |  |  |  |  | **Statistics** |  |  |  |  |
| --- | --- | --- | --- | --- | --- | --- | --- | --- | --- | --- | --- | --- |
| **Accurary** | |  |  |  |  |  |  |  |  |  |  |  |
|  |  |  | **Baseline** | | **Test** |  |  | **Factors** | **df** | **df** | **F** | **Sig.** |
| **N** | **Sex** | **Treatment** | **Mean** | **STDV** | **Mean** | **STDV** |  | *Test* | *1* | *44* | *15.72* | *<0.001* |
| 13 | Male | Control | 92.74 | 3.53 | 89.27 | 6.62 |  | Sex | 1 | 44 | 0.00 | 0.95 |
| 12 |  | WMI | 94.67 | 4.06 | 93.44 | 3.79 |  | Treatment | 1 | 44 | 1.27 | 0.27 |
| 15 | Female | Control | 93.80 | 4.65 | 91.19 | 6.05 |  | Test * Sex | 1 | 44 | 0.38 | 0.54 |
| 8 |  | WMI | 94.31 | 3.24 | 90.51 | 6.78 |  | Test * Treatment | 1 | 44 | 0.14 | 0.71 |
|  |  |  |  |  |  |  |  | Sex * Treatment | 1 | 44 | 1.41 | 0.24 |
|  |  |  |  |  |  |  |  | Test * Sex * Treatment | 1 | 44 | 1.50 | 0.23 |
| **Premature responses** | | |  |  |  |  |  |  |  |  |  |  |
|  |  |  | **Baseline** | | **Test** |  |  | **Factors** | **df** | **df** | **F** | **Sig.** |
| **N** | **Sex** | **Treatment** | **Mean** | **STDV** | **Mean** | **STDV** |  | *Test* | *1* | *44* | *81.51* | *<0.001* |
| 13 | Male | Control | 5.69 | 5.91 | 24.69 | 15.45 |  | Sex | 1 | 44 | 1.31 | 0.26 |
| 12 |  | WMI | 4.42 | 3.48 | 19.67 | 5.90 |  | Treatment | 1 | 44 | 0.20 | 0.66 |
| 15 | Female | Control | 5.27 | 6.58 | 15.87 | 12.27 |  | Sex * Treatment | 1 | 44 | 1.07 | 0.31 |
| 8 |  | WMI | 2.88 | 3.52 | 20.75 | 11.44 |  | Test * Sex | 1 | 44 | 0.69 | 0.41 |
|  |  |  |  |  |  |  |  | Test * Treatment | 1 | 44 | 0.26 | 0.61 |
|  |  |  |  |  |  |  |  | Test * Sex * Treatment | 1 | 44 | 2.52 | 0.12 |
| **Omissions** | |  |  |  |  |  |  |  |  |  |  |  |
|  |  |  | **Baseline** | | **Test** |  |  | **Factors** | **df** | **df** | **F** | **Sig.** |
| **N** | **Sex** | **Treatment** | **Mean** | **STDV** | **Mean** | **STDV** |  | Test | 1 | 44 | 0.14 | 0.72 |
| 13 | Male | Control | 8.54 | 4.58 | 9.85 | 4.30 |  | Sex | 1 | 44 | 0.07 | 0.80 |
| 12 |  | WMI | 7.58 | 4.46 | 7.58 | 3.85 |  | Treatment | 1 | 44 | 3.01 | 0.09 |
| 15 | Female | Control | 10.87 | 8.82 | 8.00 | 7.17 |  | Sex * Treatment | 1 | 44 | 0.21 | 0.65 |
| 8 |  | WMI | 6.75 | 4.62 | 6.63 | 3.20 |  | Test * Sex | 1 | 44 | 0.88 | 0.35 |
|  |  |  |  |  |  |  |  | Test * Treatment | 1 | 44 | 0.10 | 0.76 |
|  |  |  |  |  |  |  |  | Test * Sex * Treatment | 1 | 44 | 0.78 | 0.38 |
| **Preseverative responses** | | |  |  |  |  |  |  |  |  |  |  |
|  |  |  | **Baseline** | | **Test** |  |  | **Factors** | **df** | **df** | **F** | **Sig.** |
| **N** | **Sex** | **Treatment** | **Mean** | **STDV** | **Mean** | **STDV** |  | Test | 1 | 44 | 0.05 | 0.82 |
| 13 | Male | Control | 4.08 | 4.05 | 3.46 | 2.90 |  | *Sex* | *1* | *44* | *11.23* | *<0.001* |
| 12 |  | WMI | 3.25 | 2.30 | 3.58 | 1.88 |  | Treatment | 1 | 44 | 3.10 | 0.09 |
| 15 | Female | Control | 12.53 | 12.17 | 11.07 | 8.52 |  | Sex * Treatment | 1 | 44 | 2.39 | 0.13 |
| 8 |  | WMI | 5.00 | 3.21 | 7.75 | 7.44 |  | Test * Sex | 1 | 44 | 0.12 | 0.73 |
|  |  |  |  |  |  |  |  | Test * Treatment | 1 | 44 | 1.33 | 0.26 |
|  |  |  |  |  |  |  |  | Test * Sex * Treatment | 1 | 44 | 0.53 | 0.47 |

| **Parameters** | |  |  |  |  |  |  | **Statistics** |  |  |  |  |
| --- | --- | --- | --- | --- | --- | --- | --- | --- | --- | --- | --- | --- |
| **Latency to correct** | | |  |  |  |  |  |  |  |  |  |  |
|  |  |  | **Baseline** | | **Test** |  |  | **Factors** | **df** | **df** | **F** | **Sig.** |
| **N** | **Sex** | **Treatment** | **Mean** | **STDV** | **Mean** | **STDV** |  | Test | 1 | 44 | 2.31 | 0.14 |
| 13 | Male | Control | 0.65 | 0.08 | 0.65 | 0.06 |  | Sex | 1 | 44 | 1.58 | 0.22 |
| 12 |  | WMI | 0.64 | 0.11 | 0.65 | 0.16 |  | Treatment | 1 | 44 | 0.72 | 0.40 |
| 15 | Female | Control | 0.65 | 0.07 | 0.63 | 0.09 |  | Sex * Treatment | 1 | 44 | 0.60 | 0.45 |
| 8 |  | WMI | 0.61 | 0.09 | 0.57 | 0.08 |  | Test * Sex | 1 | 44 | 2.69 | 0.11 |
|  |  |  |  |  |  |  |  | Test * Treatment | 1 | 44 | 0.01 | 0.94 |
|  |  |  |  |  |  |  |  | Test * Sex * Treatment | 1 | 44 | 0.87 | 0.36 |
| **Latency to reward** | | |  |  |  |  |  |  |  |  |  |  |
|  |  |  | **Baseline** | | **Test** |  |  | **Factors** | **df** | **df** | **F** | **Sig.** |
| **N** | **Sex** | **Treatment** | **Mean** | **STDV** | **Mean** | **STDV** |  | Test | 1 | 44 | 0.13 | 0.72 |
| 13 | Male | Control | 1.77 | 0.49 | 1.75 | 0.37 |  | *Sex* | *1* | *44* | *6.31* | *0.02* |
| 12 |  | WMI | 1.51 | 0.36 | 1.68 | 0.56 |  | Treatment | 1 | 44 | 1.07 | 0.31 |
| 15 | Female | Control | 3.55 | 3.30 | 2.44 | 1.80 |  | Sex * Treatment | 1 | 44 | 0.39 | 0.54 |
| 8 |  | WMI | 1.63 | 0.72 | 3.05 | 2.63 |  | Test * Sex | 1 | 44 | 0.01 | 0.91 |
|  |  |  |  |  |  |  |  | *Test * Treatment* | *1* | *44* | *4.40* | *0.04* |
|  |  |  |  |  |  |  |  | Test * Sex * Treatment | 1 | 44 | 3.29 | 0.08 |

**Post hoc tests**

| **Preseverative responses** | | | | |  | |  | |  | |  | |  | |  | |
| --- | --- | --- | --- | --- | --- | --- | --- | --- | --- | --- | --- | --- | --- | --- | --- | --- |
|  |  | **male** | | |  | |  | | **female** | | | |  | |  | |
| **Factors** |  | **df** | | **df** | | **F** | | **Sig.** | | **df** | | **df** | | **F** | | **Sig.** |
| Test | | 1 | | 23 | | 0.04 | | 0.85 | | 1 | | 21 | | 0.08 | | 0.78 |
| Treatment | | 1 | | 23 | | 0.14 | | 0.71 | | 1 | | 21 | | 2.64 | | 0.12 |
| Test * Treatment | | 1 | | 23 | | 0.44 | | 0.52 | | 1 | | 21 | | 0.88 | | 0.36 |
|  |  |  | |  | |  | |  | |  | |  | |  | |  |
| **Latency to reward** | | |  | |  | |  | |  | |  | |  | |  | |
|  |  | **male** | | |  | |  | | **female** | | | |  | |  | |
| **Factors** |  | **df** | | **df** | | **F** | | **Sig.** | | **df** | |  | | **F** | | **Sig.** |
| Test | | 1 | | 23 | | 0.92 | | 0.35 | | 1 | | 21 | | 0.05 | | 0.82 |
| Treatment | | 1 | | 23 | | 1.02 | | 0.32 | | 1 | | 21 | | 0.63 | | 0.44 |
| Test * Treatment | | 1 | | 23 | | 1.20 | | 0.29 | | 1 | | 21 | | 3.42 | | 0.08 |

**Table 7: Variable long ITI (7, 9, 11 and 13 seconds)**

| **Descriptive Statistics** | | | | | | | | |  |  |  |  |
| --- | --- | --- | --- | --- | --- | --- | --- | --- | --- | --- | --- | --- |
| **Accurary** | |  |  |  |  |  |  |  |  |  |  |  |
|  |  |  | **Baseline** | | **Test** |  |  | **Factors** | **df** | **df** | **F** | **Sig.** |
| **N** | **Sex** | **Treatment** | **Mean** | **STDV** | **Mean** | **STDV** |  | *Test* | *1* | *44* | *15.90* | *<0.001* |
| 13 | Male | Control | 94.25 | 3.35 | 91.92 | 5.66 |  | Sex | 1 | 44 | 1.35 | 0.25 |
| 12 |  | WMI | 96.05 | 3.12 | 94.60 | 4.76 |  | Treatment | 1 | 44 | 1.41 | 0.24 |
| 15 | Female | Control | 94.94 | 3.25 | 90.47 | 7.38 |  | Sex * Treatment | 1 | 44 | 0.68 | 0.41 |
| 8 |  | WMI | 95.34 | 2.24 | 90.88 | 2.98 |  | Test * Sex | 1 | 44 | 2.61 | 0.11 |
|  |  |  |  |  |  |  |  | Test * Treatment | 1 | 44 | 0.08 | 0.78 |
|  |  |  |  |  |  |  |  | Test * Sex * Treatment | 1 | 44 | 0.07 | 0.79 |
| **Premature** | |  |  |  |  |  |  |  |  |  |  |  |
|  |  |  | **Baseline** | | **Test** |  |  | **Factors** | **df** | **df** | **F** | **Sig.** |
| **N** | **Sex** | **Treatment** | **Mean** | **STDV** | **Mean** | **STDV** |  | *Test* | *1* | *44* | *276.32* | *<0.001* |
| 13 | Male | Control | 5.54 | 5.38 | 52.85 | 22.53 |  | Sex | 1 | 44 | 0.06 | 0.82 |
| 12 |  | WMI | 2.67 | 2.53 | 49.17 | 20.63 |  | Treatment | 1 | 44 | 0.63 | 0.43 |
| 15 | Female | Control | 3.73 | 3.73 | 41.47 | 17.65 |  | Sex * Treatment | 1 | 44 | 3.29 | 0.08 |
| 8 |  | WMI | 4.88 | 3.27 | 57.13 | 17.67 |  | Test * Sex | 1 | 44 | 0.12 | 0.73 |
|  |  |  |  |  |  |  |  | Test * Treatment | 1 | 44 | 1.54 | 0.22 |
|  |  |  |  |  |  |  |  | Test * Sex * Treatment | 1 | 44 | 1.92 | 0.17 |
| **Omissions** | |  |  |  |  |  |  |  |  |  |  |  |
|  |  |  | **Baseline** | | **Test** |  |  | **Factors** | **df** | **df** | **F** | **Sig.** |
| **N** | **Sex** | **Treatment** | **Mean** | **STDV** | **Mean** | **STDV** |  | *Test* | *1* | *44* | *18.92* | *<0.001* |
| 13 | Male | Control | 5.08 | 3.40 | 10.00 | 5.77 |  | Sex | 1 | 44 | 0.33 | 0.57 |
| 12 |  | WMI | 4.08 | 3.58 | 7.67 | 5.47 |  | Treatment | 1 | 44 | 2.42 | 0.13 |
| 15 | Female | Control | 4.73 | 3.47 | 9.13 | 5.80 |  | Sex * Treatment | 1 | 44 | 0.00 | 0.99 |
| 8 |  | WMI | 5.00 | 3.02 | 5.50 | 2.27 |  | Test * Sex | 1 | 44 | 1.37 | 0.25 |
|  |  |  |  |  |  |  |  | Test * Treatment | 1 | 44 | 2.89 | 0.10 |
|  |  |  |  |  |  |  |  | Test * Sex * Treatment | 1 | 44 | 0.69 | 0.41 |
| **Preseverative responses** | | |  |  |  |  |  |  |  |  |  |  |
|  |  |  | **Baseline** | | **Test** |  |  | **Factors** | **df** | **df** | **F** | **Sig.** |
| **N** | **Sex** | **Treatment** | **Mean** | **STDV** | **Mean** | **STDV** |  | *Test* | *1* | *44* | *18.30* | *<0.001* |
| 13 | Male | Control | 4.62 | 2.81 | 2.15 | 2.15 |  | Sex | 1 | 44 | 2.61 | 0.11 |
| 12 |  | WMI | 2.67 | 2.39 | 2.67 | 2.42 |  | Treatment | 1 | 44 | 3.48 | 0.07 |
| 15 | Female | Control | 9.20 | 9.73 | 5.13 | 5.05 |  | Sex * Treatment | 1 | 44 | 1.71 | 0.20 |
| 8 |  | WMI | 4.75 | 2.19 | 1.38 | 1.30 |  | *Test * Sex* | *1* | *44* | *4.63* | *0.04* |
|  |  |  |  |  |  |  |  | Test * Treatment | 1 | 44 | 1.86 | 0.18 |
|  |  |  |  |  |  |  |  | Test * Sex * Treatment | 1 | 44 | 0.59 | 0.45 |

|  |  |  |  |  |  |  | **Statistics** |  |  |  |  |
| --- | --- | --- | --- | --- | --- | --- | --- | --- | --- | --- | --- |
| **Latency  to  correct** | | | | | | | | | | | |
|  |  | **Baseline** | | **Test** |  |  | **Factors** | **df** | **df** | **F** | **Sig.** |
| **Sex** | **Treatment** | **Mean** | **STDV** | **Mean** | **STDV** |  | *Test* | *1* | *44* | *38.70* | *<0.001* |
| Male | Control | 0.60 | 0.08 | 0.69 | 0.07 |  | Sex | 1 | 44 | 1.81 | 0.19 |
|  | WMI | 0.60 | 0.12 | 0.70 | 0.12 |  | Treatment | 1 | 44 | 1.01 | 0.32 |
| Female | Control | 0.62 | 0.09 | 0.66 | 0.07 |  | Sex * Treatment | 1 | 44 | 1.69 | 0.20 |
|  | WMI | 0.57 | 0.07 | 0.60 | 0.08 |  | *Test * Sex* | *1* | *44* | *9.82* | *0.003* |
|  |  |  |  |  |  |  | Test * Treatment | 1 | 44 | 0.00 | 0.97 |
|  |  |  |  |  |  |  | Test * Sex * Treatment | 1 | 44 | 0.14 | 0.71 |
| **Latency  to  reward** | | | | | | | | | | | |
|  |  | **Baseline** | | **Test** |  |  | **Factors** | **df** | **df** | **F** | **Sig.** |
| **Sex** | **Treatment** | **Mean** | **STDV** | **Mean** | **STDV** |  | Test | 1 | 44 | 0.24 | 0.63 |
| Male | Control | 1.77 | 0.61 | 1.68 | 0.60 |  | Sex | 1 | 44 | 3.05 | 0.09 |
|  | WMI | 1.46 | 0.36 | 1.26 | 0.29 |  | *Treatment* | *1* | *44* | *10.61* | *0.002* |
| Female | Control | 1.48 | 0.40 | 1.60 | 0.52 |  | Sex * Treatment | 1 | 44 | 0.04 | 0.84 |
|  | WMI | 1.11 | 0.25 | 1.15 | 0.29 |  | Test * Sex | 1 | 44 | 3.42 | 0.07 |
|  |  |  |  |  |  |  | Test * Treatment | 1 | 44 | 0.57 | 0.46 |
|  |  |  |  |  |  |  | Test * Sex * Treatment | 1 | 44 | 0.01 | 0.94 |

**Post hoc tests**

| **Preseverative responses** | | | |  |  |  |  |  |  |  | **Preseverative responses** |  |  |  |  |
| --- | --- | --- | --- | --- | --- | --- | --- | --- | --- | --- | --- | --- | --- | --- | --- |
|  |  | **Male** | |  |  | **Female** | | |  |  |  | **df** | **df** | **F** | **Sig.** |
| **Factors** |  | **df** | **df** | **F** | **Sig.** | **df** | **df** | **F** | **Sig.** |  | **Males baseline: ctrl vs WMI** | 1 | 23 | 3.46 | 0.08 |
| *Test* |  | *1* | *23* | *7.55* | *0.01* | *1* | *21* | *10.94* | *0.003* |  | **Males test: ctrl vs WMI** | 1 | 23 | 0.31 | 0.58 |
| Treatment | | 1 | 23 | 0.67 | 0.42 | 1 | 21 | 2.54 | 0.13 |  |  |  |  |  |  |
| Test * Treatment | | *1* | *23* | *7.55* | *0.01* | 1 | 21 | 0.09 | 0.76 |  |  |  |  |  |  |

| **Latency to correct** | |  |  |  |  |  |  |  |  |
| --- | --- | --- | --- | --- | --- | --- | --- | --- | --- |
|  |  | **Male** | |  |  | **Female** | | |  |
| **Factors** |  | **df** | **df** | **F** | **Sig.** | **df** | **df** | **F** | **Sig.** |
| *Test* |  | *1* | *23* | *68.49* | *<0.001* | 1 | 21 | 3.30 | 0.08 |
| Treatment | | 1 | 23 | 0.04 | 0.85 | 1 | 21 | 3.59 | 0.07 |
| Test * Treatment | | 1 | 23 | 0.13 | 0.72 | 1 | 21 | 0.04 | 0.84 |

| **Latency to reward** | |  |  |  |  |  |  |  |  |
| --- | --- | --- | --- | --- | --- | --- | --- | --- | --- |
|  |  | **Ctrl** | |  |  | **WMI** | |  |  |
| **Factors** |  | **df** | **df** | **F** | **Sig.** | **df** | **df** | **F** | **Sig.** |
| *Test* |  | *1* | *26* | *68.49* | *<0.001* | 1 | 18 | 0.03 | 0.87 |
| Sex |  | 1 | 26 | 0.04 | 0.85 | 1 | 18 | 1.08 | 0.31 |
| Test * Sex | | 1 | 26 | 0.13 | 0.72 | 1 | 18 | 1.27 | 0.27 |

**Table 8: 0.5 sec (short) stimulus duration**

| **Parameters** | |  |  |  |  |  |  | **Statistics** |  |  |  |  |
| --- | --- | --- | --- | --- | --- | --- | --- | --- | --- | --- | --- | --- |
| **Accurary** | |  |  |  |  |  |  |  |  |  |  |  |
|  |  |  | **Baseline** | | **Test** |  |  | **Factors** | **df** | **df** | **F** | **Sig.** |
| **N** | **Sex** | **Treatment** | **Mean** | **STDV** | **Mean** | **STDV** |  | *Test* | *1* | *44* | *102.86* | *<0.001* |
| 13 | Male | Control | 92.03 | 5.95 | 82.21 | 8.29 |  | Sex | 1 | 44 | 0.15 | 0.70 |
| 12 |  | WMI | 94.36 | 3.60 | 85.02 | 7.12 |  | Treatment | 1 | 44 | 0.36 | 0.55 |
| 15 | Female | Control | 95.16 | 3.67 | 83.59 | 7.84 |  | Sex * Treatment | 1 | 44 | 1.23 | 0.27 |
| 8 |  | WMI | 93.18 | 3.38 | 84.05 | 4.63 |  | Test * Sex | 1 | 44 | 0.15 | 0.70 |
|  |  |  |  |  |  |  |  | Test * Treatment | 1 | 44 | 0.56 | 0.46 |
|  |  |  |  |  |  |  |  | Test * Sex * Treatment | 1 | 44 | 0.25 | 0.62 |
| **Premature** | |  |  |  |  |  |  |  |  |  |  |  |
|  |  |  | **Baseline** | | **Test** |  |  | **Factors** | **df** | **df** | **F** | **Sig.** |
| **N** | **Sex** | **Treatment** | **Mean** | **STDV** | **Mean** | **STDV** |  | *Test* | *1* | *44* | *22.82* | *<0.001* |
| 13 | Male | Control | 4.23 | 2.35 | 9.62 | 7.87 |  | Sex | 1 | 44 | 1.05 | 0.31 |
| 12 |  | WMI | 4.33 | 3.85 | 7.25 | 5.55 |  | Treatment | 1 | 44 | 1.51 | 0.23 |
| 15 | Female | Control | 3.73 | 2.34 | 8.33 | 6.69 |  | Sex * Treatment | 1 | 44 | 0.08 | 0.79 |
| 8 |  | WMI | 1.88 | 1.46 | 6.63 | 5.76 |  | Test * Sex | 1 | 44 | 0.08 | 0.78 |
|  |  |  |  |  |  |  |  | Test * Treatment | 1 | 44 | 0.39 | 0.53 |
|  |  |  |  |  |  |  |  | Test * Sex * Treatment | 1 | 44 | 0.50 | 0.48 |
| **Omissions** | |  |  |  |  |  |  |  |  |  |  |  |
|  |  |  | **Baseline** | | **Test** |  |  | **Factors** | **df** | **df** | **F** | **Sig.** |
| **N** | **Sex** | **Treatment** | **Mean** | **STDV** | **Mean** | **STDV** |  | *Test* | *1* | *44* | *88.77* | *<0.001* |
| 13 | Male | Control | 10.23 | 6.04 | 20.92 | 10.10 |  | Sex | 1 | 44 | 0.01 | 0.95 |
| 12 |  | WMI | 6.75 | 4.71 | 13.75 | 5.51 |  | Treatment | 1 | 44 | 0.55 | 0.46 |
| 15 | Female | Control | 7.07 | 4.80 | 16.33 | 9.49 |  | *Sex * Treatment* | *1* | *44* | *4.98* | *0.03* |
| 8 |  | WMI | 7.75 | 5.18 | 21.00 | 6.99 |  | Test * Sex | 1 | 44 | 1.28 | 0.26 |
|  |  |  |  |  |  |  |  | Test * Treatment | 1 | 44 | 0.01 | 0.95 |
|  |  |  |  |  |  |  |  | Test * Sex * Treatment | 1 | 44 | 3.24 | 0.08 |
| **Perserverant responses** | | |  |  |  |  |  |  |  |  |  |  |
|  |  |  | **Baseline** | | **Test** |  |  | **Factors** | **df** | **df** | **F** | **Sig.** |
| **N** | **Sex** | **Treatment** | **Mean** | **STDV** | **Mean** | **STDV** |  | Test | 1 | 44 | 2.91 | 0.10 |
| 13 | Male | Control | 4.54 | 3.80 | 3.23 | 4.60 |  | *Sex* | *1* | *44* | *4.41* | *0.04* |
| 12 |  | WMI | 2.58 | 2.11 | 2.50 | 2.28 |  | Treatment | 1 | 44 | 3.24 | 0.08 |
| 15 | Female | Control | 8.33 | 7.20 | 6.27 | 6.71 |  | Sex * Treatment | 1 | 44 | 0.49 | 0.49 |
| 8 |  | WMI | 5.25 | 4.74 | 3.25 | 3.45 |  | Test * Sex | 1 | 44 | 0.70 | 0.41 |
|  |  |  |  |  |  |  |  | Test * Treatment | 1 | 44 | 0.16 | 0.69 |
|  |  |  |  |  |  |  |  | Test * Sex * Treatment | 1 | 44 | 0.13 | 0.72 |

| **Parameters** | |  |  |  |  |  |  | **Statistics** |  |  |  |  |
| --- | --- | --- | --- | --- | --- | --- | --- | --- | --- | --- | --- | --- |
| **Latency to correct** | | |  |  |  |  |  |  |  |  |  |  |
|  |  |  | **Baseline** | | **Test** |  |  | **Factors** | **df** | **df** | **F** | **Sig.** |
| **N** | **Sex** | **Treatment** | **Mean** | **STDV** | **Mean** | **STDV** |  | *Test* | *1* | *44* | *66.66* | *<0.001* |
| 13 | Male | Control | 0.65 | 0.05 | 0.57 | 0.06 |  | Sex | 1 | 44 | 1.42 | 0.24 |
| 12 |  | WMI | 0.65 | 0.15 | 0.57 | 0.11 |  | Treatment | 1 | 44 | 1.05 | 0.31 |
| 15 | Female | Control | 0.65 | 0.08 | 0.57 | 0.07 |  | Sex * Treatment | 1 | 44 | 1.07 | 0.31 |
| 8 |  | WMI | 0.59 | 0.06 | 0.52 | 0.09 |  | Test * Sex | 1 | 44 | 0.16 | 0.69 |
|  |  |  |  |  |  |  |  | Test * Treatment | 1 | 44 | 0.05 | 0.83 |
|  |  |  |  |  |  |  |  | Test * Sex * Treatment | 1 | 44 | 0.08 | 0.78 |
| **Latency to reward** | | |  |  |  |  |  |  |  |  |  |  |
|  |  |  | **Baseline** | | **Test** |  |  | **Factors** | **df** | **df** | **F** | **Sig.** |
| **N** | **Sex** | **Treatment** | **Mean** | **STDV** | **Mean** | **STDV** |  | Test | 1 | 44 | 0.43 | 0.52 |
| 13 | Male | Control | 1.82 | 0.60 | 1.63 | 0.48 |  | Sex | 1 | 44 | 0.00 | 0.96 |
| 12 |  | WMI | 1.71 | 0.65 | 1.40 | 0.30 |  | *Treatment* | *1* | *44* | *6.09* | *0.02* |
| 15 | Female | Control | 1.97 | 1.16 | 1.90 | 0.91 |  | Sex * Treatment | 1 | 44 | 1.85 | 0.18 |
| 8 |  | WMI | 1.23 | 0.38 | 1.43 | 0.40 |  | Test * Sex | 1 | 44 | 1.19 | 0.28 |
|  |  |  |  |  |  |  |  | Test * Treatment | 1 | 44 | 0.09 | 0.77 |
|  |  |  |  |  |  |  |  | Test * Sex * Treatment | 1 | 44 | 0.47 | 0.50 |

**Post hoc tests:**

| **Omissions** | |  |  |  |  |  |  |  |  |
| --- | --- | --- | --- | --- | --- | --- | --- | --- | --- |
|  |  | **Male** | |  |  | **Female** | |  |  |
| **Factors** |  | **df** |  | **F** | **Sig.** | **df** |  | **F** | **Sig.** |
| *Test* |  | *1* | *23* | *47.45* | *<0.001* | *1* | *21* | *41.75* | *<0.001* |
| *Treatment* | | *1* | *23* | *4.61* | *0.04* | 1 | 21 | 1.08 | 0.31 |
| Test * Treatment | | 1 | 23 | 2.07 | 0.16 | 1 | 21 | 1.31 | 0.27 |
|  |  |  |  |  |  |  |  |  |  |
| **Perserverant responses** | | |  |  |  |  |  |  |  |
|  |  | **Male** | |  |  | **Female** | |  |  |
| **Factors** |  | **df** |  | **F** | **Sig.** | **df** |  | **F** | **Sig.** |
| Test |  | 1 | 23 | 1.96 | 0.17 | 1 | 21 | 1.59 | 0.22 |
| Treatment | | 1 | 23 | 1.12 | 0.30 | 1 | 21 | 1.98 | 0.17 |
| Test * Treatment | | 1 | 23 | 1.52 | 0.23 | 1 | 21 | <0.001 | 0.98 |

| **Latency to reward** | |  |  |  |  |  |  |  |  |
| --- | --- | --- | --- | --- | --- | --- | --- | --- | --- |
|  |  | **Control** | |  |  | **WMI** | |  |  |
| **Factors** |  | **df** |  | **F** | **Sig.** | **df** |  | **F** | **Sig.** |
| Test |  | 1 | 26 | 0.36 | 0.56 | 1 | 18 | 0.25 | 0.63 |
| Sex |  | 1 | 26 | 0.81 | 0.38 | 1 | 18 | 1.45 | 0.25 |
| Test * Sex | | 1 | 26 | 0.06 | 0.80 | *1* | *18* | *6.12* | *0.02* |

| **Latency to reward** | | **Bonferroni correction p<0.0125** | | |
| --- | --- | --- | --- | --- |
| **WMI** |  | **t** | **df** | **Sig.** |
| **Baseline: m vs f** | | 1.86 | 18 | 0.08 |
| **Test: m vs f** |  | -0.19 | 18 | 0.85 |
| **Male: baseline vs test** | | 2.35 | 11 | 0.04 |
| **Female: baseline vs test** | | -1.26 | 7 | 0.25 |

**Table 9: variable short stimulus duration (alternating 1.2 and 0.5 sec)**

| **Parameters** | |  |  |  |  |  |  | **Statistics** |  |  |  |  |
| --- | --- | --- | --- | --- | --- | --- | --- | --- | --- | --- | --- | --- |
| **Accuracy** |  |  |  |  |  |  |  |  |  |  |  |  |
| **Stim. dur.** | **Trial** | **Sex** | **Treatment** | **Mean** | **STDV** | **N** |  | **Factors** | **df** | **df** | **F** | **Sig.** |
| **1.2 sec** | 0-20 | Male | Control | 95.00 | 5.63 | 13 |  | *Test* | *1* | *44* | *8.17* | *<0.001* |
|  |  |  | WMI | 93.25 | 8.66 | 12 |  | Sex | 1 | 44 | 3.74 | 0.06 |
|  |  | Female | Control | 96.80 | 3.91 | 15 |  | Treatment | 1 | 44 | 0.05 | 0.82 |
|  |  |  | WMI | 94.13 | 4.32 | 8 |  | Sex * Treatment | 1 | 44 | 0.07 | 0.80 |
| **0.5 sec** | 20-40 | Male | Control | 84.54 | 9.52 | 13 |  | *Test * Sex* | *1* | *44* | *2.45* | *0.05* |
|  |  |  | WMI | 86.42 | 7.76 | 12 |  | Test * Treatment | 1 | 44 | 1.32 | 0.27 |
|  |  | Female | Control | 90.47 | 10.08 | 15 |  | Test * Sex * Treatment | 1 | 44 | 0.58 | 0.68 |
|  |  |  | WMI | 92.63 | 7.33 | 8 |  |  |  |  |  |  |
| **1.2 sec** | 40-60 | Male | Control | 93.08 | 6.84 | 13 |  |  |  |  |  |  |
|  |  |  | WMI | 91.00 | 6.30 | 12 |  |  |  |  |  |  |
|  |  | Female | Control | 96.87 | 4.63 | 15 |  |  |  |  |  |  |
|  |  |  | WMI | 93.75 | 5.18 | 8 |  |  |  |  |  |  |
| **0.5 sec** | 60-80 | Male | Control | 86.23 | 9.88 | 13 |  |  |  |  |  |  |
|  |  |  | WMI | 84.33 | 11.94 | 12 |  |  |  |  |  |  |
|  |  | Female | Control | 88.13 | 10.94 | 15 |  |  |  |  |  |  |
|  |  |  | WMI | 92.88 | 7.41 | 8 |  |  |  |  |  |  |
| **1.2 sec** | 80-100 | Male | Control | 92.54 | 7.92 | 13 |  |  |  |  |  |  |
|  |  |  | WMI | 96.17 | 4.37 | 12 |  |  |  |  |  |  |
|  |  | Female | Control | 90.67 | 11.51 | 15 |  |  |  |  |  |  |
|  |  |  | WMI | 92.88 | 6.66 | 8 |  |  |  |  |  |  |

**Post hoc tests accuracy:**

| **Accuracy** | **Bonferroni adjustment: p<0.005** | | |
| --- | --- | --- | --- |
| **Comparisons (test)** | **t** | **df** | **Sig.** |
| *trial 0-20 vs 20-40* | *5.12* | *47* | *<0.001* |
| trial 0-20 vs 40-60 | 0.98 | 47 | 0.33 |
| *trial 0-20 vs 60-80* | *4.76* | *47* | *<0.001* |
| trial 0-20 vs 80-100 | 1.45 | 47 | 0.16 |
| *trial 20-40 vs 40-60* | *-4.22* | *47* | *<0.001* |
| trial 20-40 vs 60-80 | 0.44 | 47 | 0.66 |
| trial 20-40 vs 80-100 | -2.58 | 47 | 0.01 |
| *trial 40-60 vs 60-80* | *3.67* | *47* | *0.001* |
| trial 40-60 vs 80-100 | 0.68 | 47 | 0.50 |
| *trial 60-80 vs 80-100* | *-3.03* | *47* | *0.004* |

| **Accuracy** |  |  |  |  |  |  |  |  |  |
| --- | --- | --- | --- | --- | --- | --- | --- | --- | --- |
|  |  | **Male** | |  |  | **Female** | |  |  |
| **Source** |  | **df** | **df** | **F** | **Sig.** | **df** | **df** | **F** | **Sig.** |
| Test |  | *1* | *23* | *10.29* | *<0.001* | 1 | 21 | 1.80 | 0.14 |
| Treatment | | 1 | 23 | 0.00 | 0.98 | 1 | 21 | 0.14 | 0.71 |
| Test * Treatment | | 1 | 23 | 0.86 | 0.49 | 1 | 21 | 0.99 | 0.42 |

| **Accuracy: male, test**  **Bonferroni adjustment: p<0.005** | | |  |
| --- | --- | --- | --- |
| **Comparisons (test)** | **t** | **df** | **Sig.** |
| *trial 0-20 vs 20-40* | *4.47* | *24* | *<0.001* |
| trial 0-20 vs 40-60 | 1.06 | 24 | 0.30 |
| *trial 0-20 vs 60-80* | *4.14* | *24* | *<0.001* |
| trial 0-20 vs 80-100 | -0.07 | 24 | 0.94 |
| *trial 20-40 vs 40-60* | *-3.65* | *24* | *0.001* |
| trial 20-40 vs 60-80 | 0.06 | 24 | 0.96 |
| *trial 20-40 vs 80-100* | *-4.83* | *24* | *<0.001* |
| trial 40-60 vs 60-80 | 2.75 | 24 | 0.01 |
| trial 40-60 vs 80-100 | -1.36 | 24 | 0.19 |
| *trial 60-80 vs 80-100* | *-4.05* | *24* | *<0.001* |

| **Parameters** | |  |  |  |  |  |  | **Statistics** |  |  |  |  |
| --- | --- | --- | --- | --- | --- | --- | --- | --- | --- | --- | --- | --- |
| **Premature** |  |  |  |  |  |  |  |  |  |  |  |  |
| **Stim. dur.** | **Trial** | **Sex** | **Treatment** | **Mean** | **STDV** | **N** |  | **Factors** | **df** | **df** | **F** | **Sig.** |
| **1.2 sec** | 0-20 | Male | Control | 2.00 | 2.27 | 13 |  | Test | 1 | 44 | 0.79 | 0.53 |
|  |  |  | WMI | 0.92 | 1.38 | 12 |  | Sex | 1 | 44 | 0.90 | 0.35 |
|  |  | Female | Control | 1.80 | 2.21 | 15 |  | *Treatment* | *1* | *44* | *3.99* | *0.05* |
|  |  |  | WMI | 0.25 | 0.46 | 8 |  | Sex * Treatment | 1 | 44 | 1.74 | 0.19 |
| **0.5 sec** | 20-40 | Male | Control | 2.85 | 3.26 | 13 |  | Test * Sex | 1 | 44 | 0.69 | 0.60 |
|  |  |  | WMI | 1.00 | 1.35 | 12 |  | Test * Treatment | 1 | 44 | 2.00 | 0.10 |
|  |  | Female | Control | 1.47 | 1.92 | 15 |  | Test * Sex * Treatment | 1 | 44 | 1.25 | 0.29 |
|  |  |  | WMI | 1.00 | 0.76 | 8 |  |  |  |  |  |  |
| **1.2 sec** | 40-60 | Male | Control | 1.77 | 1.74 | 13 |  |  |  |  |  |  |
|  |  |  | WMI | 0.92 | 1.00 | 12 |  |  |  |  |  |  |
|  |  | Female | Control | 0.93 | 1.33 | 15 |  |  |  |  |  |  |
|  |  |  | WMI | 2.13 | 3.00 | 8 |  |  |  |  |  |  |
| **0.5 sec** | 60-80 | Male | Control | 2.08 | 2.53 | 13 |  |  |  |  |  |  |
|  |  |  | WMI | 1.25 | 0.97 | 12 |  |  |  |  |  |  |
|  |  | Female | Control | 1.53 | 1.25 | 15 |  |  |  |  |  |  |
|  |  |  | WMI | 1.38 | 0.92 | 8 |  |  |  |  |  |  |
| **1.2 sec** | 80-100 | Male | Control | 2.15 | 1.72 | 13 |  |  |  |  |  |  |
|  |  |  | WMI | 0.75 | 1.06 | 12 |  |  |  |  |  |  |
|  |  | Female | Control | 1.00 | 1.20 | 15 |  |  |  |  |  |  |
|  |  |  | WMI | 0.75 | 0.89 | 8 |  |  |  |  |  |  |

**Post hoc tests: Premature responses**

| **Premature** | |  |  |  |  |  |  |  |  |
| --- | --- | --- | --- | --- | --- | --- | --- | --- | --- |
|  |  | **Control** | |  |  | **WMI** | |  |  |
| **Source** |  | **df** |  | **F** | **Sig.** | **df** |  | **F** | **Sig.** |
| Test |  | 1 | 26 | 1.14 | 0.34 | 1 | 18 | 1.90 | 0.12 |
| Sex |  | 1 | 26 | 2.16 | 0.15 | 1 | 18 | 0.17 | 0.69 |
| Test * Sex | | 1 | 26 | 0.66 | 0.62 | 1 | 18 | 1.45 | 0.23 |

| **Parameters** | |  |  |  |  |  |  | **Statistics** |  |  |  |  |
| --- | --- | --- | --- | --- | --- | --- | --- | --- | --- | --- | --- | --- |
| **Omissions** |  |  |  |  |  |  |  |  |  |  |  |  |
| **Stim. dur.** | **Trial** | **Sex** | **Treatment** | **Mean** | **STDV** | **N** |  | **Factors** | **df** |  | **F** | **Sig.** |
| **1.2 sec** | 0-20 | Male | Control | 1.77 | 1.17 | 13 |  | *Test* | *1* | *44* | *17.32* | *<0.001* |
|  |  |  | WMI | 0.25 | 0.45 | 12 |  | Sex | 1 | 44 | 0.51 | 0.48 |
|  |  | Female | Control | 0.67 | 0.82 | 15 |  | *Treatment* | *1* | *44* | *8.09* | *0.01* |
|  |  |  | WMI | 0.88 | 0.64 | 8 |  | *Sex * Treatment* | *1* | *44* | *4.92* | *0.03* |
| **0.5 sec** | 20-40 | Male | Control | 3.54 | 2.47 | 13 |  | Test * Sex | 1 | 44 | 1.96 | 0.10 |
|  |  |  | WMI | 1.33 | 1.61 | 12 |  | Test * Treatment | 1 | 44 | 0.98 | 0.42 |
|  |  | Female | Control | 1.93 | 1.53 | 15 |  | Test * Sex * Treatment | 1 | 44 | 0.87 | 0.49 |
|  |  |  | WMI | 1.50 | 1.20 | 8 |  |  |  |  |  |  |
| **1.2 sec** | 40-60 | Male | Control | 1.08 | 1.38 | 13 |  |  |  |  |  |  |
|  |  |  | WMI | 0.42 | 0.51 | 12 |  |  |  |  |  |  |
|  |  | Female | Control | 0.33 | 0.72 | 15 |  |  |  |  |  |  |
|  |  |  | WMI | 0.13 | 0.35 | 8 |  |  |  |  |  |  |
| **0.5 sec** | 60-80 | Male | Control | 2.31 | 1.75 | 13 |  |  |  |  |  |  |
|  |  |  | WMI | 1.25 | 1.22 | 12 |  |  |  |  |  |  |
|  |  | Female | Control | 2.33 | 1.84 | 15 |  |  |  |  |  |  |
|  |  |  | WMI | 2.38 | 1.51 | 8 |  |  |  |  |  |  |
| **1.2 sec** | 80-100 | Male | Control | 1.15 | 1.46 | 13 |  |  |  |  |  |  |
|  |  |  | WMI | 0.42 | 0.79 | 12 |  |  |  |  |  |  |
|  |  | Female | Control | 1.00 | 1.85 | 15 |  |  |  |  |  |  |
|  |  |  | WMI | 0.63 | 0.74 | 8 |  |  |  |  |  |  |

**Post hoc tests: Omissions**

| **Omissions** | **Bonferroni adjustment p<0.005** | | |
| --- | --- | --- | --- |
| **Comparisons (test)** | **t** | **df** | **Sig.** |
| *trial 0-20 vs 20-40* | *-4.30* | *47* | *<0.001* |
| trial 0-20 vs 40-60 | 2.05 | 47 | 0.05 |
| *trial 0-20 vs 60-80* | *-4.64* | *47* | *<0.001* |
| trial 0-20 vs 80-100 | 0.27 | 47 | 0.79 |
| *trial 20-40 vs 40-60* | *6.84* | *47* | *<0.001* |
| trial 20-40 vs 60-80 | 0.28 | 47 | 0.78 |
| *trial 20-40 vs 80-100* | *4.43* | *47* | *<0.001* |
| *trial 40-60 vs 60-80* | *-6.38* | *47* | *<0.001* |
| trial 40-60 vs 80-100 | -1.53 | 47 | 0.13 |
| *trial 60-80 vs 80-100* | *4.82* | *47* | *<0.001* |

| **Omissions** | |  |  |  |  |  |  |  |  |
| --- | --- | --- | --- | --- | --- | --- | --- | --- | --- |
|  |  | **Male** | |  |  | **Female** | |  |  |
| **Source** |  | **df** | **df** | **F** | **Sig.** | **df** | **df** | **F** | **Sig.** |
| *Test* |  | *1* | *23* | *7.88* | *<0.001* | *1* | *21* | *12.13* | *<0.001* |
| Treatment | | *1* | *23* | *14.72* | *0.001* | 1 | 21 | 0.17 | 0.68 |
| Test * Treatment | | 1 | 23 | 1.49 | 0.21 | 1 | 21 | 0.32 | 0.87 |

| **Omissions** | **Bonferroni correction p<0.005** | | | | | |
| --- | --- | --- | --- | --- | --- | --- |
|  | **Males** | |  | **Females** | |  |
| **Comparisons (test)** | **t** | **df** | **Sig.** | **t** | **df** | **Sig.** |
| *trial 0-20 vs 20-40* | *-2.96* | *24* | *0.007* | *-3.43* | *22* | *0.002* |
| trial 0-20 vs 40-60 | 0.98 | 24 | 0.34 | 2.12 | 22 | 0.05 |
| *trial 0-20 vs 60-80* | *-2.32* | *24* | *0.029* | *-4.34* | *22* | *<0.001* |
| trial 0-20 vs 80-100 | 0.86 | 24 | 0.40 | -0.35 | 22 | 0.73 |
| *trial 20-40 vs 40-60* | *4.32* | *24* | *<0.001* | *6.08* | *22* | *<0.001* |
| trial 20-40 vs 60-80 | 1.48 | 24 | 0.15 | -1.77 | 22 | 0.09 |
| *trial 20-40 vs 80-100* | *3.56* | *24* | *0.002* | 2.70 | 22 | 0.01 |
| *trial 40-60 vs 60-80* | *-3.26* | *24* | *0.003* | *-6.17* | *22* | *<0.001* |
| trial 40-60 vs 80-100 | -0.14 | 24 | 0.89 | -2.18 | 22 | 0.04 |
| *trial 60-80 vs 80-100* | *2.97* | *24* | *0.007* | *3.82* | *22* | *0.001* |

| **Parameters** | |  |  |  |  |  |  | **Statistics** |  |  |  |  |
| --- | --- | --- | --- | --- | --- | --- | --- | --- | --- | --- | --- | --- |
| **Perservative responses** | | | | | | | | | | | | |
| **Stim. dur.** | **Trial** | **Sex** | **Treatment** | **Mean** | **STDV** | **N** |  | **Factors** | **df** | **df** | **F** | **Sig.** |
| **1.2 sec** | 0-20 | Male | Control | 1.69 | 1.93 | 13 |  | Test | 1 | 44 | 0.95 | 0.44 |
|  |  |  | WMI | 0.67 | 0.98 | 12 |  | Sex | 1 | 44 | 3.21 | 0.08 |
|  |  | Female | Control | 2.20 | 2.27 | 15 |  | Treatment | 1 | 44 | 0.95 | 0.34 |
|  |  |  | WMI | 2.00 | 2.51 | 8 |  | Sex * Treatment | 1 | 44 | 0.51 | 0.48 |
| **0.5 sec** | 20-40 | Male | Control | 1.08 | 1.26 | 13 |  | Test * Sex | 1 | 44 | 0.46 | 0.77 |
|  |  |  | WMI | 0.67 | 0.78 | 12 |  | Test * Treatment | 1 | 44 | 1.32 | 0.27 |
|  |  | Female | Control | 1.47 | 1.68 | 15 |  | Test * Sex * Treatment | 1 | 44 | 0.38 | 0.83 |
|  |  |  | WMI | 2.13 | 1.36 | 8 |  |  |  |  |  |  |
| **1.2 sec** | 40-60 | Male | Control | 1.62 | 1.89 | 13 |  |  |  |  |  |  |
|  |  |  | WMI | 0.75 | 0.87 | 12 |  |  |  |  |  |  |
|  |  | Female | Control | 2.27 | 2.55 | 15 |  |  |  |  |  |  |
|  |  |  | WMI | 1.38 | 1.77 | 8 |  |  |  |  |  |  |
| **0.5 sec** | 60-80 | Male | Control | 1.15 | 1.72 | 13 |  |  |  |  |  |  |
|  |  |  | WMI | 0.83 | 0.94 | 12 |  |  |  |  |  |  |
|  |  | Female | Control | 1.07 | 1.62 | 15 |  |  |  |  |  |  |
|  |  |  | WMI | 1.50 | 2.73 | 8 |  |  |  |  |  |  |
| **1.2 sec** | 80-100 | Male | Control | 1.38 | 1.04 | 13 |  |  |  |  |  |  |
|  |  |  | WMI | 0.75 | 0.75 | 12 |  |  |  |  |  |  |
|  |  | Female | Control | 2.00 | 1.89 | 15 |  |  |  |  |  |  |
|  |  |  | WMI | 1.50 | 2.73 | 8 |  |  |  |  |  |  |

**Table 10: Distractor 1: fixed block**

| **Parameters** | |  |  |  |  |  |  | **Statistics** |  |  |  |  |
| --- | --- | --- | --- | --- | --- | --- | --- | --- | --- | --- | --- | --- |
| **Accurary** | |  |  |  |  |  |  |  |  |  |  |  |
|  |  |  | **Baseline** | | **Test** |  |  | **Factors** | **df** | **df** | **F** | **Sig.** |
| **N** | **Sex** | **Treatment** | **Mean** | **STDV** | **Mean** | **STDV** |  | *Test* | *1* | *44* | *10.69* | *0.002* |
| 13 | Male | Control | 94.10 | 3.63 | 92.50 | 4.59 |  | Sex | 1 | 44 | 0.01 | 0.94 |
| 12 |  | WMI | 96.82 | 3.00 | 95.53 | 4.05 |  | *Treatment* | *1* | *44* | *5.25* | *0.03* |
| 15 | Female | Control | 95.76 | 2.43 | 92.69 | 3.80 |  | Sex * Treatment | 1 | 44 | 0.94 | 0.34 |
| 8 |  | WMI | 96.70 | 2.87 | 94.09 | 4.98 |  | Test * Sex | 1 | 44 | 1.14 | 0.29 |
|  |  |  |  |  |  |  |  | Test * Treatment | 1 | 44 | 0.09 | 0.77 |
|  |  |  |  |  |  |  |  | Test * Sex * Treatment | 1 | 44 | 0.00 | 0.96 |
| **Premature** | |  |  |  |  |  |  |  |  |  |  |  |
|  |  |  | **Baseline** | |  |  |  | **Factors** | **df** | **df** | **F** | **Sig.** |
| **N** | **Sex** | **Treatment** | **Mean** | **STDV** | **Mean** | **STDV** |  | Test | 1 | 44 | 0.45 | 0.51 |
| 13 | Male | Control | 3.69 | 2.63 | 4.46 | 4.27 |  | Sex | 1 | 44 | 0.79 | 0.38 |
| 12 |  | WMI | 1.67 | 1.61 | 1.83 | 2.17 |  | Treatment | 1 | 44 | 2.24 | 0.14 |
| 15 | Female | Control | 1.93 | 1.98 | 2.40 | 1.35 |  | Sex * Treatment | 1 | 44 | 3.57 | 0.07 |
| 8 |  | WMI | 2.63 | 3.38 | 2.25 | 3.33 |  | Test * Sex | 1 | 44 | 0.30 | 0.59 |
|  |  |  |  |  |  |  |  | Test * Treatment | 1 | 44 | 0.88 | 0.35 |
|  |  |  |  |  |  |  |  | Test * Sex * Treatment | 1 | 44 | 0.02 | 0.88 |
| **Omissions** | |  |  |  |  |  |  |  |  |  |  |  |
|  |  |  | **Baseline** | | **Test** |  |  | **Factors** | **df** | **df** | **F** | **Sig.** |
| **N** | **Sex** | **Treatment** | **Mean** | **STDV** | **Mean** | **STDV** |  | *Test* | *1* | *44* | *27.44* | *<0.001* |
| 13 | Male | Control | 7.00 | 5.02 | 21.69 | 15.93 |  | Sex | 1 | 44 | 0.60 | 0.44 |
| 12 |  | WMI | 4.33 | 3.63 | 11.25 | 8.90 |  | *Treatment* | *1* | *44* | *4.08* | *0.05* |
| 15 | Female | Control | 6.73 | 3.99 | 13.33 | 6.86 |  | Sex * Treatment | 1 | 44 | 2.62 | 0.11 |
| 8 |  | WMI | 6.38 | 3.07 | 12.25 | 9.11 |  | Test * Sex | 1 | 44 | 1.97 | 0.17 |
|  |  |  |  |  |  |  |  | Test * Treatment | 1 | 44 | 1.71 | 0.20 |
|  |  |  |  |  |  |  |  | Test * Sex * Treatment | 1 | 44 | 1.17 | 0.28 |
| **Preserverative responses** | | | |  |  |  |  |  |  |  |  |  |
|  |  |  | **Baseline** | | **Test** |  |  | **Factors** | **df** | **df** | **F** | **Sig.** |
| **N** | **Sex** | **Treatment** | **Mean** | **STDV** | **Mean** | **STDV** |  | Test | 1 | 44 | 0.68 | 0.42 |
| 13 | Male | Control | 4.00 | 3.16 | 4.15 | 3.95 |  | *Sex* | *1* | *44* | *9.83* | *0.003* |
| 12 |  | WMI | 4.25 | 2.73 | 4.17 | 3.41 |  | Treatment | 1 | 44 | 0.22 | 0.64 |
| 15 | Female | Control | 10.47 | 10.39 | 11.80 | 10.24 |  | Sex * Treatment | 1 | 44 | 0.29 | 0.59 |
| 8 |  | WMI | 8.50 | 7.33 | 9.88 | 8.66 |  | Test * Sex | 1 | 44 | 0.61 | 0.44 |
|  |  |  |  |  |  |  |  | Test * Treatment | 1 | 44 | 0.00 | 0.95 |
|  |  |  |  |  |  |  |  | Test * Sex * Treatment | 1 | 44 | 0.01 | 0.94 |

| **Parameters** | |  |  |  |  |  |  | **Statistics** |  |  |  |  |
| --- | --- | --- | --- | --- | --- | --- | --- | --- | --- | --- | --- | --- |
| **Latency to correct** | | |  |  |  |  |  |  |  |  |  |  |
|  |  |  | **Baseline** | | **Test** |  |  | **Factors** | **df** | **df** | **F** | **Sig.** |
| **N** | **Sex** | **Treatment** | **Mean** | **STDV** | **Mean** | **STDV** |  | *Test* | *1* | *44* | *20.37* | *<0.001* |
| 13 | Male | Control | 0.61 | 0.08 | 0.70 | 0.11 |  | Sex | 1 | 44 | 0.05 | 0.83 |
| 12 |  | WMI | 0.63 | 0.11 | 0.70 | 0.16 |  | Treatment | 1 | 44 | 0.42 | 0.52 |
| 15 | Female | Control | 0.65 | 0.08 | 0.70 | 0.12 |  | Sex * Treatment | 1 | 44 | 0.76 | 0.39 |
| 8 |  | WMI | 0.62 | 0.11 | 0.63 | 0.12 |  | *Test * Sex* | *1* | *44* | *4.42* | *0.04* |
|  |  |  |  |  |  |  |  | Test * Treatment | 1 | 44 | 1.44 | 0.24 |
|  |  |  |  |  |  |  |  | Test * Sex * Treatment | 1 | 44 | 0.22 | 0.64 |
| **Latency to reward** | | |  |  |  |  |  |  |  |  |  |  |
|  |  |  | **Baseline** | | **Test** |  |  | **Factors** | **df** | **df** | **F** | **Sig.** |
| **N** | **Sex** | **Treatment** | **Mean** | **STDV** | **Mean** | **STDV** |  | *Test* | *1* | *44* | *16.21* | *<0.001* |
| 13 | Male | Control | 1.78 | 0.57 | 2.74 | 1.05 |  | Sex | 1 | 44 | 0.05 | 0.83 |
| 12 |  | WMI | 1.44 | 0.31 | 2.16 | 0.50 |  | Treatment | 1 | 44 | 2.61 | 0.11 |
| 15 | Female | Control | 2.14 | 1.29 | 2.34 | 1.12 |  | Sex * Treatment | 1 | 44 | 0.09 | 0.77 |
| 8 |  | WMI | 1.76 | 0.66 | 2.09 | 1.27 |  | *Test * Sex* | *1* | *44* | *4.40* | *0.04* |
|  |  |  |  |  |  |  |  | Test * Treatment | 1 | 44 | 0.04 | 0.85 |
|  |  |  |  |  |  |  |  | Test * Sex * Treatment | 1 | 44 | 0.44 | 0.51 |

**Post hoc tests:**

| **Omissions** | |  |  |  |  |  |  |  |  |
| --- | --- | --- | --- | --- | --- | --- | --- | --- | --- |
|  |  | **Ctrl** |  |  |  | **WMI** |  |  |  |
| **Factors** |  | **df** | **df** | **F** | **Sig.** | **df** | **df** | **F** | **Sig.** |
| *Test* |  | *1* | *26* | *19.93* | *<0.001* | *1* | *18* | *12.02* | *0.003* |
| Sex |  | 1 | 26 | 3.10 | 0.09 | 1 | 18 | 0.37 | 0.55 |
| Test * Sex | | 1 | 26 | 2.88 | 0.10 | 1 | 18 | 0.08 | 0.78 |

| **Preserverative responses** | | | |  |  |  |  |  |  |
| --- | --- | --- | --- | --- | --- | --- | --- | --- | --- |
|  |  | **Male** | |  |  | **Female** | |  |  |
| **Factors** |  | **df** | **df** | **F** | **Sig.** | **df** | **df** | **F** | **Sig.** |
| Test |  | 1 | 23 | 0.00 | 0.96 | 1 | 21 | 0.69 | 0.42 |
| Treatment | | 1 | 23 | 0.01 | 0.91 | 1 | 21 | 0.25 | 0.62 |
| Test * Treatment | | 1 | 23 | 0.03 | 0.86 | 1 | 21 | 0.00 | 0.99 |

| **Latency to correct** | |  |  |  | |  | |  | |  | |  | |  | |
| --- | --- | --- | --- | --- | --- | --- | --- | --- | --- | --- | --- | --- | --- | --- | --- |
|  |  | **Male** | |  | |  | | **Female** | | | |  | |  | |
| **Factors** |  | **df** | **df** | **F** | | **Sig.** | | **df** | | **df** | | **F** | | **Sig.** | |
| Test |  | *1* | *23* | *21.22* | | *<0.001* | | 1 | | 21 | | 3.12 | | 0.09 | |
| Treatment | | 1 | 23 | 0.03 | | 0.87 | | 1 | | 21 | | 1.18 | | 0.29 | |
| Test * Treatment | | 1 | 23 | 0.26 | | 0.61 | | 1 | | 21 | | 1.49 | | 0.24 | |
|  |  |  |  |  | |  | |  | |  | |  | |  | |
| **Latency to reward** | |  |  |  |  | |  | |  | | | | | |  |
|  |  | **Male** | |  |  | | **Female** | | | |  | |  | |  |
| **Factors** |  | **df** | **df** | **F** | **Sig.** | | **df** | | **df** | | **F** | | **Sig.** | |  |
| Test |  | *1* | *23* | *67.26* | *<0.001* | | 1 | | 21 | | 0.97 | | 0.34 | |  |
| Treatment | | 1 | 23 | 3.32 | 0.08 | | 1 | | 21 | | 0.56 | | 0.46 | |  |
| Test * Treatment | | 1 | 23 | 1.33 | 0.26 | | 1 | | 21 | | 0.06 | | 0.81 | |  |

**Table 11: distractor 2: loose block**

| **Parameters** | |  |  |  |  |  |  | **Statistics** |  |  |  |  |
| --- | --- | --- | --- | --- | --- | --- | --- | --- | --- | --- | --- | --- |
| **Accurary** | |  |  |  |  |  |  |  |  |  |  |  |
|  |  |  | **Baseline** | | **Test** |  |  | **Factors** | **df** | **df** | **F** | **Sig.** |
| **N** | **Sex** | **Treatment** | **Mean** | **STDV** | **Mean** | **STDV** |  | *Test* | *1* | *44* | *12.46* | *0.001* |
| 13 | Male | Control | 94.31 | 4.25 | 90.43 | 5.75 |  | Sex | 1 | 44 | 0.51 | 0.48 |
| 12 |  | WMI | 94.78 | 3.06 | 88.88 | 13.54 |  | Treatment | 1 | 44 | 0.02 | 0.88 |
| 15 | Female | Control | 94.79 | 3.23 | 91.61 | 5.10 |  | Sex * Treatment | 1 | 44 | 0.04 | 0.85 |
| 8 |  | WMI | 96.73 | 1.75 | 89.79 | 13.13 |  | Test * Sex | 1 | 44 | 0.00 | 0.95 |
|  |  |  |  |  |  |  |  | Test * Treatment | 1 | 44 | 1.05 | 0.31 |
|  |  |  |  |  |  |  |  | Test * Sex * Treatment | 1 | 44 | 0.10 | 0.76 |
| **Premature** | |  |  |  |  |  |  |  |  |  |  |  |
|  |  |  | **Baseline** | | **Test** |  |  | **Factors** | **df** | **df** | **F** | **Sig.** |
| **N** | **Sex** | **Treatment** | **Mean** | **STDV** | **Mean** | **STDV** |  | *Test* | *1* | *44* | *6.43* | *0.02* |
| 13 | Male | Control | 4.00 | 3.00 | 6.08 | 7.27 |  | Sex | 1 | 44 | 0.60 | 0.44 |
| 12 |  | WMI | 2.42 | 2.02 | 6.08 | 11.52 |  | Treatment | 1 | 44 | 0.32 | 0.58 |
| 15 | Female | Control | 2.80 | 4.55 | 5.13 | 3.89 |  | Sex * Treatment | 1 | 44 | 0.00 | 0.98 |
| 8 |  | WMI | 2.13 | 2.17 | 4.38 | 2.72 |  | Test * Sex | 1 | 44 | 0.08 | 0.78 |
|  |  |  |  |  |  |  |  | Test * Treatment | 1 | 44 | 0.14 | 0.71 |
|  |  |  |  |  |  |  |  | Test * Sex * Treatment | 1 | 44 | 0.17 | 0.68 |
| **Omissions** | |  |  |  |  |  |  |  |  |  |  |  |
|  |  |  | **Baseline** | | **Test** |  |  | **Factors** | **df** | **df** | **F** | **Sig.** |
| **N** | **Sex** | **Treatment** | **Mean** | **STDV** | **Mean** | **STDV** |  | *Test* | *1* | *44* | *15.90* | *<0.001* |
| 13 | Male | Control | 5.75 | 3.35 | 8.08 | 5.66 |  | Sex | 1 | 44 | 1.35 | 0.25 |
| 12 |  | WMI | 3.95 | 3.12 | 5.40 | 4.76 |  | Treatment | 1 | 44 | 1.41 | 0.24 |
| 15 | Female | Control | 5.06 | 3.25 | 9.53 | 7.38 |  | Sex * Treatment | 1 | 44 | 0.68 | 0.41 |
| 8 |  | WMI | 4.66 | 2.24 | 9.13 | 2.98 |  | Test * Sex | 1 | 44 | 2.61 | 0.11 |
|  |  |  |  |  |  |  |  | Test * Treatment | 1 | 44 | 0.08 | 0.78 |
|  |  |  |  |  |  |  |  | Test * Sex * Treatment | 1 | 44 | 0.07 | 0.79 |
| **Preserverative responses** | | | |  |  |  |  |  |  |  |  |  |
|  |  |  | **Baseline** | | **Test** |  |  | **Factors** | **df** | **df** | **F** | **Sig.** |
| **N** | **Sex** | **Treatment** | **Mean** | **STDV** | **Mean** | **STDV** |  | Test | 1 | 44 | 1.53 | 0.22 |
| 13 | Male | Control | 5.31 | 4.29 | 4.23 | 3.14 |  | *Sex* | *1* | *44* | *8.13* | *0.007* |
| 12 |  | WMI | 3.75 | 2.26 | 2.92 | 1.78 |  | Treatment | 1 | 44 | 2.44 | 0.13 |
| 15 | Female | Control | 8.87 | 9.60 | 11.80 | 8.65 |  | Sex * Treatment | 1 | 44 | 0.43 | 0.51 |
| 8 |  | WMI | 5.38 | 4.07 | 8.25 | 6.04 |  | *Test * Sex* | *1* | *44* | *6.00* | *0.02* |
|  |  |  |  |  |  |  |  | Test * Treatment | 1 | 44 | 0.00 | 0.95 |
|  |  |  |  |  |  |  |  | Test * Sex * Treatment | 1 | 44 | 0.01 | 0.92 |

| **Parameters** | |  |  |  |  |  |  | **Statistics** |  |  |  |  |
| --- | --- | --- | --- | --- | --- | --- | --- | --- | --- | --- | --- | --- |
| **Latency to correct** | | |  |  |  |  |  |  |  |  |  |  |
|  |  |  | **Baseline** | | **Test** |  |  | **Factors** | **df** | **df** | **F** | **Sig.** |
| **N** | **Sex** | **Treatment** | **Mean** | **STDV** | **Mean** | **STDV** |  | *Test* | *1* | *44* | *19.17* | *<0.001* |
| 13 | Male | Control | 0.62 | 0.06 | 0.65 | 0.06 |  | Sex | 1 | 44 | 0.69 | 0.41 |
| 12 |  | WMI | 0.60 | 0.10 | 0.67 | 0.13 |  | Treatment | 1 | 44 | 1.80 | 0.19 |
| 15 | Female | Control | 0.63 | 0.09 | 0.66 | 0.10 |  | Sex * Treatment | 1 | 44 | 2.00 | 0.17 |
| 8 |  | WMI | 0.58 | 0.08 | 0.58 | 0.07 |  | *Test * Sex* | *1* | *44* | *6.21* | *0.02* |
|  |  |  |  |  |  |  |  | Test * Treatment | 1 | 44 | 0.19 | 0.66 |
|  |  |  |  |  |  |  |  | *Test * Sex * Treatment* | *1* | *44* | *4.77* | *0.03* |
| **Latency to reward** | | |  |  |  |  |  |  |  |  |  |  |
|  |  |  | **Baseline** | | **Test** |  |  | **Factors** | **df** | **df** | **F** | **Sig.** |
| **N** | **Sex** | **Treatment** | **Mean** | **STDV** | **Mean** | **STDV** |  | *Test* | *1* | *44* | *21.82* | *<0.001* |
| 13 | Male | Control | 1.96 | 0.72 | 2.37 | 0.84 |  | Sex | 1 | 44 | 0.01 | 0.92 |
| 12 |  | WMI | 1.58 | 0.43 | 1.91 | 0.42 |  | *Treatment* | *1* | *44* | *4.70* | *0.04* |
| 15 | Female | Control | 2.02 | 1.44 | 2.84 | 2.28 |  | Sex * Treatment | 1 | 44 | 0.82 | 0.37 |
| 8 |  | WMI | 1.16 | 0.26 | 1.66 | 0.46 |  | Test * Sex | 1 | 44 | 1.75 | 0.19 |
|  |  |  |  |  |  |  |  | Test * Treatment | 1 | 44 | 0.84 | 0.37 |
|  |  |  |  |  |  |  |  | Test * Sex * Treatment | 1 | 44 | 0.27 | 0.61 |

**Post hoc tests:**

| **Preserverative responses** | | | | | | | | |  | |  | | |  | | |  | | |  | | |  | | |  |  |
| --- | --- | --- | --- | --- | --- | --- | --- | --- | --- | --- | --- | --- | --- | --- | --- | --- | --- | --- | --- | --- | --- | --- | --- | --- | --- | --- | --- |
|  |  | | **Male** | | | | | |  | |  | | | **Female** | | | | | |  | | |  | | |  |  |
| **Factors** |  | | **df** | | **df** | | | | **F** | | **Sig.** | | | **df** | | | **df** | | | **F** | | | **Sig.** | | |  |  |
| Test |  | | 1 | | 23 | | | | 2.14 | | 0.16 | | | 1 | | | 21 | | | 3.70 | | | 0.07 | | |  |  |
| Treatment | | | 1 | | 23 | | | | 1.92 | | 0.18 | | | 1 | | | 21 | | | 1.23 | | | 0.28 | | |  |  |
| Test * Treatment | | | 1 | | 23 | | | | 0.04 | | 0.85 | | | 1 | | | 21 | | | 0.00 | | | 0.99 | | |  |  |
| **Latency to correct** | | | |  | |  | |  | | | |  | | |  | | |  | | |  | | |  | | |  |
|  | |  | | **Male** | | | |  | | | |  | | | **Female** | | | | | |  | | |  | | |  |
| **Factors** | |  | | **df** | | **df** | | **F** | | | | **Sig.** | | | **df** | | | **df** | | | **F** | | | **Sig.** | | |  |
| Test | |  | | *1* | | *23* | | *22.53* | | | | *<0.001* | | | 1 | | | 21 | | | 1.96 | | | 0.18 | | |  |
| Treatment | | | | 1 | | 23 | | 0.00 | | | | 0.96 | | | 1 | | | 21 | | | 3.55 | | | 0.07 | | |  |
| Test * Treatment | | | | 1 | | 23 | | 3.28 | | | | 0.08 | | | 1 | | | 21 | | | 1.68 | | | 0.21 | | |  |
| **Latency to reward** | | | |  | | |  | | |  | | |  | | |  | | |  | | |  | | |  | | |
|  | |  | | **Ctrl** | | |  | | |  | | |  | | | **WMI** | | | | | |  | | |  | | |
| **Factors** | |  | | **df** | | | **df** | | | **F** | | | **Sig.** | | | **df** | | | **df** | | | **F** | | | **Sig.** | | |
| *Test* | |  | | *1* | | | *26* | | | *13.29* | | | *<0.001* | | | *1* | | | *18* | | | *16.24* | | | *0.001* | | |
| Sex | |  | | 1 | | | 26 | | | 0.24 | | | 0.63 | | | *1* | | | *18* | | | *4.70* | | | *0.04* | | |
| Test * Sex | | | | 1 | | | 26 | | | 1.45 | | | 0.24 | | | 1 | | | 18 | | | 0.74 | | | 0.40 | | |
