# Supplementary material for: Cognitive performance during adulthood in a rat model of neonatal diffuse white matter injury
Source: Psychopharmacology (Berl). 2022 Jan 22;239(3):745–64. doi: 10.1007/s00213-021-06053-w (PMC8891199; doi:10.1007/s00213-021-06053-w)
Supplement: Supplementary file 2 — Supplementary file2 (DOCX 85 KB) [file 213_2021_6053_MOESM2_ESM.docx]

| **Parameters** | | |  |  |  |  |  |  |  | **Statistics** |  |  |  |  |
| --- | --- | --- | --- | --- | --- | --- | --- | --- | --- | --- | --- | --- | --- | --- |
| **Nr. of trials pressing the high prob. lever** | | | | |  |  |  |  |  |  |  |  |  |  |
|  |  |  | **Males** |  |  | **Females** | |  |  | **Factors** | **df** | **df** | **F** | **Sig.** |
| **Day** | **Session** | **Treatment** | **Mean** | **STDV** | **N** | **Mean** | **STDV** | **N** |  | Sex | 1 | 44 | 1.72 | 0.20 |
| **1** | 0-40 | Control | 23.92 | 5.81 | 13 | 22.73 | 4.99 | 15 |  | Treatment | 1 | 44 | 2.58 | 0.12 |
|  |  | WMI | 24.50 | 6.23 | 12 | 22.75 | 6.41 | 8 |  | Sex * Treatment | 1 | 44 | 1.50 | 0.23 |
|  | 41-80 | Control | 17.15 | 6.26 | 13 | 18.27 | 3.39 | 15 |  | *Day* | *4* | *176* | *13.97* | *<0.001* |
|  |  | WMI | 17.67 | 4.98 | 12 | 15.75 | 6.36 | 8 |  | Day * Sex | 4 | 176 | 2.16 | 0.08 |
|  | 81-120 | Control | 24.38 | 7.62 | 13 | 20.87 | 6.69 | 15 |  | Day * Treatment | 4 | 176 | 0.62 | 0.65 |
|  |  | WMI | 24.50 | 7.50 | 12 | 24.88 | 7.61 | 8 |  | Day * Sex * Treatment | 4 | 176 | 1.00 | 0.41 |
| **2** | 0-40 | Control | 25.85 | 5.54 | 13 | 23.67 | 6.63 | 15 |  | *Session* | *2* | *88* | *48.00* | *<0.001* |
|  |  | WMI | 25.50 | 7.61 | 12 | 24.50 | 8.70 | 8 |  | Session * Sex | 2 | 88 | 0.48 | 0.62 |
|  | 41-80 | Control | 18.69 | 5.99 | 13 | 18.27 | 6.73 | 15 |  | Session * Treatment | 2 | 88 | 0.17 | 0.85 |
|  |  | WMI | 18.67 | 8.25 | 12 | 16.75 | 4.86 | 8 |  | Session * Sex * Treatment | 2 | 88 | 0.26 | 0.77 |
|  | 81-120 | Control | 24.00 | 6.75 | 13 | 21.47 | 7.38 | 15 |  | Day * Session | 8 | 352 | 0.21 | 0.99 |
|  |  | WMI | 25.75 | 6.63 | 12 | 23.25 | 8.35 | 8 |  | Day * Session * Sex | 8 | 352 | 0.07 | 1.00 |
| **3** | 0-40 | Control | 28.92 | 6.70 | 13 | 23.07 | 7.01 | 15 |  | Day * Session * Treatment | 8 | 352 | 0.26 | 0.98 |
|  |  | WMI | 24.00 | 7.53 | 12 | 27.13 | 7.14 | 8 |  | Day * Session * Sex * Treatment | 8 | 352 | 1.53 | 0.15 |
|  | 41-80 | Control | 18.85 | 7.56 | 13 | 20.07 | 8.18 | 15 |  |  |  |  |  |  |
|  |  | WMI | 22.83 | 7.87 | 12 | 19.50 | 7.89 | 8 |  |  |  |  |  |  |
|  | 81-120 | Control | 26.31 | 8.12 | 13 | 22.40 | 8.89 | 15 |  |  |  |  |  |  |
|  |  | WMI | 25.92 | 7.18 | 12 | 26.25 | 5.04 | 8 |  |  |  |  |  |  |
| **4** | 0-40 | Control | 25.92 | 7.50 | 13 | 27.40 | 5.93 | 15 |  |  |  |  |  |  |
|  |  | WMI | 27.83 | 6.38 | 12 | 26.13 | 8.27 | 8 |  |  |  |  |  |  |
|  | 41-80 | Control | 21.31 | 9.62 | 13 | 17.47 | 7.39 | 15 |  |  |  |  |  |  |
|  |  | WMI | 17.33 | 7.74 | 12 | 23.25 | 6.56 | 8 |  |  |  |  |  |  |
|  | 81-120 | Control | 25.92 | 8.12 | 13 | 27.33 | 6.47 | 15 |  |  |  |  |  |  |
|  |  | WMI | 28.83 | 7.33 | 12 | 23.25 | 4.06 | 8 |  |  |  |  |  |  |
| **5** | 0-40 | Control | 26.00 | 8.99 | 13 | 25.40 | 9.37 | 15 |  |  |  |  |  |  |
|  |  | WMI | 26.08 | 6.35 | 12 | 30.88 | 3.27 | 8 |  |  |  |  |  |  |
|  | 41-80 | Control | 21.23 | 8.25 | 13 | 21.53 | 8.34 | 15 |  |  |  |  |  |  |
|  |  | WMI | 21.92 | 8.90 | 12 | 24.13 | 5.79 | 8 |  |  |  |  |  |  |
|  | 81-120 | Control | 26.62 | 8.24 | 13 | 26.27 | 5.73 | 15 |  |  |  |  |  |  |
|  |  | WMI | 26.58 | 6.54 | 12 | 28.88 | 5.28 | 8 |  |  |  |  |  |  |

**Table 12:** **Number of trials pressing the high probability lever**

**Post hoc tests:**

| **Parameters** | |  |  | **Statistics** | |  |
| --- | --- | --- | --- | --- | --- | --- |
| **Day** |  |  |  |  |  |  |
| **Day** | **Mean** | **STDV** |  | **Day** | **vs. Day** | **Sig.** |
| 1 | 21.45 | 3.16 |  | 1 | 2 | 1.00 |
| 2 | 22.20 | 3.11 |  | *1* | *3* | *0.01* |
| 3 | 23.77 | 3.28 |  | *1* | *4* | *0.001* |
| 4 | 24.33 | 3.11 |  | *1* | *5* | *<0.001* |
| 5 | 25.46 | 3.27 |  | 2 | 3 | 0.07 |
|  |  |  |  | *2* | *4* | *0.01* |
|  |  |  |  | *2* | *5* | *<0.001* |
|  |  |  |  | 3 | 4 | 1.00 |
|  |  |  |  | 3 | 5 | 0.13 |
|  |  |  |  | 4 | 5 | 0.72 |
| **Session** |  |  |  |  |  |  |
| **Session** | **Mean** | **STDV** |  | **Session** | **vs. Session** | **Sig.** |
| 1 | 25.61 | 2.88 |  | *1* | *2* | *<0.001* |
| 2 | 19.53 | 3.25 |  | 1 | 3 | 0.79 |
| 3 | 25.18 | 3.62 |  | *2* | *3* | *<0.001* |

**Table 13: Reaction time**

| **Parameters** | |  |  |  |  |  | **Statistics** |  |  |  |  |
| --- | --- | --- | --- | --- | --- | --- | --- | --- | --- | --- | --- |
| **Reaction time** | |  |  |  |  |  |  |  |  |  |  |
| **Day** | **Sex** | **Treatment** | **Mean** | **STDV** | **N** |  | **Factors** | **df** | **df** | **F** | **Sig.** |
| 1 | Males | Control | 0.95 | 0.24 | 13 |  | *Sex* | *1* | *43* | *20.71* | *<0.001* |
|  |  | WMI | 1.02 | 0.32 | 12 |  | *Treatment* | *1* | *43* | *4.88* | *0.03* |
|  | Females | Control | 2.47 | 1.90 | 15 |  | *Sex * Treatment* | *1* | *43* | *5.82* | *0.02* |
|  |  | WMI | 1.33 | 0.29 | 7 |  | *Day* | *4* | *172* | *8.62* | *<0.001* |
| 2 | Males | Control | 0.89 | 0.24 | 13 |  | *Day * Sex* | *4* | *172* | *2.41* | *0.05* |
|  |  | WMI | 0.92 | 0.25 | 12 |  | Day * Treatment | 4 | 172 | 1.49 | 0.21 |
|  | Females | Control | 1.92 | 0.76 | 15 |  | Day * Sex * Treatment | 4 | 172 | 1.98 | 0.10 |
|  |  | WMI | 1.12 | 0.22 | 7 |  |  |  |  |  |  |
| 3 | Males | Control | 0.81 | 0.22 | 13 |  |  |  |  |  |  |
|  |  | WMI | 0.80 | 0.25 | 12 |  |  |  |  |  |  |
|  | Females | Control | 1.48 | 0.58 | 15 |  |  |  |  |  |  |
|  |  | WMI | 1.23 | 0.50 | 7 |  |  |  |  |  |  |
| 4 | Males | Control | 0.78 | 0.23 | 13 |  |  |  |  |  |  |
|  |  | WMI | 0.82 | 0.25 | 12 |  |  |  |  |  |  |
|  | Females | Control | 1.37 | 0.31 | 15 |  |  |  |  |  |  |
|  |  | WMI | 0.94 | 0.20 | 7 |  |  |  |  |  |  |
| 5 | Males | Control | 0.71 | 0.20 | 13 |  |  |  |  |  |  |
|  |  | WMI | 0.70 | 0.28 | 12 |  |  |  |  |  |  |
|  | Females | Control | 1.14 | 0.35 | 15 |  |  |  |  |  |  |
|  |  | WMI | 0.94 | 0.37 | 7 |  |  |  |  |  |  |

**Post hoc tests**

| **Reaction time** | |  |  |  |
| --- | --- | --- | --- | --- |
|  |  | **t** | **df** | **Sig.** |
| **Males: ctrl vs WMI** | | -0.30 | 23 | 0.77 |
| ***Females: ctrl vs WMI*** | | *2.96* | *20* | *0.01* |
| ***Control: M vs F*** | | *-5.05* | *26* | *<0.001* |
| ***WMI: M vs F*** | | *-2.18* | *17* | *0.04* |
| ***Day 1: M vs F*** | | *-3.35* | *45* | *0.002* |
| ***Day 2: M vs F*** | | *-4.86* | *45* | *<0.001* |
| ***Day 3: M vs F*** | | *-4.88* | *45* | *<0.001* |
| ***Day 4: M vs F*** | | *-5.06* | *45* | *<0.001* |
| ***Day 5: M vs F*** | | *-4.21* | *45* | *<0.001* |

**Table 14: Win-stay behaviour**

| **Parameters** | |  |  |  |  |  | **Statistics** |  |  |  |  |
| --- | --- | --- | --- | --- | --- | --- | --- | --- | --- | --- | --- |
| **Win-stay behaviour** | | |  |  |  |  |  |  |  |  |  |
| **Day** | **Sex** | **Treatment** | **Mean** | **STDV** | **N** |  | **Factors** | **df** | **df** | **F** | **Sig.** |
| 1 | Male | Control | 0.60 | 0.13 | 13 |  | Sex | 1 | 43 | 1.13 | 0.29 |
|  |  | WMI | 0.59 | 0.12 | 12 |  | Treatment | 1 | 43 | 0.06 | 0.81 |
|  | Female | Control | 0.52 | 0.10 | 15 |  | Sex * Treatment | 1 | 43 | 0.06 | 0.80 |
|  |  | WMI | 0.59 | 0.18 | 7 |  | *Day* | *4* | *172* | *27.31* | *<0.001* |
| 2 | Male | Control | 0.63 | 0.13 | 13 |  | Day * Sex | 4 | 172 | 0.21 | 0.93 |
|  |  | WMI | 0.63 | 0.15 | 12 |  | Day * Treatment | 4 | 172 | 0.61 | 0.66 |
|  | Female | Control | 0.61 | 0.10 | 15 |  | Day * Sex * Treatment | 4 | 172 | 0.40 | 0.81 |
|  |  | WMI | 0.60 | 0.17 | 7 |  |  |  |  |  |  |
| 3 | Male | Control | 0.69 | 0.12 | 13 |  |  |  |  |  |  |
|  |  | WMI | 0.65 | 0.13 | 12 |  |  |  |  |  |  |
|  | Female | Control | 0.65 | 0.12 | 15 |  |  |  |  |  |  |
|  |  | WMI | 0.63 | 0.11 | 7 |  |  |  |  |  |  |
| 4 | Male | Control | 0.74 | 0.12 | 13 |  |  |  |  |  |  |
|  |  | WMI | 0.75 | 0.15 | 12 |  |  |  |  |  |  |
|  | Female | Control | 0.72 | 0.11 | 15 |  |  |  |  |  |  |
|  |  | WMI | 0.71 | 0.12 | 7 |  |  |  |  |  |  |
| 5 | Male | Control | 0.77 | 0.10 | 13 |  |  |  |  |  |  |
|  |  | WMI | 0.80 | 0.12 | 12 |  |  |  |  |  |  |
|  | Female | Control | 0.77 | 0.12 | 15 |  |  |  |  |  |  |
|  |  | WMI | 0.79 | 0.12 | 7 |  |  |  |  |  |  |

**Table 15: Pellets won (fraction)**

| **Parameters** | |  |  |  |  |  | **Statistics** |  |  |  |  |
| --- | --- | --- | --- | --- | --- | --- | --- | --- | --- | --- | --- |
| **Pellets won (fraction)** | | |  |  |  |  |  |  |  |  |  |
| **Day** | **Sex** | **Treatment** | **Mean** | **STDV** | **N** |  | **Factors** | **df** | **df** | **F** | **Sig.** |
| 1 | Males | Control | 0.54 | 0.06 | 13 |  | Sex | 1 | 43 | 1.36 | 0.25 |
|  |  | WMI | 0.52 | 0.06 | 12 |  | Treatment | 1 | 43 | 0.28 | 0.60 |
|  | Females | Control | 0.50 | 0.05 | 15 |  | Sex * Treatment | 1 | 43 | 1.90 | 0.18 |
|  |  | WMI | 0.53 | 0.05 | 7 |  | *Day* | *4* | *172* | *5.55* | *<0.001* |
| 2 | Males | Control | 0.54 | 0.05 | 13 |  | Day * Sex | 4 | 172 | 0.67 | 0.61 |
|  |  | WMI | 0.54 | 0.05 | 12 |  | Day * Treatment | 4 | 172 | 0.71 | 0.59 |
|  | Females | Control | 0.52 | 0.06 | 15 |  | Day * Sex * Treatment | 4 | 172 | 0.39 | 0.81 |
|  |  | WMI | 0.54 | 0.07 | 7 |  |  |  |  |  |  |
| 3 | Males | Control | 0.56 | 0.06 | 13 |  |  |  |  |  |  |
|  |  | WMI | 0.56 | 0.05 | 12 |  |  |  |  |  |  |
|  | Females | Control | 0.51 | 0.06 | 15 |  |  |  |  |  |  |
|  |  | WMI | 0.55 | 0.06 | 7 |  |  |  |  |  |  |
| 4 | Males | Control | 0.57 | 0.06 | 13 |  |  |  |  |  |  |
|  |  | WMI | 0.56 | 0.06 | 12 |  |  |  |  |  |  |
|  | Females | Control | 0.57 | 0.04 | 15 |  |  |  |  |  |  |
|  |  | WMI | 0.54 | 0.05 | 7 |  |  |  |  |  |  |
| 5 | Males | Control | 0.57 | 0.05 | 13 |  |  |  |  |  |  |
|  |  | WMI | 0.57 | 0.08 | 12 |  |  |  |  |  |  |
|  | Females | Control | 0.57 | 0.07 | 15 |  |  |  |  |  |  |
|  |  | WMI | 0.59 | 0.04 | 7 |  |  |  |  |  |  |

**Table 16: Lose-shift behaviour**

| **Parameters** | |  |  |  |  |  | **Statistics** |  |  |  |  |
| --- | --- | --- | --- | --- | --- | --- | --- | --- | --- | --- | --- |
| **Lose-shift behaviour** | | |  |  |  |  |  |  |  |  |  |
| **Day** | **Sex** | **Treatment** | **Mean** | **STDV** | **N** |  | **Source** | **df** | **df** | **F** | **Sig.** |
| 1 | Males | Control | 0.50 | 0.07 | 13 |  | Sex | 1 | 43 | 0.97 | 0.33 |
|  |  | WMI | 0.48 | 0.07 | 12 |  | Treatment | 1 | 43 | 0.16 | 0.69 |
|  | Females | Control | 0.50 | 0.06 | 15 |  | Sex * Treatment | 1 | 43 | 0.25 | 0.62 |
|  |  | WMI | 0.44 | 0.05 | 7 |  | Day | 4 | 172 | 0.70 | 0.59 |
| 2 | Males | Control | 0.54 | 0.07 | 13 |  | Day * Sex | 4 | 172 | 2.21 | 0.07 |
|  |  | WMI | 0.48 | 0.10 | 12 |  | *Day * Treatment* | *4* | *172* | *3.21* | *0.01* |
|  | Females | Control | 0.47 | 0.08 | 15 |  | Day * Sex * Treatment | 4 | 172 | 0.76 | 0.56 |
|  |  | WMI | 0.43 | 0.04 | 7 |  |  |  |  |  |  |
| 3 | Males | Control | 0.48 | 0.07 | 13 |  |  |  |  |  |  |
|  |  | WMI | 0.50 | 0.07 | 12 |  |  |  |  |  |  |
|  | Females | Control | 0.47 | 0.08 | 15 |  |  |  |  |  |  |
|  |  | WMI | 0.54 | 0.09 | 7 |  |  |  |  |  |  |
| 4 | Males | Control | 0.49 | 0.06 | 13 |  |  |  |  |  |  |
|  |  | WMI | 0.49 | 0.10 | 12 |  |  |  |  |  |  |
|  | Females | Control | 0.46 | 0.09 | 15 |  |  |  |  |  |  |
|  |  | WMI | 0.46 | 0.05 | 7 |  |  |  |  |  |  |
| 5 | Males | Control | 0.48 | 0.09 | 13 |  |  |  |  |  |  |
|  |  | WMI | 0.47 | 0.09 | 12 |  |  |  |  |  |  |
|  | Females | Control | 0.49 | 0.10 | 15 |  |  |  |  |  |  |
|  |  | WMI | 0.51 | 0.09 | 7 |  |  |  |  |  |  |

**Post hoc tests:**

| **Lose-shift behaviour** | |  |  |  |
| --- | --- | --- | --- | --- |
|  |  | **t** | **df** | **Sig.** |
| **Day 1: ctrl vs WMI** | | 1.79 | 45 | 0.08 |
| **Day 2: ctrl vs WMI** | | 1.50 | 45 | 0.14 |
| ***Day 3: ctrl vs WMI*** | | *-2.00* | *45* | *0.05* |
| **Day 4: ctrl vs WMI** | | -0.23 | 45 | 0.82 |
| **Day 5: ctrl vs WMI** | | -0.06 | 45 | 0.96 |

**Table S17: Reward learning (α+)**

| **Parameters** | |  |  |  |  |  | **Statistics** |  |  |  |  |  |
| --- | --- | --- | --- | --- | --- | --- | --- | --- | --- | --- | --- | --- |
| **Reward learning (α+)** | | |  |  |  |  |  |  |  |  |  |  |
| **Day** | **Sex** | **Treatment** | **Mean** | **STDV** | **N** |  | **Factors** |  | **df** | **df** | **F** | **Sig.** |
| 1 | Males | Control | 0,15 | 0,18 | 13 |  | Treatment | | 1 | 30 | 1,04 | 0,32 |
|  |  | WMI | 0,06 | 0,19 | 12 |  | Sex |  | 1 | 30 | 0,01 | 0,91 |
|  | Females | Control | 0,03 | 0,12 | 15 |  | Treatment * Sex | | 1 | 30 | 0,11 | 0,75 |
|  |  | WMI | 0,08 | 0,12 | 7 |  | *Day* |  | *4* | *120* | *4,00* | *0,004* |
| 2 | Males | Control | 0,13 | 0,42 | 13 |  | Day * Treatment | | 4 | 120 | 0,35 | 0,85 |
|  |  | WMI | 0,19 | 0,44 | 12 |  | Day *Sex |  | 4 | 120 | 0,19 | 0,94 |
|  | Females | Control | 0,10 | 0,28 | 15 |  | Day * Treatment * Sex | | 4 | 120 | 1,47 | 0,22 |
|  |  | WMI | 0,28 | 0,26 | 7 |  |  |  |  |  |  |  |
| 3 | Males | Control | 0,05 | 0,35 | 13 |  |  |  |  |  |  |  |
|  |  | WMI | 0,36 | 0,37 | 12 |  |  |  |  |  |  |  |
|  | Females | Control | 0,22 | 0,24 | 15 |  |  |  |  |  |  |  |
|  |  | WMI | 0,12 | 0,22 | 7 |  |  |  |  |  |  |  |
| 4 | Males | Control | 0,23 | 0,46 | 13 |  |  |  |  |  |  |  |
|  |  | WMI | 0,26 | 0,48 | 12 |  |  |  |  |  |  |  |
|  | Females | Control | 0,20 | 0,31 | 15 |  |  |  |  |  |  |  |
|  |  | WMI | 0,23 | 0,29 | 7 |  |  |  |  |  |  |  |
| 5 | Males | Control | 0,43 | 0,59 | 13 |  |  |  |  |  |  |  |
|  |  | WMI | 0,27 | 0,62 | 12 |  |  |  |  |  |  |  |
|  | Females | Control | 0,34 | 0,4 | 15 |  |  |  |  |  |  |  |
|  |  | WMI | 0,48 | 0,38 | 7 |  |  |  |  |  |  |  |

| **Post hoc tests** | |  |  |  |
| --- | --- | --- | --- | --- |
| **Reward learning (α+)** | |  |  |  |
|  |  | **t** | **df** | **Sig.** |
| **Day 1 vs 2** | | -1,8 | 45 | 0,99 |
| **Day 1 vs 3** | | -2,5 | 45 | 0,35 |
| **Day 1 vs 4** | | -2,6 | 45 | 0,32 |
| ***Day 1 vs 5*** | | *-4,2* | *45* | *0.01* |

**Table S18: Punishment learning (α-)**

| **Parameters** | |  |  |  |  |  | **Statistics** |  |  |  |  |  |
| --- | --- | --- | --- | --- | --- | --- | --- | --- | --- | --- | --- | --- |
| **Punishment learning (α-)** | | |  |  |  |  |  |  |  |  |  |  |
| **Day** | **Sex** | **Treatment** | **Mean** | **STDV** | **N** |  | **Factors** |  | **df** | **df** | **F** | **Sig.** |
| 1 | Males | Control | 0,18 | 0,54 | 13 |  | Treatment | | 1 | 30 | 1,77 | 0,19 |
|  |  | WMI | 0,39 | 0,33 | 12 |  | Sex |  | 1 | 30 | 0,13 | 0,73 |
|  | Females | Control | 0,28 | 0,64 | 15 |  | Treatment * Sex | | 1 | 30 | 0,24 | 0,63 |
|  |  | WMI | 0,27 | 0,34 | 7 |  | Day |  | 4 | 120 | 1,57 | 0,19 |
| 2 | Males | Control | 0,14 | 0,52 | 13 |  | Day * Treatment | | 4 | 120 | 0,31 | 0,87 |
|  |  | WMI | 0,12 | 0,55 | 12 |  | Day *Sex |  | 4 | 120 | 0,24 | 0,92 |
|  | Females | Control | 0,22 | 0,35 | 15 |  | Day * Treatment * Sex | | 4 | 120 | 0,16 | 0,96 |
|  |  | WMI | 0,18 | 0,33 | 7 |  |  |  |  |  |  |  |
| 3 | Males | Control | 0,16 | 0,50 | 13 |  |  |  |  |  |  |  |
|  |  | WMI | 0,39 | 0,52 | 12 |  |  |  |  |  |  |  |
|  | Females | Control | 0,20 | 0,34 | 15 |  |  |  |  |  |  |  |
|  |  | WMI | 0,32 | 0,31 | 7 |  |  |  |  |  |  |  |
| 4 | Males | Control | 0,13 | 0,51 | 13 |  |  |  |  |  |  |  |
|  |  | WMI | 0,23 | 0,54 | 12 |  |  |  |  |  |  |  |
|  | Females | Control | 0,23 | 0,35 | 15 |  |  |  |  |  |  |  |
|  |  | WMI | 0,36 | 0,33 | 7 |  |  |  |  |  |  |  |
| 5 | Males | Control | 0,41 | 0,63 | 13 |  |  |  |  |  |  |  |
|  |  | WMI | 0,44 | 0,66 | 12 |  |  |  |  |  |  |  |
|  | Females | Control | 0,35 | 0,43 | 15 |  |  |  |  |  |  |  |
|  |  | WMI | 0,40 | 0,40 | 7 |  |  |  |  |  |  |  |

**Table S19: Perservation (π)**

| **Parameters** | |  |  |  |  |  | **Statistics** |  |  |  |  |  |
| --- | --- | --- | --- | --- | --- | --- | --- | --- | --- | --- | --- | --- |
| **Perservation (π)** | |  |  |  |  |  |  |  |  |  |  |  |
| **Day** | **Sex** | **Treatment** | **Mean** | **STDV** | **N** |  | **Factors** |  | **df** | **df** | **F** | **Sig.** |
| 1 | Males | Control | -0,39 | 0,80 | 13 |  | Treatment | | 1 | 30 | 1,06 | 0,31 |
|  |  | WMI | 0,00 | 0,91 | 12 |  | Sex |  | 1 | 30 | 1,30 | 0,26 |
|  | Females | Control | -0,02 | 0,57 | 15 |  | Treatment * Sex | | 1 | 30 | 0,34 | 0,57 |
|  |  | WMI | 0,09 | 0,53 | 7 |  | Day |  | 4 | 120 | 1,59 | 0,18 |
| 2 | Males | Control | 0,02 | 0,18 | 13 |  | Day * Treatment | | 4 | 120 | 0,89 | 0,47 |
|  |  | WMI | 0,05 | 0,21 | 12 |  | Day *Sex |  | 4 | 120 | 0,50 | 0,74 |
|  | Females | Control | 0,04 | 0,13 | 15 |  | Day * Treatment * Sex | | 4 | 120 | 0,66 | 0,62 |
|  |  | WMI | 0,16 | 0,12 | 7 |  |  |  |  |  |  |  |
| 3 | Males | Control | 0,03 | 0,78 | 13 |  |  |  |  |  |  |  |
|  |  | WMI | 0,08 | 0,89 | 12 |  |  |  |  |  |  |  |
|  | Females | Control | 0,29 | 0,55 | 15 |  |  |  |  |  |  |  |
|  |  | WMI | -0,02 | 0,52 | 7 |  |  |  |  |  |  |  |
| 4 | Males | Control | 0,09 | 0,18 | 13 |  |  |  |  |  |  |  |
|  |  | WMI | 0,14 | 0,21 | 12 |  |  |  |  |  |  |  |
|  | Females | Control | 0,08 | 0,13 | 15 |  |  |  |  |  |  |  |
|  |  | WMI | 0,14 | 0,12 | 7 |  |  |  |  |  |  |  |
| 5 | Males | Control | 0,19 | 0,34 | 13 |  |  |  |  |  |  |  |
|  |  | WMI | 0,16 | 0,39 | 12 |  |  |  |  |  |  |  |
|  | Females | Control | 0,08 | 0,24 | 15 |  |  |  |  |  |  |  |
|  |  | WMI | 0,23 | 0,23 | 7 |  |  |  |  |  |  |  |

**Table S20: Explore/Exploit (β)**

| **Parameters** | |  |  |  |  |  | **Statistics** |  |  |  |  |  |
| --- | --- | --- | --- | --- | --- | --- | --- | --- | --- | --- | --- | --- |
| **Explore/exploit (β)** | |  |  |  |  |  |  |  |  |  |  |  |
| **Day** | **Sex** | **Treatment** | **Mean** | **STDV** | **N** |  | **Factors** |  | **df** | **df** | **F** | **Sig.** |
| 1 | Males | Control | 3,49 | 2,87 | 13 |  | Treatment | | 1 | 30 | 2,26 | 0,14 |
|  |  | WMI | 3,27 | 3,02 | 12 |  | Sex |  | 1 | 30 | 0,02 | 0,89 |
|  | Females | Control | 4,14 | 1,95 | 15 |  | Treatment * Sex | | 1 | 30 | 0,01 | 0,94 |
|  |  | WMI | 3,86 | 1,83 | 7 |  | Day |  | 4 | 120 | 1,32 | 0,27 |
| 2 | Males | Control | 3,97 | 2,22 | 13 |  | Day * Treatment | | 4 | 120 | 0,28 | 0,89 |
|  |  | WMI | 4,13 | 2,33 | 12 |  | Day *Sex |  | 4 | 120 | 0,79 | 0,53 |
|  | Females | Control | 4,54 | 1,51 | 15 |  | Day * Treatment * Sex | | 4 | 120 | 1,40 | 0,24 |
|  |  | WMI | 2,98 | 1,41 | 7 |  |  |  |  |  |  |  |
| 3 | Males | Control | 3,63 | 2,86 | 13 |  |  |  |  |  |  |  |
|  |  | WMI | 1,58 | 3,01 | 12 |  |  |  |  |  |  |  |
|  | Females | Control | 3,15 | 1,94 | 15 |  |  |  |  |  |  |  |
|  |  | WMI | 3,50 | 1,81 | 7 |  |  |  |  |  |  |  |
| 4 | Males | Control | 4,30 | 2,39 | 13 |  |  |  |  |  |  |  |
|  |  | WMI | 3,62 | 2,52 | 12 |  |  |  |  |  |  |  |
|  | Females | Control | 3,67 | 1,63 | 15 |  |  |  |  |  |  |  |
|  |  | WMI | 3,42 | 1,52 | 7 |  |  |  |  |  |  |  |
| 5 | Males | Control | 3,38 | 2,51 | 13 |  |  |  |  |  |  |  |
|  |  | WMI | 3,79 | 2,64 | 12 |  |  |  |  |  |  |  |
|  | Females | Control | 3,34 | 1,70 | 15 |  |  |  |  |  |  |  |
|  |  | WMI | 2,95 | 1,60 | 7 |  |  |  |  |  |  |  |
